# Correlation-Driven Spin-Component-Scaled Second-Order Møller–Plesset Perturbation Theory (CD-SCS-MP2)

**DOI:** 10.1021/acs.jctc.5c01167

**Published:** 2025-09-16

**Authors:** A. Paulau, L. Soriano-Agueda, E. Matito

**Affiliations:** † 226245 Donostia International Physics Center (DIPC), 20018 Donostia, Euskadi, Spain; ‡ Departamento de Física y Química Teórica, Facultad de Química, 164178 Universidad Nacional Autónoma de México, Cd. Universitaria, 04510 Ciudad de México, México; § Ikerbasque Foundation for Science, Plaza Euskadi 5, 48009 Bilbao, Euskadi, Spain; ⊥ Polimero eta Material Aurreratuak: Fisika, Kimika eta Teknologia, Kimika Fakultatea, Euskal Herriko Unibertsitatea UPV/EHU, 20080 Donostia, Euskadi, Spain

## Abstract

Møller–Plesset second-order perturbation theory (MP2) is one of the most popular and successful methods in computational chemistry, but it is not without disadvantages. It fails to capture nondynamic correlation, overestimates dispersion interactions in strongly polarizable systems, and inaccurately describes delocalized molecules. Spin-component scaling techniques improve MP2 energies by compensating for the fact that, in general, opposite-spin correlation plays a significantly greater role than same-spin correlation. On average, SCS-MP2 improves the reaction energies of small organic molecules, vibrational frequencies, thermodynamic properties, and π-stacking interactions; however, the optimal scaling values are known to be system-dependent, resulting in multiple SCS-MP2 methods. In this work, we propose improving the accuracy of SCS-MP2 by scaling the opposite-spin correlation according to the amount of dynamic correlation as measured from recently developed correlation indices that depend on the natural orbital occupations. In this way, the method is correlation-driven and can effectively adapt to the system-specific nature of spin-scaling factors. The correlation-driven SCS-MP2 (CD-SCS-MP2) method adds a negligible cost to the MP2 calculation and provides results superior to those obtained from SCS-MP2.

## Graphical abstract

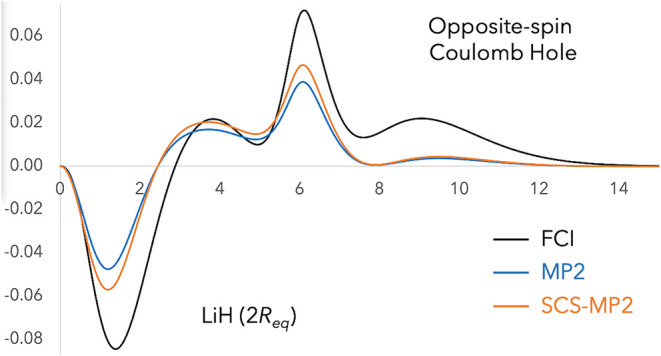

## Introduction

Møller–Plesset second-order perturbation theory (MP2)^1^ was one the first attempts to introduce electron correlation, improving the Hartree–Fock (HF) wave function. It became one of the most widely employed ab initio methods due to relatively efficient account of dynamic correlation energy compared to the relatively small computational cost increase with respect to HF. However, MP2 partially saw a decline in popularity with the appearance of modern density functional approximations (DFAs),^2–7^ which also introduce dynamic correlation and often outperform MP2 at a reduced computational cost. From this moment on, MP2 has been mostly used to overcome some now well-known failures of DFAs, such as the delocalization error,^8,9^ the description of noncovalent interactions,^10,11^ or grid-sensitivity problems.^12–19^ The local nature of (short-ranged) dynamic correlation has been also exploited to develop efficient MP2 methods, making MP2 competitive against DFAs.^20–23^ Perhaps one of the first most striking examples is the algorithm by Ochsenfeld and co-workers that was capable of computing 1000 atoms with 10,000 basis functions.^24^

MP2 is not without its disadvantages. As a single-reference method, it fails to capture nondynamic correlation.^25^ Additionally, it has other limitations, such as overestimating dispersion interactions in strongly polarizable systems^10,26,27^ and inaccurately describing delocalized molecules,^28^ including transition states.^29^ In 2003, Grimme developed a strategy to improve MP2 energies by compensating for the fact that, in general, opposite-spin correlation plays a significantly greater role than same-spin correlation. His spin-component-scaled MP2 (SCS-MP2) method increased the amount of the opposite-spin correlation (*c*_OS_) and decreased the amount of the same-spin correlation (*c*_SS_).^30^ On average, SCS-MP2 improves the reaction energies of small organic molecules, vibrational frequencies, thermodynamic properties, and π-stacking interactions.^31,32^ Grimme’s SCS-MP2 has been rationalized^33–35^ and has inspired other spin-scaled MP2 alternatives, such as a tailored SOS-MP2 for π-systems,^36^ SCS-MP2-vdW^37^ for noncovalent interactions, SCS-IL-MP2 ^38^ for ionic liquids, SCS­(MI)-MP2 ^39^ for both hydrogen bonded and dispersion complexes, the Feenberg-optimized FE2-SCS-MP2 of Szabados,^33^ SCSC-MP2,^40^ developed for catalysis, and S2opt-MP2.^41^ This proliferation of SCS-MP2 alternatives suggests that the optimal values of *c*_OS_ and *c*_SS_ are system-dependent.^34^

In this work, we propose to improve the accuracy of MP2 by scaling the opposite-spin correlation according to the amount of dynamic correlation as measured from recently developed correlation indices.^42–44^ These indices only depend on the natural orbital occupations, so they can be calculated efficiently, and are expected to account for the system-specific nature of *c*_OS_ and *c*_SS_. The correlation-driven spin-component-scaled MP2 (CD-SCS-MP2), developed and presented in this manuscript adds a negligible cost to the MP2 calculation and provides results superior to those obtained from SCS-MP2.

## Motivation

The improvement provided by MP2 over the Hartree–Fock (HF) energy arises from a more accurate description of electron correlation, achieved through a perturbative correction involving two-electron integrals. Consequently, there appears to be no explicit correction to the one-electron terms computed at the MP2 level. However, this is only because the perturbative correction is not naturally separable into one- and two-electron components. To rationalize the correlation effects captured by MP2, we adopt here the reconstruction of the MP2 one- and two-electron density matrices, commonly referred to as the relaxed first- and second-order MP2 density matrices.^45^

From the relaxed second-order MP2 density, we can construct the MP2 Coulomb hole, *h* ^MP2^(*r*_12_), which returns the difference between MP2 and HF electron–electron correlation

1 $$\Delta V_{\mathrm{ee}}^{\mathrm{MP}2}=V_{\mathrm{ee}}^{\mathrm{MP}2}-V_{\mathrm{ee}}^{\mathrm{HF}}=\int dr_{12}h^{\mathrm{MP}2}\left(r_{12}\right)/r_{12}$$

where

2 $$h^{\mathrm{MP}2}\left(r_{12}\right)=I^{\mathrm{MP}2}\left(r_{12}\right)-I^{\mathrm{HF}}\left(r_{12}\right)$$

and

3 $$I\left(r_{12}\right)=\int dr_{1}dr_{2}\delta \left(r_{12}-∥r_{1}-r_{2}∥\right)n_{2}\left(r_{1},r_{2}\right)$$

*n*_2_(**r**_1_, **r**_2_) being the corresponding second-order density (for the expression of the relaxed second-order MP2 density matrix see ref 45). We have also computed the FCI Coulomb holes, *h* ^FCI^(*r*_12_), for various systems for comparison.

In Figure 1a, we show the FCI and MP2 Coulomb holes of H_2_ at equilibrium, which, upon integration, contribute −0.070 au and −0.051 au, respectively, to the electron–electron repulsion component of the correlation energy. MP2 underestimation of electron correlation is reflected by the smaller depth of the MP2 hole compared to the FCI one. If we multiply MP2 electron correlation by 1.2, as suggested by Grimme, the contribution to the correlation energy improves to −0.061 au, in line with the improvement of the depth of the Coulomb hole. Since H_2_ involves only opposite-spin electron interactions, we turn to a more complete case by examining He_2_ (Figure 1b). The opposite-spin Coulomb hole in He_2_ behaves similarly to that of H_2_, for essentially the same reasons, supporting Grimme’s approach of scaling *E*_corr,OS_^MP2^ by a factor greater than one. The FCI same-spin Coulomb hole is very small and contributes negligibly to the overall hole, whereas the MP2-relaxed same-spin Coulomb hole exhibits positive, non-negligible values. This further justifies scaling *E*_corr,SS_^MP2^ by a small factor—0.33, as suggested by Grimme. We have tried other molecules and, in general, up-scaling the opposite-spin MP2 correlation is well justified, although not always by the same amount. However, the same-spin MP2 correlation behaves more erratically with respect to the FCI one. Let us consider the LiH molecule at both its equilibrium geometry and at twice the equilibrium bond distance. In Figure 2a, we observe that both the opposite-spin MP2 correlation and its SCS-MP2 counterpart for LiH at equilibrium underestimate the FCI reference. Meanwhile, the FCI same-spin Coulomb hole exhibits both negative and positive features that cannot be captured by simply scaling the uniformly positive MP2 same-spin Coulomb hole. The discrepancies become even more pronounced at twice the equilibrium distance, where reproducing the depth of the opposite-spin FCI Coulomb hole would require a scaling factor greater than 1.2 (see Figure 2b).

**Figure 1:**
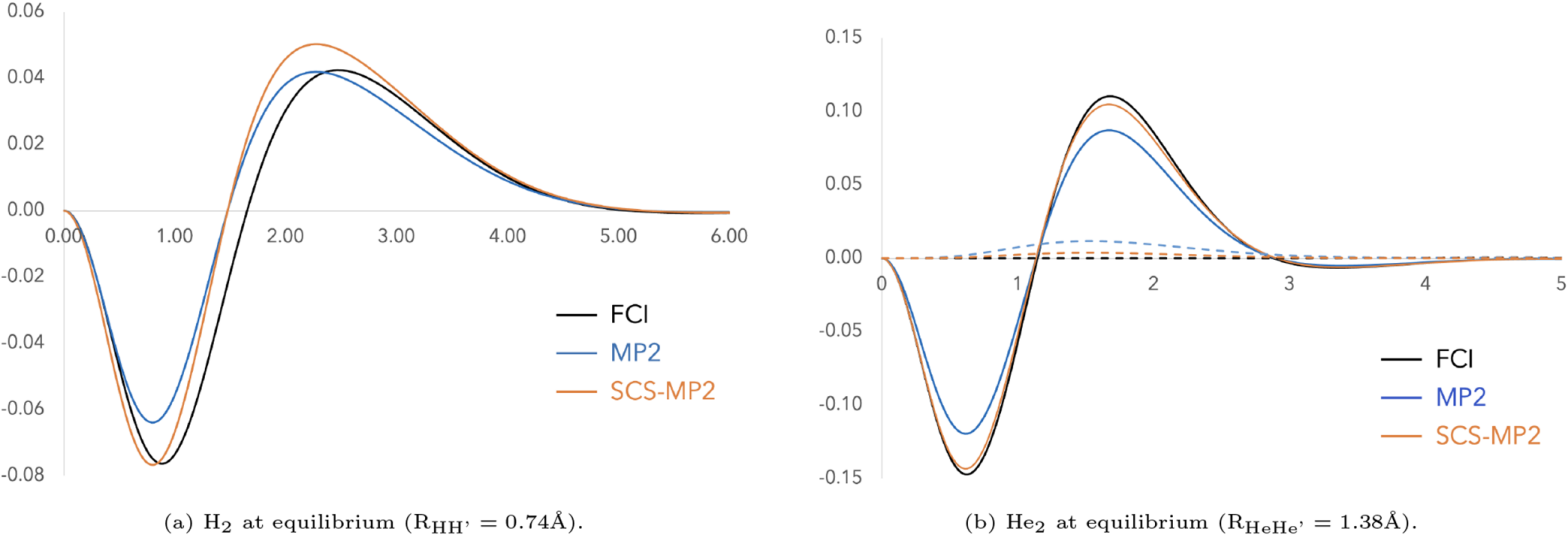
Coulomb holes for FCI, MP2 (relaxed density), and SCS-MP2 using the cc-pVTZ basis set. The solid line corresponds to the opposite-spin component and the dashed line to the same-spin one.

**Figure 2:**
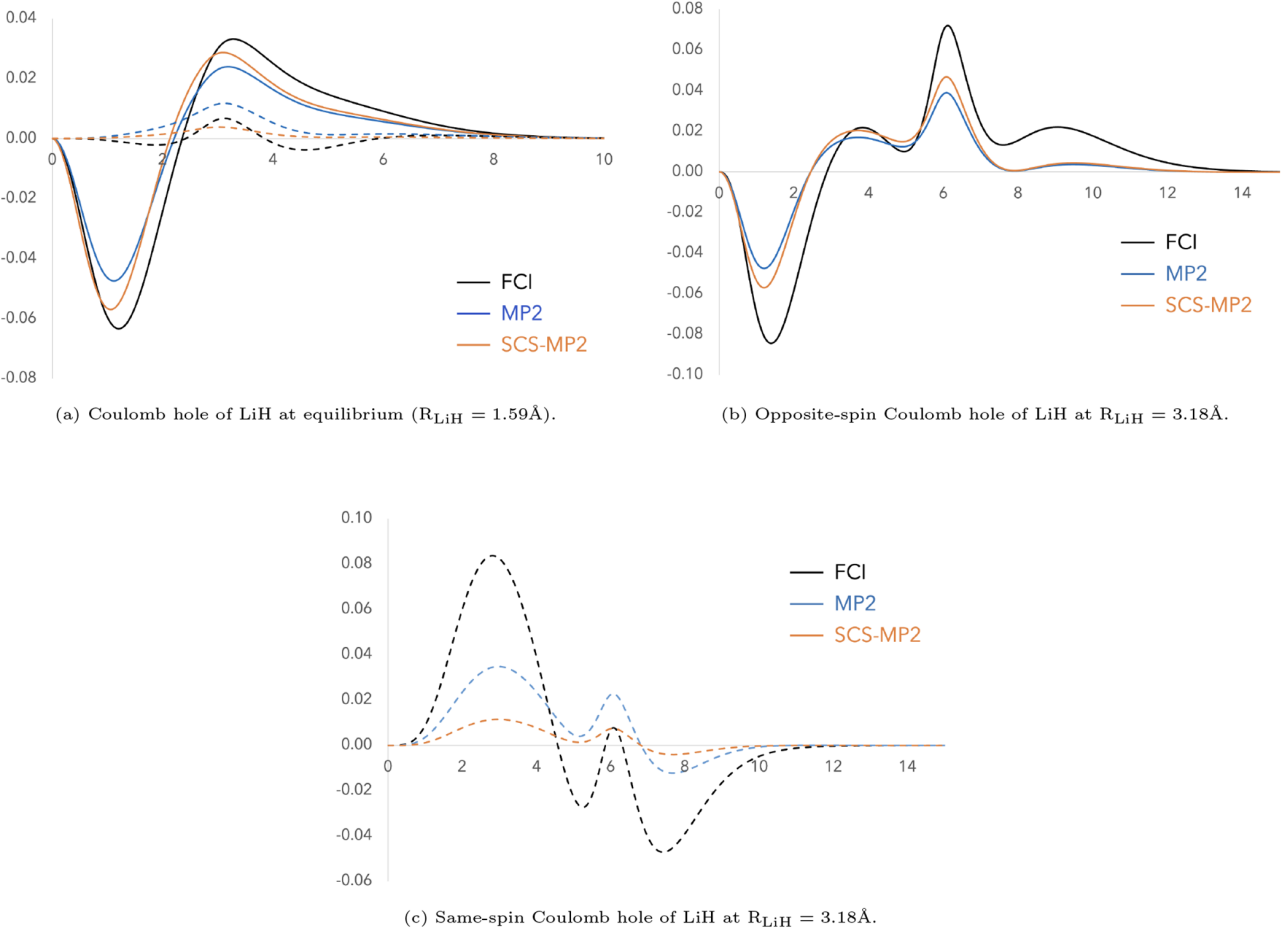
Coulomb hole of LiH at equilibrium and twice the equilibrium distance at FCI, MP2 (relaxed density), and SCS-MP2 using the cc-pVDZ basis set. The solid line corresponds to the opposite-spin component and the dashed line to the same-spin one.

Up to this point, we have focused on cases dominated by dynamic correlation, where the opposite-spin Coulomb hole outweighs the same-spin contribution. However, in the stretched geometry, LiH enters a nondynamic correlation regime, in which the same-spin component becomes comparable in magnitude to the opposite-spin one. In such a scenario, the shape of the Coulomb hole becomes significantly more complex, and a simple scaling factor is insufficient. If anything, the appropriate scaling would likely exceed unity—contrary to the typical assumptions made in SCS-MP2 approaches.

The SCS-MP2 method was not designed to compensate for MP2’s inability to describe nondynamic correlation, but rather to improve its performance in systems dominated by dynamic correlation. However, the preceding examples illustrate that the optimal scaling factors are system-dependent and, more importantly, can be effectively tuned when both the type and magnitude of electron correlation are taken into account. In particular, the amount of opposite-spin correlation can be adjusted using dynamic correlation measures, while the scaling of same-spin correlation can be guided by nondynamic correlation indicators. Such information is readily accessible from the native MP2 wave function through the electron correlation measures we have developed, based on natural orbital occupancies.^42–44,46^

MP2 correlation energy can be expressed as

4 $$E_{c}^{\mathrm{MP}2}=c_{\mathrm{OS}}E_{c}^{\mathrm{OS}}+c_{\mathrm{SS}}E_{c}^{\mathrm{SS}}$$

where *E*_c_^OS^ and *E*_*c*_^SS^ correspond to the opposite- and same-spin correlation components. In canonical MP2 theory, *c*_OS_ = *c*_SS_ = 1, whereas SCS-MP2 takes *c*_OS_ = 1.2 and *c*_SS_ = 0.33. In order to explore how correlation measures can enter *c*_OS_ and *c*_SS_, we study the series of energy differences contained in the diet-GMTKN55 set.^47^ In particular, we have computed *c*_*OS*_ and *c*_SS_ in the range [0,2.5], and displayed the results in Figure 3 for some selected systems of the diet-GMTKN55 set. This color-mapped figure shows that errors relative to the reference values are minimal along a linear region with a negative slope. In Section 2 of the Supporting Information, we highlight a few exceptions to this trend. However, these cases are outnumbered by those where *c*_OS_ and *c*_SS_ exhibit a clear linear inverse correlation.

**Figure 3:**
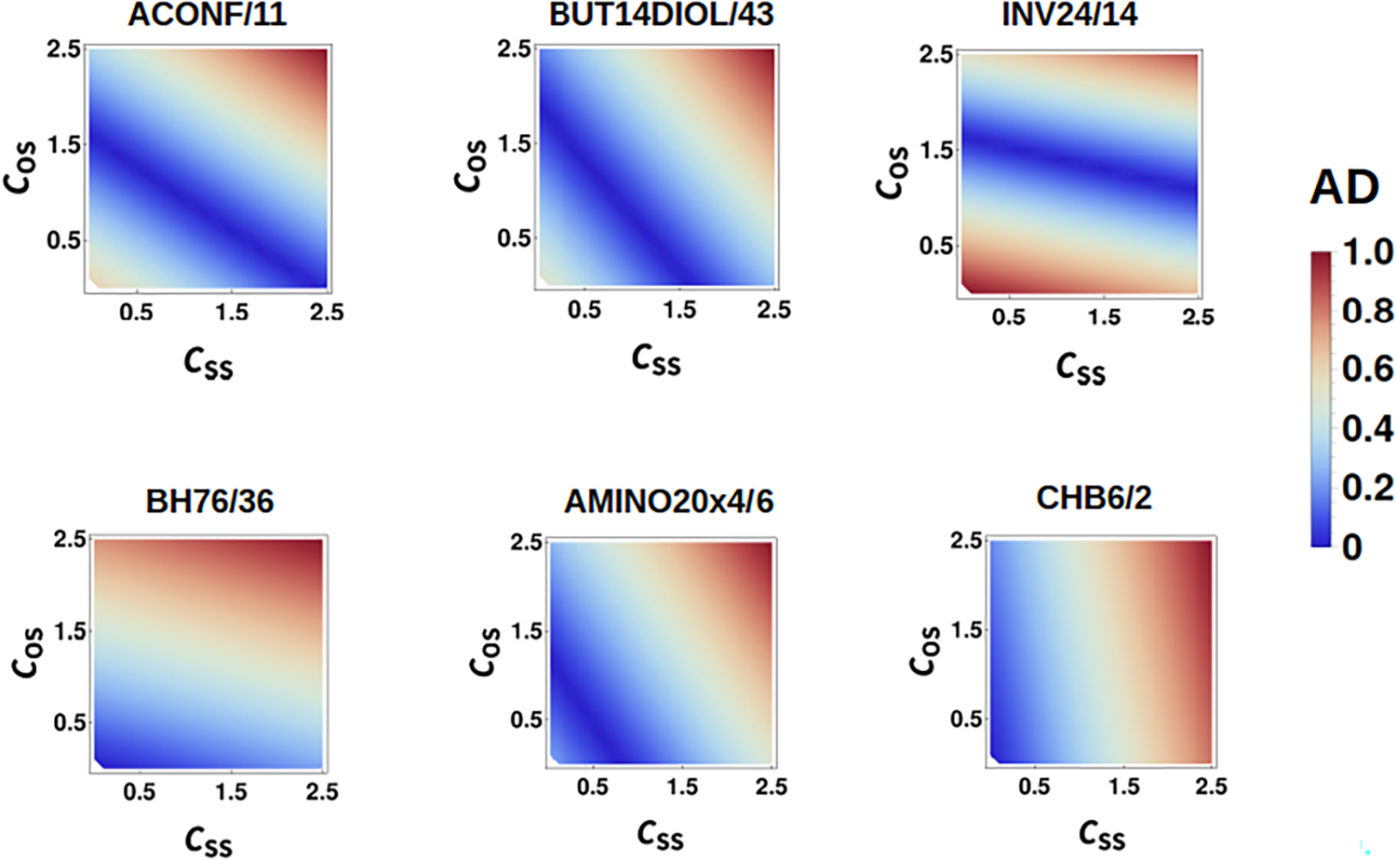
Heat maps of absolute errors for selected systems of the diet-GMTKN55 data set. ACONF/11 is the map corresponding to system 11 of the ACONF subset. The same notation is used in all cases. Color gradient in kcal/mol units. Red represents values equal to or greater than 1 kcal/mol.

## Methodology

The linear connection between *c*_OS_ and *c*_SS_ from Figure 3 suggests that both indices are inversely connected, although not necessarily through a fixed sum rule, since the slope of the line changes with the system. Since we have already determined the relationship between the opposite-spin correlation and dynamic correlation, a natural way to parametrize *c*_OS_ and *c*_SS_ using electron correlation measures is the following

5 $$c_{\mathrm{SS}}=a\frac{\overset{®}{I_{\mathrm{ND}}}}{\overset{®}{I_{T}}}+b;,c_{\mathrm{OS}}=a^{\prime }\frac{\overset{®}{I_{D}}}{\overset{®}{I_{T}}}+b^{\prime }$$

where *a*, *a*′, *b*, and *b*′ are constants to be determined, and^42,44^

6 $$\overset{®}{I_{\mathrm{ND}}}=\frac{1}{N}\sum_{i,\sigma }n_{i}^{\sigma }\left(1-n_{i}^{\sigma }\right)$$

7 $$\overset{®}{I_{D}}=\frac{1}{2N}\sum_{i,\sigma }{\left[n_{i}^{\sigma }\left(1-n_{i}^{\sigma }\right)\right]}^{1/2}-\frac{1}{N}\sum_{i,\sigma }n_{i}^{\sigma }\left(1-n_{i}^{\sigma }\right)$$

8 $$\overset{®}{I_{T}}=\overset{®}{I_{D}}+\overset{®}{I_{\mathrm{ND}}}$$

where *N* is the number of electrons and *n*_*i*_^σ^ denotes the natural orbital occupancies obtained from the MP2 first-order unrelaxed density matrix. We opt for the unrelaxed density matrix because it yields occupancies strictly within the [0, 1] range and incurs very low computational cost. In contrast, relaxed density matrices are computationally more demanding, and our tests show that the resulting correlation measures are not significantly affected by the choice of MP2 natural occupancies.

One could employ statistical learning techniques to test more complex expressions than those in eq 5 in an effort to improve performance. However, the aim of this work is to preserve a simple and intuitive connection between electron correlation and the method, enabling a straightforward interpretation of its features and limitations.

Two sets are employed for training and testing the model. For the former, we employ the 150-data-points diet-GMTKN55 data set^47^ designed by Tim Gould, whereas the testing set is the full GMTKN55 data set.^48^ See the Computational Details section at the end of the manuscript for more detailed information.

We first construct SCS-MP2*, which serves as a baseline for comparison with the two CD-SCS-MP2 models. SCS-MP2* is obtained by optimizing the parameters *b* and *b*′, while fixing *a* = *a*′ = 0. This method is analogous to the original SCS-MP2, but specifically optimized to minimize the mean absolute deviation (MAD) over the GMTKN55 benchmark set. As such, SCS-MP2* represents the best performance achievable on the GMTKN55 set when employing fixed values of *c*_OS_ and *c*_SS_ (SCS-MP2’s performance is close to SCS-MP2*’s, see Tables S2 and S3). Afterward, we construct two CD-SCS-MP2 models. The first model, CD2-SCS-MP2 sets *b* = *b*′ = 0 and optimizes *a* and *a*′ to minimize the MAD of the diet-GMTKN55 set, whereas the second model, CD4-SCS-MP2, optimizes simultaneously the four parameters. The performance of the latter two models is evaluated using the GMTKN55 set, and compared against SCS-MP2*. In addition to the MAD, the maximum mean absolute error (MAX), the root-mean-square deviation (RMSD), and the weighted mean absolute deviation (WTMAD2) are computed. The WTMAD2 is evaluated with the following equation^48^

9 $$\mathrm{WTMAD}2=\frac{1}{\sum_{i=1}^{55}N_{i}}\sum_{i=1}55N_{i}\frac{56.84\;\mathrm{kcal}/\mathrm{mol}}{{\left|\Delta E\right|}_{\mathrm{avg},i}}{\mathrm{MAD}}_{i}$$

where the MAD of each subset *i* is weighted by the energy ratio $\frac{56.84}{{\left|\Delta E\right|}_{\mathrm{avg},i}}$ and the number of data *N*_*i*_ in the set. The constant 56.84 kcal/mol is the average of |Δ*E*|_avg,*i*_ over all the sets.

The optimal coefficients of SCS-MP2* are *c*_OS_ = 1.13 and *c*_SS_ = 0.44, fairly close to the values of SCS-MP2 proposed by Grimme et al. (*c*_OS_ = 1.2 and *c*_SS_ = 0.33).^30^ The two-parameter correlation-driven SCS-MP2 method, CD2-SCS-MP2 produces the values *a*′ = 2.89 and *a* = 1.38, whereas CD4-SCS-MP2 gives the values *a* = 0.00, *a*′ = 0.42, *b*′ = 0.79, and *b* = 0.47. Interestingly, the four-parameter optimization suggests a constant contribution from the same-spin correlation, *c*_SS_ = 0.47, whereas the opposite-spin correlation, *c*_OS_ takes values in the interval [0.79, 1.21]. In Figure 4, we show the range of *c*_OS_ and *c*_SS_ that the two methods take for the molecules in the diet-GMTKN55 set, along with the fixed values used in various SCS-MP2 variants reported in the literature. Both methods agree on an increased importance of opposite-spin correlation compared to MP2, ranging from 10% to 32%, and a significantly reduced same-spin correlation, amounting to less than 60% of that in MP2 for the molecules in the diet-GMTKN55 set. Although neither model can formally reduce to SCS-MP2 (or SCS-MP2*), the individual values of *c*_OS_ and *c*_SS_ in these latter methods fall within the ranges permitted by CD2-SCS-MP2 and CD4-SCS-MP2 (only for the opposite-spin component).

**Figure 4:**
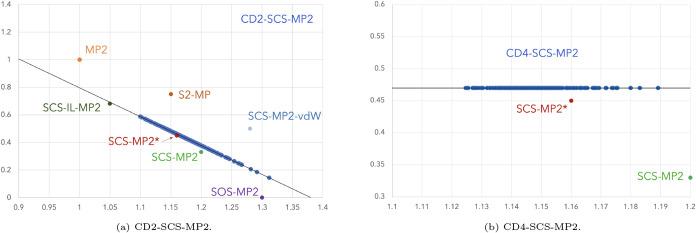
Range of *c*_OS_ (*x*-axis) and *c*_SS_ (*y*-axis) values used in various SCS-MP2 models. The solid black lines indicate the range of values allowed by the two methods developed in this article, while the blue points along the line represent the values obtained for the molecules in the diet-GMTKN55 set.

In Table 1, we give various error measures that illustrate the performance of the methods for the training set, the diet-GMTKN55 data set.^47^ The results show a clear improvement of both CD2-SCS-MP2 and CD4-SCS-MP2 models over MP2, MP3, and MP2.5.^49^ However, the magnitude of the improvement depends on the statistical measure of the error we choose to examine. Since MAD is used as the figure of merit to minimize, it is only natural that MAD is clearly improved over MP2 and SCS-MP2*, reaching values as low as 1.18 kcal/mol for CD4-SCS-MP2. However, we see a concomitant improvement of the maximal error and the root-mean-square deviation (RMSD) that suggests these methods perform much better, in general. Conversely, the weighted MAD gives a different picture, where CD-SCS-MP2 methods improve over MP2, MP3, and MP2.5 but not over SCS-MP2*. The latter is due to the larger weight that noncovalent interactions have on the WTMAD2 measure, as will be evident from the discussion we present below.

**Table 1:** Error Assessment of MP2, MP2.5, MP3, SCS-MP2*, CD2-SCS-MP2, and CD4-SCS-MP2 for the Training Set [the Diet-GMTKN55 Data Set (150 Data Points)][Bibr ref47] ^a^

	MP2	MP3	MP2.5	SCS-MP2*	CD2-SCS-MP2	CD4-SCS-MP2
MAD	3.17	3.33	2.12	2.27	1.36	1.18
MAX	37.74	37.74	37.74	26.38	22.19	14.95
RMSD	6.10	7.13	4.63	4.45	2.92	2.23
WTMAD2	6.72	6.89	6.34	5.12	5.75	5.52

a All values in kcal/mol.

## Results and Discussion

### GMTKN55 Data Set

To evaluate CD-SCS-MP2 methods, we test their performance on the GMTKN55 data set,^48^ the results of which are reported in Table 2. The comparison with Table 1 confirms the improvement of CD-SCS-MP2 methods over MP2, SCS-MP2*, and S2opt-MP2,^41^ reducing the MAD below 1.7 kcal/mol and yielding significantly smaller maximum and RMSD errors. The evaluation test further suggests that the WTMAD2 is not substantially improved compared to the best-case fixed-coefficient scenario, i.e., SCS-MP2*.

**Table 2:** GMTKN55 Data Set, Which Corresponds to 1505 Data Points^a^

error type	MP2	S2opt-MP2	SCS-MP2*	CD2-SCS-MP2	CD4-SCS-MP2
MAD	3.13	2.07	2.02	1.68	1.53
MAX	46.70	31.97	44.03	22.19	17.85
RMSD	6.29	3.98	4.07	3.15	2.67
WTMAD2	8.08	5.62	5.43	5.53	5.17

a SCS-MP2*: *c*_OS_ = 1.13, *c*_OS_ = 0.44. S2opt-MP2: *c*_OS_ = 1.055, *c*_SS_ = 0.623. CD2-SCS-MP2: *c*_OS_ = 1.38­(*I*_D_/*I*_T_) and *c*_SS_ = 2.89­(*I*_ND_/*I*_T_). CD4-SCS-MP2: *c*_OS_ = 0.42­(*I*_D_/*I*_T_) + 0.79, *c*_SS_ = 0.47. All values in kcal/mol.

A set-by-set comparison is presented in Table 3. It is clear that the largest margin of improvement over MP2—as measured by MAD and RMSD values—is observed for sets 1, 2, and 3, which include reaction energies, isomerization energies, and barrier heights. For set 1, SCS-MP2* significantly reduces all error metrics compared to MP2, and CD-SCS-MP2 models improve them further, lowering the MAD to 2.09–2.18 kcal/mol. A similar trend is found for set 2, although in this case, the CD4-SCS-MP2 method achieves a much greater reduction in error than CD2-SCS-MP2, reaching a MAD of 2.27 kcal/mol—about 1.5 kcal/mol lower than that of SCS-MP2*. This result is particularly remarkable considering that set 2 includes the MB16–43 and C60ISO subsets, which are especially challenging for electronic structure methods. The former comprises artificial molecules and gives the highest MAD across all GMTKN55 subsets for every method considered in this work. At the MP2 level, MB16–43 yields a MAD of 21.97 kcal/mol, which is reduced to 14.55 kcal/mol with SCS-MP2*. The CD-SCS-MP2 methods lower this further, down to 11.27 kcal/mol for CD2-SCS-MP2 and 7.78 kcal/mol for CD4-SCS-MP2 (see Table S8). A similar conclusion can be drawn for C60ISO, which is often the subset responsible for the second-largest error and comprises relative energies of fullerene isomers.

**Table 3:** Set 1: Basic Properties and Reaction Energies for Small Systems (475 Data Points); Set 2: Reaction Energies for Large Systems and Isomerization Reactions (243 Data Points); Set 3: Barrier Heights (194 Data Points); Set 4: Intermolecular Noncovalent Interactions (304 Data Points); Set 5: Intramolecular Noncovalent Interactions (291 Data Points)^a^

	MP2	SCS-MP2	SCS-MP2*	CD2-SCS-MP2	CD4-SCS-MP2	SCS-MP2-vdW	
Set 1	4.42	2.79	2.86	2.09	2.18	6.20	MAD
	23.39	16.44	14.88	13.41	10.05	33.92	MAX
	6.32	3.87	3.87	2.93	2.92	9.50	RMSD
	3.71	2.82	2.76	2.23	2.22	3.62	WTMAD2
Set 2	6.31	4.09	3.73	3.03	2.27	3.68	MAD
	46.70	48.19	44.03	22.19	17.85	34.13	MAX
	11.64	9.14	8.17	5.86	4.33	7.19	RMSD
	9.22	5.31	5.27	4.63	4.29	6.89	WTMAD2
Set 3	3.10	2.30	2.30	2.01	1.90	2.43	MAD
	13.20	13.78	13.52	7.02	7.66	14.20	MAX
	4.43	3.47	3.44	2.75	2.60	3.61	RMSD
	8.36	6.48	6.51	5.42	5.14	6.85	WTMAD2
Set 4	1.06	1.27	1.08	1.06	0.86	1.11	MAD
	35.81	20.98	19.01	14.54	13.31	28.85	MAX
	3.40	3.10	2.61	1.79	1.62	3.60	RMSD
	14.34	11.46	10.23	11.59	10.66	12.31	WTMAD2
Set 5	0.53	0.31	0.30	0.33	0.30	0.38	MAD
	12.63	4.47	4.87	6.52	6.00	9.59	MAX
	1.24	0.51	0.51	0.62	0.52	0.91	RMSD
	7.47	5.23	5.00	5.37	5.00	5.60	WTMAD2

a All values in kcal/mol.

Set 3 contains BH76, which includes barrier heights for hydrogen transfer, heavy atom transfer, nucleophilic substitutions, unimolecular reactions, and association reactions. This is one of the few subsets where SCS-MP2* underperforms relative to MP2—the only such case within Set 3. In contrast, both CD2-SCS-MP2 and CD4-SCS-MP2 outperform MP2 for this subset.

A completely opposite conclusion can be drawn from the analysis of Sets 4 and 5, which involve inter- and intramolecular noncovalent interactions, respectively. In these cases, there are various subsets for which MP2 outperforms both SCS-MP2* and CD-SCS-MP2 methods. Although MP2 generally yields larger overall errors than spin-component-scaled methods, this is primarily due to very large errors in binding energies for halogenated dimers and systems dominated by intramolecular dispersion. While SCS-MP2*, and especially CD-SCS-MP2, reduce the MAD in these subsets, the improvement is insufficient to significantly lower the overall WTMAD2 values for Sets 4 and 5, which remain around 10 and 5 kcal/mol, respectively. Not even SCS-MP2-vdW, which was specifically designed for van der Waals interactions, is able to improve these values. Martin et al.^50^ do not recommend the use of MP2 or SCS-MP2 for halogen-bonded compounds, while Radom et al.^51^ specifically discourage their use for water clusters. These concerns are echoed in the performance of the WATER27 and HAL59 subsets, which yield some of the largest errors in Set 4 (see Tables S8–S11).

This discussion raises the question of how strongly the performance of spin-scaled methods is affected by their inaccurate description of noncovalent interactions, which appears to be a key limiting factor. In Table 4, we present the overall error statistics for the GMTKN55 set, excluding Sets 4 and 5. As we can see, all methods give worse MAD and RMSD values (as expected, since noncovalent interactions have lower magnitudes than e.g., energy barriers). Unlike MP2 or SCS-MP2, CD-SCS-MP2 increases these values by less than 1 kcal/mol with respect to the statistics of the full GMTKN55 data set. More notable, the WTMAD2 values of CD-SCS-MP2 methods are now clearly below the SCS-MP2* value, highlighting the overall improved performance of CD-SCS-MP2 over SCS-MP2, which were hindered by a general poor performance of these methods for noncovalent interactions. In fact, if we discard data points involving molecules suspicious of presenting multireference character (we discard systems with molecules presenting *I*_ND_^max^ > 0.030)^43^ both CD-SCS-MP2 methods achieve MAD below 2 kcal/mol, and WTMAD2 values as low as 3.15 kcal/mol, significantly lower than both MP2 and SCS-MP2* (see Table 4).

**Table 4:** Set A: GMTKN55 Data Set without Sets 4 and 5, Which Corresponds to 910 Data Points, and Set B: Set A Discarding Also Molecules with *I*_ND_^max^ > 0.030 (882 Data Points)^43^ ^a^

	MP2	SCS-MP2*	CD2-SCS-MP2	CD4-SCS-MP2	
Set A	4.65	2.97	2.32	2.18	MAD
	46.70	44.03	22.19	17.85	MAX
	7.82	5.30	3.90	3.43	RMSD
	6.86	4.70	4.00	3.80	WTMAD2
Set B	3.18	2.52	1.87	1.74	MAD
	22.60	12.46	9.17	8.85	MAX
	4.78	3.50	2.61	2.54	RMSD
	5.74	4.98	3.32	3.15	WTMAD2

a SCS-MP2*: *c*_OS_ = 1.13, *c*_OS_ = 0.44. CD2-SCS-MP2: *c*_OS_ = 1.38­(*I*_D_/*I*_T_) and *c*_SS_ = 2.89­(*I*_ND_/*I*_T_). CD4-SCS-MP2: *c*_OS_ = 0.42­(*I*_D_/*I*_T_) + 0.79, *c*_SS_ = 0.47. All values in kcal/mol.

Various authors have found that bonded and nonbonded interactions call for quite different scaling coefficients^31,36,37,39,52,53^ and this is not something we are capturing well with natural-orbital-based correlation measures.^42^ To rationalize why noncovalent interactions are so poorly represented, we revisit the Coulomb hole of the helium dimer (for which an FCI reference hole can be easily generated for comparison), this time analyzing its long-range behavior, which was omitted from Figure 1b for clarity. In particular, some of our recent results have linked the long-range part of the cumulant component of the Coulomb hole (*c*_II_ component)^54,55^ to the ability of electronic structure methods to capture dispersion interactions. Its energetic counterpart—the integral of the long-range portion of the Coulomb hole weighted by 1/*r*_12_—has been associated with the dispersion energy.^54–56^

The opposite-spin Coulomb hole at short-range (see Figure 1b) is well approximated by SCS-MP2 and the long-range part connected to dispersion (shown in Figure 5a) remains only slightly underestimated. However, applying a 0.33 scaling factor to the same-spin component, further worsens the underestimation of dispersion interactions in the He dimer (shown in Figure 5b), which was already slightly underestimated at the MP2 level. For the He dimer, MP2 underestimates the dispersion energy, but if we faced any of the multiple situations in which MP2 overestimates dispersion (e.g., for strongly polarizable systems) the situation would not be much different. Since short-range correlation contributes far more significantly to the total correlation energy than long-range correlation, any energy-optimized value *c*_OS_ will inevitably be dominated by the coefficient best suited for capturing short-range dynamic correlation—which may not align with the optimal value required at long-range. Conversely, the value of *c*_SS_ may not be strongly influenced by dynamic correlation; however, it is unlikely that *c*_SS_ can be used to correct MP2’s poor treatment of dispersion interactions, as this coefficient is effectively governed by the extent of nondynamic correlation. Unless one corrects for correlation differently at different interelectronic ranges, it is not possible to simultaneously address both the underestimation of dynamic short-range correlation and long-range dispersion within a single scaling scheme. Since such range-dependent scaling is not part of the SCS-MP2 framework—whether correlation-driven or not—this constitutes a fundamental limitation of this family of methods. Head-Gordon and co-workers have addressed this problem using attenuated MP2 methods with a long-range dispersion correction for treating nonbonded interactions.^57^ We are currently working in our laboratory to construct a range-separated version of CD-SCS-MP2 that could attenuate this problem without resorting to dispersion corrections.

**Figure 5:**
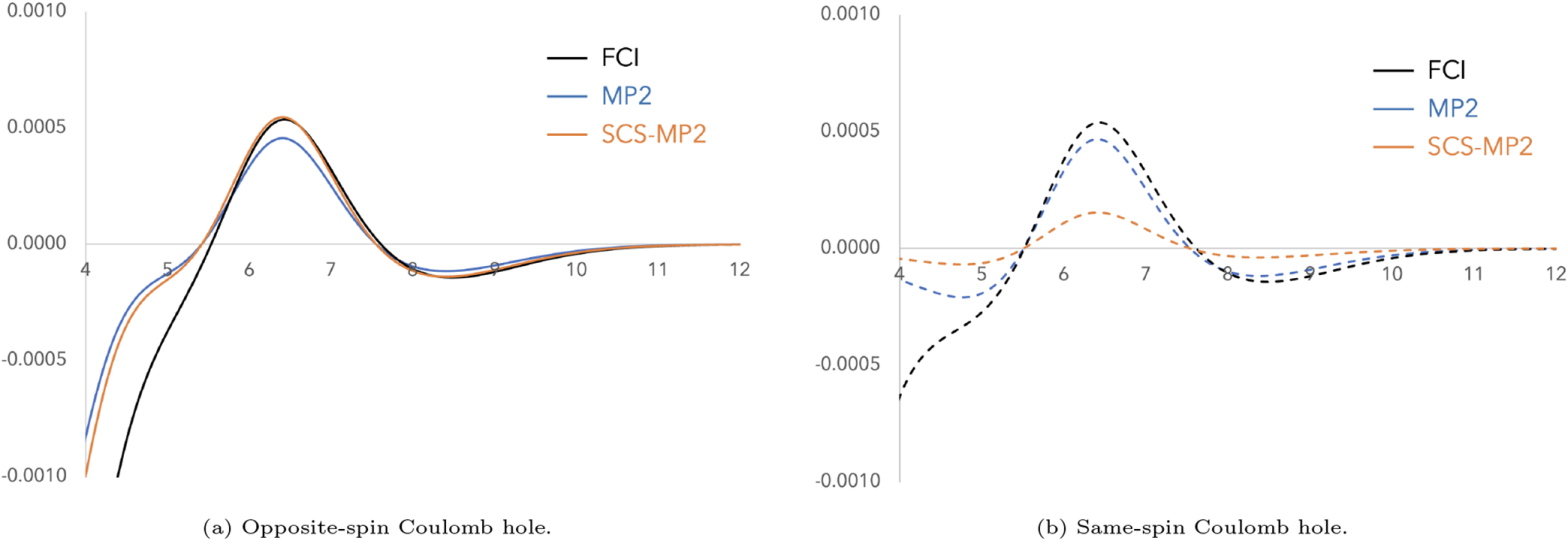
Coulomb holes of He_2_ dimer for FCI, MP2 (relaxed density), and SCS-MP2 using the cc-pVTZ basis set. The solid line corresponds to the opposite-spin component and the dashed line to the same-spin one.

#### CD-SCS-MP2 vs DFT

In this section, we assess the competitiveness of CD-SCS-MP2 against Kohn–Sham DFT. To this end, we selected some density functional approximations (DFAs) widely used by the scientific community: PBE-D3­(BJ),^58,59^ B3LYP-D3­(BJ)^60–62^ and M06–2X-D3(0).^63,64^ We also included the range-separated hybrid ωB97X-D3­(BJ)^65^ and the double-hybrid DSD-PBEP86-D3­(BJ)^66^ – which is ranked as the DFA most frequently scoring among the best performing (see Table S14 in ref 48). One should consider that the number of optimized parameters in most DFAs is notoriously larger to the two or four parameters included in SCS-MP2 or CD-SCS-MP2 methods (PBE and other nonempirical functionals being an exception). However, we believe this comparison sheds some light on the competitiveness of MP2 methods against DFT. The results are collected in Table 5. Data on many additional DFAs can be found in the Supporting Information (SI), including some nonempirical density functional approximations^67,68^ (see Table S12 and Figures S3–S22).

**Table 5:** Set 1: Basic Properties and Reaction Energies for Small Systems (475 Data Points); Set 2: Reaction Energies for Large Systems and Isomerization Reactions (243 Data Points); Set 3: Barrier Heights (194 Data Points); Set 4: Intermolecular Noncovalent Interactions (304 Data Points); Set 5: Intramolecular Noncovalent Interactions (291 Data Points)^a^

	CD2-SCS-MP2	CD4-SCS-MP2	PBE-D3(BJ)	B3LYP-D3(BJ)	M06–2X-D3(0)	ωB97X-D3(0)	DSD-PBEP86-D3(BJ)	
Set 1	2.09	2.18	6.00	4.22	3.02	3.55	1.87	MAD
	13.41	10.05	54.18	40.25	22.39	30.87	11.45	Max(MAD)
	2.93	2.92	7.57	5.16	3.95	4.44	2.36	RMSD
	2.23	2.22	6.51	4.36	2.73	3.32	1.69	WTMAD2
Set 2	3.03	2.27	5.97	5.55	3.70	6.86	2.23	MAD
	22.19	17.85	72.41	19.81	37.90	20.82	15.3	Max(MAD)
	5.86	4.33	7.43	6.58	4.54	7.92	3.79	RMSD
	4.63	4.29	12.36	10.28	5.84	7.85	3.91	WTMAD2
Set 3	2.01	1.90	7.01	2.59	1.94	1.58	1.53	MAD
	7.02	7.66	9.15	16.19	44.30	7.12	9.80	Max(MAD)
	2.75	2.60	7.57	3.02	3.03	1.96	1.80	RMSD
	5.42	5.14	18.36	9.04	4.99	4.67	3.52	WTMAD2
Set 4	1.06	0.86	1.48	0.80	0.74	0.61	0.53	MAD
	14.54	13.31	30.19	13.89	9.98	7.41	8.11	Max(MAD)
	1.79	1.62	1.94	1.03	0.91	0.77	0.66	RMSD
	11.59	10.66	10.21	5.56	5.20	4.54	4.25	WTMAD2
Set 5	0.33	0.30	0.78	0.68	0.61	0.57	0.35	MAD
	6.52	6.00	9.08	12.38	4.82	5.52	2.54	Max(MAD)
	0.62	0.52	1.04	0.97	0.76	0.73	0.41	RMSD
	5.37	5.00	9.58	5.68	7.48	4.86	3.46	WTMAD2

a The data from PBE-D3­(BJ), B3LYP-D3­(BJ), M06-2X-D3(0), ωB97X-D3(0), and DSD-PBEP86-D3­(BJ) were taken from ref 69. All values in kcal/mol.

For Sets 1 and 2, both CD-SCS-MP2 methods clearly outperform all DFAs except DSD-PBEP86-D3­(BJ), which performs slightly better than CD4-SCS-MP2. In the case of Set 1, for all DFAs, the largest MAD and RMSD arise from the SIE4x4 subset,^48^ which includes systems prone to self-interaction errors. For MP2, the largest errors originate from the W4–11 subset, which comprises atomization energies (see Tables S8–S11). In Set 2, the MB16–43 and C60ISO subsets present the largest errors across all methods.

Set 3 is composed of barrier heights, which were included in the training of ωB97X to improve the performance on kinetic data, which was a limitation in earlier versions of the functional. Hence, it is not entirely surprising that this functional, as well as DSD-PBEP86-D3­(BJ), outperforms CD-SCS-MP2.

Set 4 consists of intermolecular noncovalent interactions. Since all DFAs considered have been empirically corrected for dispersion using the D3 scheme,^70^ they all outperform CD-SCS-MP2 on this set, with the exception of PBE-D3­(BJ). Although the zero-damping scheme, D3(0),^70^ seems to provide lower errors, the lowest error is actually obtained for the double-hybrid corrected with Becke–Johnson (BJ) damping function.^71^ Conversely, Set 5 focuses on intramolecular noncovalent interactions, which are more challenging to model with dispersion-corrected functionals. In this case, only DSD-PBEP86-D3­(BJ) clearly outperforms CD-SCS-MP2.

## Conclusions

We have motivated the use of a correlation-driven spin-component-scaled MP2 method (CD-SCS-MP2), which adjusts the contributions of opposite- and same-spin correlation according to the amounts of dynamic and nondynamic correlation present in the system. This approach outperforms the best fixed-coefficient SCS-MP2 method (and the native SCS-MP2) at a negligible additional computational cost (the computation of natural orbital occupancies).

We have designed a two-parameter CD-SCS-MP2, CD2-SCS-MP2, and a four-parameter counterpart, CD4-SCS-MP2 and demonstrated numerically, using the GMTKN55 data set, that both clearly outperform MP2, SCS-MP2, and most pure and hybrid DFAs in reaction energies, isomerization reactions, and barrier heights. Only ωB97X-D3(0) performs slightly better than CD-SCS-MP2 for barrier heights.

On the other hand, neither SCS-MP2 nor CD-SCS-MP2 perform well for noncovalent interactions, showing clear inferiority compared to empirically corrected DFAs in the case of intermolecular noncovalent interactions. Through the analysis of Coulomb holes, we have traced this deficiency to the different range performance of MP2, which cannot simultaneously capture short-range dynamic correlation and long-range dispersion interactions through uniform spin-component scaling. A promising solution to this issue may lie in a range-separated version of CD-SCS-MP2, which is currently under investigation in our laboratory.

The current methodology can be readily extended to double-hybrid DFAs, although this requires correlation measures that are compatible with the DFT framework. Given the promising results reported for DSD-PBEP86-D3­(BJ), such extensions may pave the way toward chemically accurate methods and are also currently being explored in our group.

## Computational Details

The optimal parameters of CD-SCS-MP2 were trained from the relative energies of the diet-GMTKN55 ^47^ data set, which contains 337 molecules corresponding to 150 relative energies (data points), whereas the performance of the method was evaluated using the whole GMTKN55 data set.^48^ The GMTKN55 set contains 2561 molecules, corresponding to 1505 chemical and physical processes. This data set contains 5 subsets: (i) basic properties, (ii) reaction and isomerization energies, (iii) reaction barriers heights, (iv) intermolecular noncovalent interactions and (v) intramolecular noncovalent interactions.

For molecules within the diet-GMTKN55 and GMTKN55 data sets, single-point calculations were carried out at the MP2 ^1^ level of theory with the def2-qzvp basis set.^72^ In the case of the WATER27 subset, the basis set was increased with diffuse **s** and **p** functions for oxygen atoms.^73^ Diffuse **s** and **p** functions were applied to all non-hydrogen atoms in subsets G21EA, AHB21 and IL16, while diffuse **s** functions were applied to hydrogen atoms. Core electrons of heavy elements in some systems of HEAVY28, HEAVYSB11 and HAL59 were replaced with the def2-ECP effective-core-potentials.^74^ All calculations were performed using the ORCA v.4.2.1 software package,^75,76^ except the MP3 calculations that were performed with Gaussian16.^77^ The heat maps were generated with the Mathematica v.12 software.^78^

The code used to compute $\overset{®}{I_{\mathrm{ND}}}/\overset{®}{I_{T}}$ and $\overset{®}{I_{D}}/\overset{®}{I_{T}}$ indices is available in Section 1 of the SI. It also explains step by step how to execute the program using the outputs generated by the ORCA v.4.2.1 computational package. The program RHO2_OPS^79^ was employed to compute the Coulomb holes using the algorithm of Cioslowski and Liu.^80^

## Supplementary Material



## Notes

Guide to obtain optimal *c*_OS_ and *c*_SS_, heat maps for the SCS-MP2 performance in the diet-GMTKN55 data set for variable *c*_OS_ and *c*_SS_, GMTKN55 data set description, comparison of SCS-MP2* and SCS-MP2, and statistical data of the errors of CD-SCS-MP2 and a large collection of density functional approximations for the GMTKN55 data set (PDF)

## References

[1] Møller C., Plesset M. S. (1934). Note on an approximation
treatment for many-electron
systems. Phys. Rev..

[2] Becke A. D. (1993). Density-functional
thermochemistry. III. The role of exact exchange. J. Chem. Phys..

[3] Tao J., Perdew J. P., Staroverov V. N., Scuseria G. E. (2003). Climbing the density
functional ladder: Nonempirical meta-generalized gradient approximation
designed for molecules and solids. Phys. Rev.
Lett..

[4] Zhao Y., Truhlar D. G. (2008). The M06 suite of
density functionals for main group
thermochemistry, thermochemical kinetics, noncovalent interactions,
excited states, and transition elements: two new functionals and systematic
testing of four M06-class functionals and 12 other functionals. Theor. Chem. Acc..

[5] Chai J.-D., Head-Gordon M. (2008). Long-range corrected hybrid density
functionals with
damped atom-atom dispersion corrections. Phys.
Chem. Chem. Phys..

[6] Becke A. D. (2014). Perspective:
Fifty years of density-functional theory in chemical physics. J. Chem. Phys..

[7] Burke K. (2012). Perspective
on density functional theory. J. Chem. Phys..

[8] Cohen A. J., Mori-Sánchez P., Yang W. (2008). Insights into current limitations
of density functional theory. Science.

[9] Mori-Sánchez P., Cohen A. J., Yang W. (2008). Localization
and delocalization errors
in density functional theory and implications for band-gap prediction. Phys. Rev. Lett..

[10] Tkatchenko A., DiStasio R. A., Head-Gordon M., Scheffler M. (2009). Dispersion-corrected
Møller-Plesset second-order perturbation theory. J. Chem. Phys..

[11] Cremer D. (2011). Møller-Plesset
perturbation theory: from small molecule methods to methods for thousands
of atoms. WIREs. Comput. Mol. Sci..

[12] Johnson E. R., Wolkow R. A., DiLabio G. A. (2004). Application
of 25 density functionals
to dispersion-bound homomolecular dimers. Chem.
Phys. Lett..

[13] Johnson E. R., Becke A. D., Sherill C. D., DiLabio G. A. (2009). Oscillations in
meta-generalized-gradient approximation potential energy surfaces
for dispersion-bound complexes. J. Chem. Phys..

[14] Zaleśny R., Medved’ M., Sitkiewicz S. P., Matito E., Luis J. M. (2019). Can density
functional theory be trusted for high-order electric properties? The
case of hydrogen-bonded complexes. J. Chem.
Theory Comput..

[15] Sitkiewicz S. P., Zaleśny R., Ramos-Cordoba E., Luis J. M., Matito E. (2022). How reliable
are modern density functional approximations to simulate vibrational
spectroscopies?. J. Phys. Chem. Lett..

[16] Lehtola S., Marques M. A. (2022). Many recent density
functionals are numerically ill-behaved. J.
Chem. Phys..

[17] Sitkiewicz S. P., Matito E., Luis J. M., Zaleśny R. (2023). Pitfall in
simulations of vibronic TD-DFT spectra: Diagnosis and assessment. Phys. Chem. Chem. Phys..

[18] Lehtola S. (2023). Atomic electronic
structure calculations with Hermite interpolating polynomials. J. Phys. Chem. A.

[19] Sitkiewicz S. P., Ferradás R. R., Ramos-Cordoba E., Zaleśny R., Matito E., Luis J. M. (2024). Spurious oscillations
caused by density
functional approximations: Who is to blame? Exchange or correlation?. J. Chem. Theory Comput..

[20] Weigend F., Häser M. (1997). RI-MP2: first derivatives and global
consistency. Theor. Chim. Acta.

[21] Saebø S., Pulay P. (2001). A low-scaling method
for second order Møller-Plesset calculations. J. Chem. Phys..

[22] Kossmann S., Neese F. (2010). Efficient structure optimization with second-order many-body perturbation
theory: The RIJCOSX-MP2 method. J. Chem. Theory
Comput..

[23] Nagy P. R., Samu G., Kállay M. (2016). An integral-direct
linear-scaling
second-order Møller-Plesset approach. J.
Chem. Theory Comput..

[24] Doser B., Lambrecht D. S., Kussmann J., Ochsenfeld C. (2009). Linear-scaling
atomic orbital-based second-order Møller-Plesset perturbation
theory by rigorous integral screening criteria. J. Chem. Phys..

[25] Hirao K. (1992). Multireference
Møller—Plesset method. Chem. Phys.
Lett..

[26] Sinnokrot M. O., Valeev E. F., Sherrill C. D. (2002). Estimates of the ab initio limit
for *π*–*π* interactions:
The benzene dimer. J. Am. Chem. Soc..

[27] Hobza P., Selzle H. L., Schlag E. W. (1996). Potential
energy surface for the
benzene dimer. Results of ab initio CCSD (T) calculations show two
nearly isoenergetic structures: T-shaped and parallel-displaced. J. Phys. Chem. A.

[28] Kurlancheek W., Lochan R., Lawler K., Head-Gordon M. (2012). Exploring
the competition between localization and delocalization of the neutral
soliton defect in polyenyl chains with the orbital optimized second
order opposite spin method. J. Chem. Phys..

[29] Linder M., Brinck T. (2013). On the method-dependence of transition state asynchronicity
in Diels-Alder reactions. Phys. Chem. Chem.
Phys..

[30] Grimme S. (2003). Improved second-order
Møller-Plesset perturbation theory by separate scaling of parallel-and
antiparallel-spin pair correlation energies. J. Chem. Phys..

[31] Grimme S. (2005). Accurate calculation
of the heats of formation for large main group compounds with spin-component
scaled MP2 methods. J. Phys. Chem. A.

[32] Antony J., Grimme S. (2007). Is spin-component scaled
second-order Møller-
Plesset perturbation theory an appropriate method for the study of
noncovalent interactions in molecules?. J. Phys.
Chem. A.

[33] Szabados Á. (2006). Theoretical
interpretation of Grimme’s spin-component-scaled second order
Møller-Plesset theory. J. Chem. Phys..

[34] Fink R. F. (2010). Spin-component-scaled
Møller–Plesset (SCS-MP) perturbation theory: A generalization
of the MP approach with improved properties. J. Chem. Phys..

[35] Grimme S., Goerigk L., Fink R. F. (2012). Spin-component-scaled
electron correlation
methods. WIREs, Comput. Mol. Sci..

[36] Jung Y., Head-Gordon M. (2006). A fast correlated
electronic structure method for computing
interaction energies of large van der Waals complexes applied to the
fullerene-porphyrin dimer. Phys. Chem. Chem.
Phys..

[37] King R. A. (2009). On the
accuracy of spin-component-scaled perturbation theory (SCS-MP2) for
the potential energy surface of the ethylene dimer. Mol. Phys..

[38] Rigby J., Izgorodina E. I. (2014). New SCS- and SOS-MP2 Coefficients
Fitted to Semi-Coulombic
Systems. J. Chem. Theory Comput..

[39] Distasio
JR R. A., Head-Gordon M. (2007). Optimized
spin-component scaled second-order Møller-Plesset perturbation
theory for intermolecular interaction energies. Mol. Phys..

[40] Zhao Y., Truhlar D. G. (2009). Benchmark energetic
data in a model system for Grubbs
II metathesis catalysis and their use for the development, assessment,
and validation of electronic structure methods. J. Chem. Theory Comput..

[41] Martin J. M. L., Santra G. (2020). Empirical double-hybrid density functional theory:
A ‘third way’in between WFT and DFT. Isr. J. Chem..

[42] Ramos-Cordoba E., Salvador P., Matito E. (2016). Separation of dynamic and nondynamic
correlation. Phys. Chem. Chem. Phys..

[43] Xu X., Soriano-Agueda L., López X., Ramos-Cordoba E., Matito E. (2024). An All-Purpose Measure
of Electron Correlation for
Multireference Diagnostics. J. Chem. Theory
Comput..

[44] Xu X., Soriano-Agueda L., López X., Ramos-Cordoba E., Matito E. (2025). How many distinct and
reliable multireference diagnostics
are there?. J. Chem. Phys..

[45] Cioslowski J., Liu G. (1998). Electron intracule densities and Coulomb holes from energy-derivative
two-electron reduced density matrices. J. Chem.
Phys..

[46] Ramos-Cordoba E., Matito E. (2017). Local Descriptors
of dynamic and nondynamic correlation. J. Chem.
Theory Comput..

[47] Gould T. (2018). ‘Diet
GMTKN55’ offers accelerated benchmarking through a representative
subset approach. Phys. Chem. Chem. Phys..

[48] Goerigk L., Hansen A., Bauer C., Ehrlich S., Najibi A., Grimme S. (2017). A look at the density
functional theory zoo with the
advanced GMTKN55 database for general main group thermochemistry,
kinetics and noncovalent interactions. Phys.
Chem. Chem. Phys..

[49] Pitoňák M., Neogrády P., Černỳ J., Grimme S., Hobza P. (2009). Scaled MP3
non-covalent interaction energies agree closely with accurate CCSD
(T) benchmark data. Comput. Phys. Commun..

[50] Kozuch S., Martin J. M. L. (2013). Halogen Bonds:
Benchmarks and Theoretical Analysis. J. Chem.
Theory Comput..

[51] Karton A., O’Reilly R. J., Chan B., Radom L. (2012). Determination of Barrier
Heights for Proton Exchange in Small Water, Ammonia, and Hydrogen
Fluoride Clusters with G4­(MP2)-Type, MPn, and SCS-MPn Procedures—A
Caveat. J. Chem. Theory Comput..

[52] Gerenkamp M., Grimme S. (2004). Spin-component scaled
second-order Møller-Plesset
perturbation theory for the calculation of molecular geometries and
harmonic vibrational frequencies. Chem. Phys.
Lett..

[53] Hyla-Kryspin I., Grimme S. (2004). Comprehensive study of the thermochemistry of first-row
transition metal compounds by spin component scaled MP2 and MP3 methods. Organometallics.

[54] Via-Nadal M., Rodríguez-Mayorga M., Ramos-Cordoba E., Matito E. (2019). Singling out Weak and Strong Correlation. J. Phys. Chem. Lett..

[55] Via-Nadal M., Rodríguez-Mayorga M., Ramos-Cordoba E., Matito E. (2022). Range Separation of the Coulomb Hole. J. Chem. Phys..

[56] Via-Nadal M., Rodríguez-Mayorga M., Matito E. (2017). A Salient Signature
of van der Waals Interactions. Phys. Rev. A.

[57] Goldey M. B., Belzunces B., Head-Gordon M. (2015). Attenuated MP2 with a long-range
dispersion correction for treating nonbonded interactions. J. Chem. Theory Comput..

[58] Perdew J. P., Burke K., Ernzerhof M. (1996). Generalized
Gradient Approximation
Made Simple. Phys. Rev. Lett..

[59] Perdew J. P., Burke K., Ernzerhof M. (1997). Generalized
Gradient Approximation
Made Simple. Phys. Rev. Lett..

[60] Lee C., Yang W., Parr R. G. (1988). Development
of the Colle-Salvetti
correlation-energy formula into a functional of the electron density. Phys. Rev. B.

[61] Becke A. D. (1988). Density-functional
exchange-energy approximation with correct asymptotic behavior. Phys. Rev. A.

[62] Becke A. D. (1996). Density-functional
thermochemistry. IV. A new dynamical correlation functional and implications
for exact-exchange mixing. J. Chem. Phys..

[63] Zhao Y., Truhlar D. G. (2008). Density Functionals
with Broad Applicability in Chemistry. Acc.
Chem. Res..

[64] Zhao Y., Truhlar D. G. (2008). The M06 suite of
density functionals for main group
thermochemistry, thermochemical kinetics, noncovalent interactions,
excited states, and transition elements: two new functionals and systematic
testing of four M06-Class functionals and 12 other functionals. Theor. Chem. Acc..

[65] Chai J.-D., Head-Gordon M. (2008). Systematic optimization of long-range
corrected hybrid
density functionals. J. Chem. Phys..

[66] Kozuch S., Martin J. M. L. (2011). DSD-PBEP86: in search of the best double-hybrid DFT
with spin-component scaled MP2 and dispersion corrections. Phys. Chem. Chem. Phys..

[67] Brémond E., Adamo C. (2011). Seeking for parameter-free double-hybrid functionals: The PBE0-DH
model. J. Chem. Phys..

[68] Brémond É., Sancho-García J. C., Pérez-Jiménez Á. J., Adamo C. (2014). Communication: Double-hybrid functionals from adiabatic-connection:
The QIDH model. J. Chem. Phys..

[69] Goerigk L., Hansen A., Bauer C., Ehrlich S., Najibi A., Grimme S. (2017). A look At the density functional theory zoo with the
advanced GMTKN55 database for general main group thermochemistry,
kinetics and noncovalent interactions. Phys.
Chem. Chem. Phys..

[70] Grimme S., Antony J., Ehrlich S., Krieg H. (2010). A consistent and accurate
ab initio parametrization of density functional dispersion correction
(DFT-D) for the 94 elements H-Pu. J. Chem. Phys..

[71] Becke A. D., Johnson E. R. (2005). A density-functional model of the dispersion interaction. J. Chem. Phys..

[72] Weigend F., Furche F., Ahlrichs R. (2003). Gaussian basis
sets of quadruple
zeta valence quality for atoms H-Kr. J. Chem.
Phys..

[73] Kendall R.
A., Dunning T. H., Harrison R. J. (1992). Electron affinities of the first-row
atoms revisited. Systematic basis sets and wave functions. J. Chem. Phys..

[74] Weigend F., Ahlrichs R. (2005). Balanced basis sets of split valence, triple zeta valence
and quadruple zeta valence quality for H to Rn: Design and assessment
of accuracy. Phys. Chem. Chem. Phys..

[75] Neese F. (2012). The ORCA program
system. WIREs Comput. Mol. Sci..

[76] Neese F. (2018). Software update:
the ORCA program system, version 4.0. WIREs
Comput. Mol. Sci..

[77] Frisch, M. J. ; Trucks, G. W. ; Schlegel, H. B. Gaussian 16, Revision C.01; Gaussian Inc: Wallingford CT, 2016.

[78] Wolfram Research, Inc. Mathematica, Version 12.0; Wolfram Research, Inc: Champaign, IL, 2019.

[79] Rodríguez-Mayorga, M. ; RHO2-OPS: 2-DM Operations . Institute of Computational Chemistry and Catalysis; University of Girona: Catalonia, Spain; 2016, https://github.com/marm314/rho2_ops.

[80] Cioslowski J., Liu G. (1996). Fast evaluation of
electron intracule and extracule densities on
large grids of points. J. Chem. Phys..

