# Supplementary material for: Correlation-Driven Spin-Component-Scaled Second-Order Møller–Plesset Perturbation Theory (CD-SCS-MP2)
Source: J Chem Theory Comput. 2025 Sep 16;21(19):9601–11. doi: 10.1021/acs.jctc.5c01167 (PMC12529911; doi:10.1021/acs.jctc.5c01167)
Supplement: Supplementary file 1 [file ct5c01167_si_001.pdf]

Supporting Information: Correlation-driven spin-component-scaled second-order Møller-Plesset perturbation theory (CD-SCS-MP2)

Paulau A.,[†] Soriano-Agueda. L.,[*,†,‡] and Matito E.[*,†,¶]

†*Donostia International Physics Center (DIPC), 20018 Donostia, Euskadi, Spain*

‡*Departamento de Física y Química Teórica, Facultad de Química, Universidad Nacional Autónoma de México, Cd. Universitaria, 04510 Ciudad de México, México*

¶*Ikerbasque Foundation for Science, Plaza Euskadi 5, 48009 Bilbao, Euskadi, Spain*

E-mail: lsorianoagueda@gmail.com; ematito@dipc.org

Contents

1 Guide to obtain optimal c_{OS} and c_{SS} **S-3**

2 Heat maps for diet-GMTKN55 dataset **S-5**

3 GMTKN55 dataset description **S-15**

4 SCS-MP2 *vs.* SCS-MP2* **S-17**

4.1 MAD, RMSD, MAX and WTMAD2 on the diet-GMTKN55 subsets. S-19

4.2 MAD, RMSD, MAX and WTMAD on GMTKN55 subsets. S-23

5 CD-SCS-MP2 *vs.* DFT **S-27**

5.1 Mean Absolute Deviation Plots S-27

5.2 Root-Mean-Square Deviation Plots S-35

5.3 Maximum Mean Absolute Deviation Plots S-40

5.4 Weighted total mean absolute deviation of type 2 Plots S-45

References **S-50**

1 Guide to obtain optimal c_{OS} and c_{SS}

There are two components in the electron correlation energy, c_{OS} and c_{SS} corresponding to the correlation of opposite-spin and same-spin respectively. To get both amounts in the ORCA software from an MP2 calculation, the following input must be used:

```
! MP2 def2-qzvp
! TightSCF
%pal
 nprocs 10
 end
%MaxCore 4000
%scf
  maxiter=100
  end
%mp2
   NatOrbs true
   PrintLevel 3
end

* xyz Charge Multiplicity
Geometry
*
```

Figure S1: Input example in ORCA software to get c_{OS} and c_{SS} coefficients.

In the geometry section, one should include the geometry in Cartesian coordinates. Charge and multiplicity are integers that depend on each of the molecules. PrintLevel 3 is the keyword needed to report c_{OS} and c_{SS} in the output file.

On the other hand, to obtain I_{ND}/I_T and I_{D}/I_T we use the program available at `https://github.com/lasa1988/DC-SCS-MP2/tree/main`.

To compile it you must write the following line in the terminal:

```
gcc program.c  -o  program.x -lm
```

Later you will use the executable as follows:

```
./program.x file.out
```

where file.out is the ORCA output file. At the end of the described procedure you will have $I_{\mathrm{ND}}/I_{\mathrm{T}}$ and $I_{\mathrm{D}}/I_{\mathrm{T}}$.

MP2 correlation energy can be expressed as

$$E_c^{\mathrm{MP2}} = c_{OS}E_c^{OS} + c_{SS}E_c^{SS}, \tag{1}$$

where E_c^{OS} and E_c^{SS} correspond to the opposite- and same-spin correlation components. CD-SCS-MP2 parameterizes c_{OS} and c_{SS} using electron correlation measures[S1–S6] in the following way:

$$c_{SS} = a\frac{\overline{I_{\mathrm{ND}}}}{\overline{I_{\mathrm{T}}}} + b; \qquad c_{OS} = a'\frac{\overline{I_{\mathrm{D}}}}{\overline{I_{\mathrm{T}}}} + b', \tag{2}$$

where for the two-parameter correlation-driven SCS-MP2 method, a'=2.89 and a=1.38, whereas CD4-SCS-MP2 employs the values $a = 0.00$, $a' = 0.42$, $b' = 0.79$, and $b = 0.47$. The optimal coefficients of SCS-MP2* are c_{OS}=1.13 and c_{SS}=0.44, fairly close to the values of SCS-MP2 proposed by Grimme *et al.* (c_{OS}=1.2 and c_{SS}=0.33).[S7] These coefficients have been obtained through a direct minimization of the overall MAD of the diet-GMTKN55 dataset.[S8]

2 Heat maps for diet-GMTKN55 dataset

Heat maps corresponding to the 150 systems of the diet-GMTKN55 dataset.[S8] In all cases, the following notation is used: ACONF/11, where ACONF is one of the GMTKN55 subsets, and 11 represents one of the systems in ACONF. In all cases, there is a range of values (c_{OS} and c_{SS}) for which the absolute deviation (AD) is less than 1 kcal/mol.

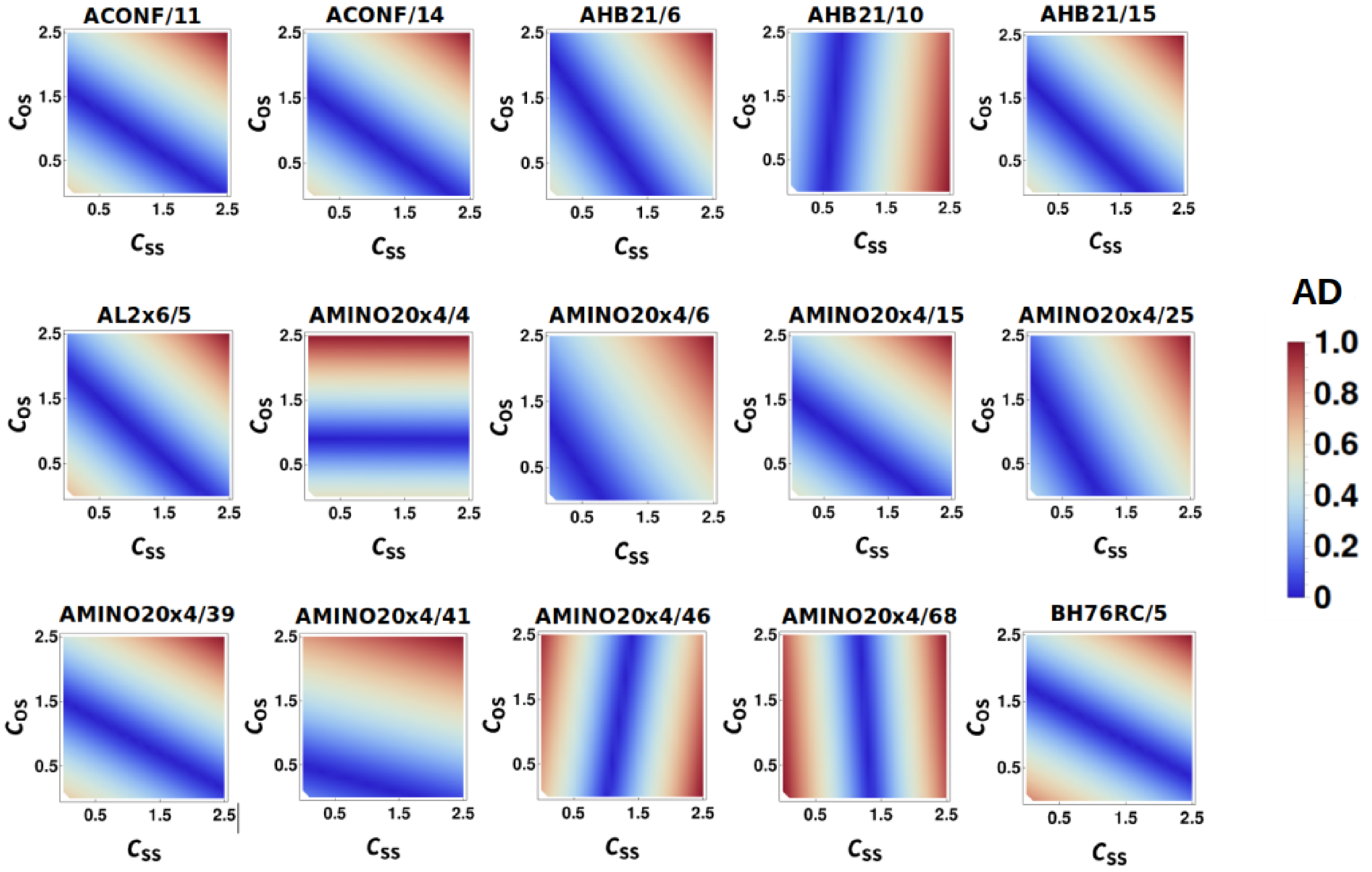

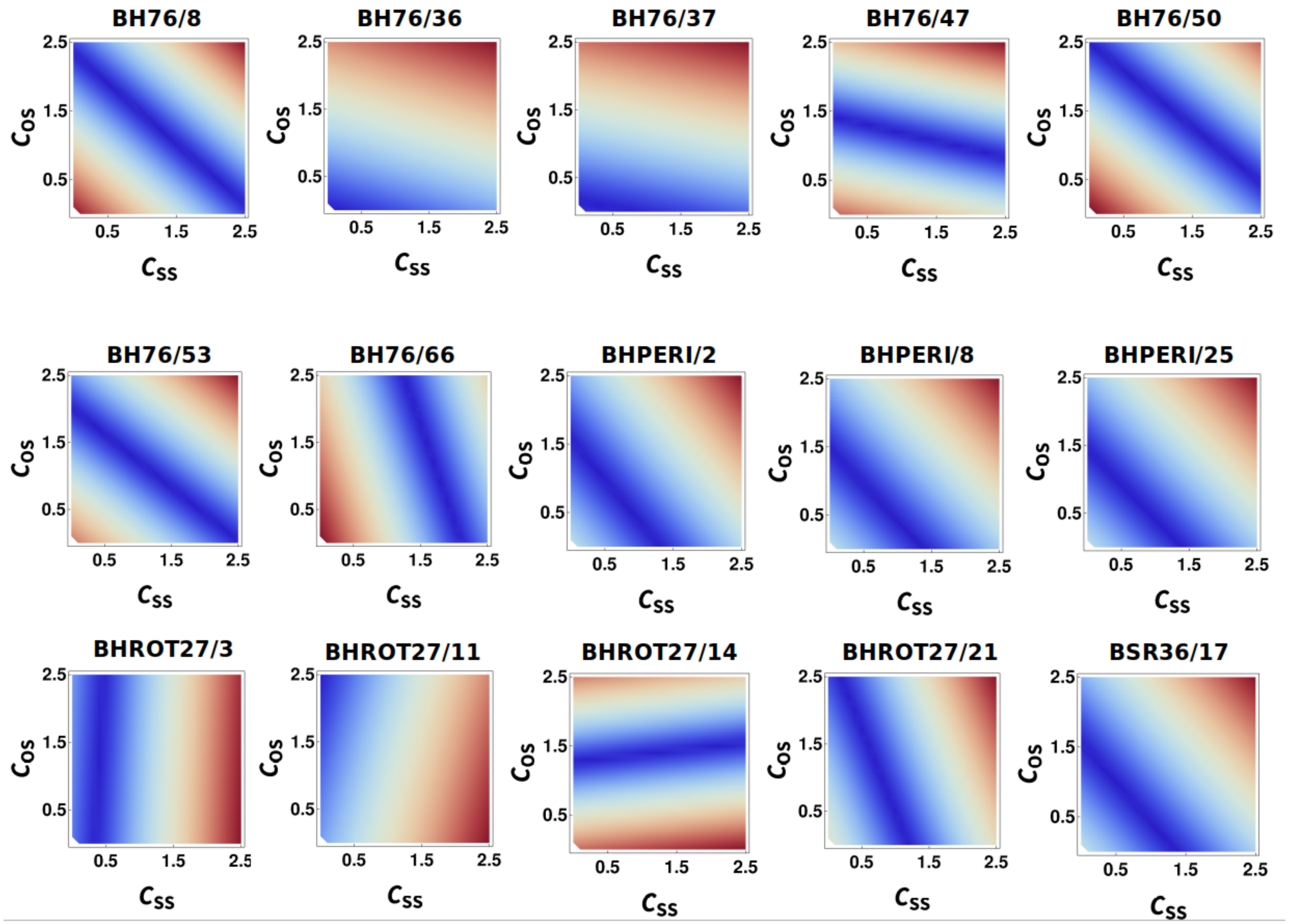
BH76/8
BH76/36
BH76/37
BH76/47
BH76/50
BH76/53
BH76/66
BHPERI/2
BHPERI/8
BHPERI/25
BHROT27/3
BHROT27/11
BHROT27/14
BHROT27/21
BSR36/17
c_{OS}
c_{SS}
0.5
1.5
2.5

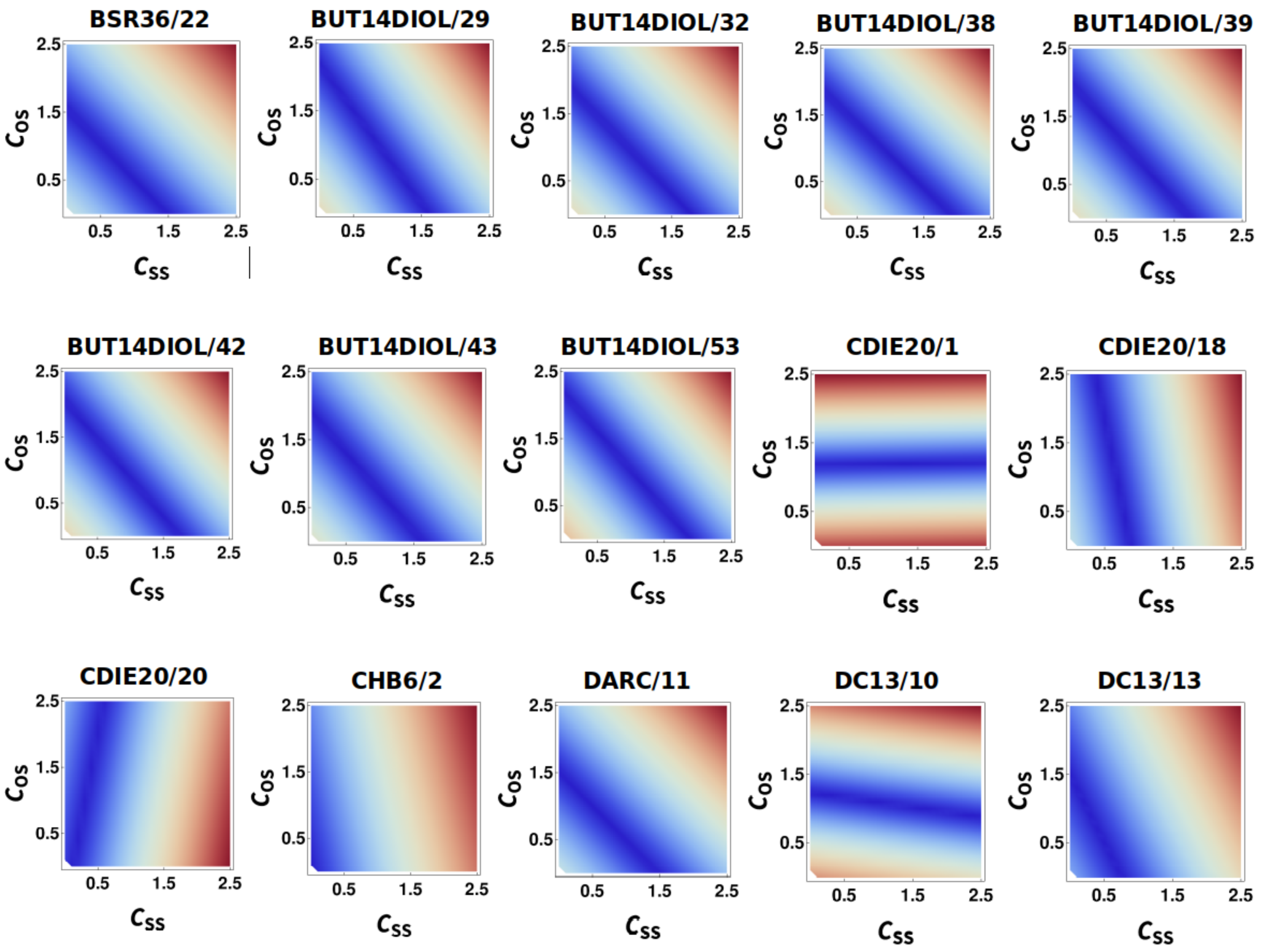
BSR36/22
BUT14DIOL/29
BUT14DIOL/32
BUT14DIOL/38
BUT14DIOL/39
BUT14DIOL/42
BUT14DIOL/43
BUT14DIOL/53
CDIE20/1
CDIE20/18
CDIE20/20
CHB6/2
DARC/11
DC13/10
DC13/13
c_{OS}
c_{SS}
0.5
1.5
2.5

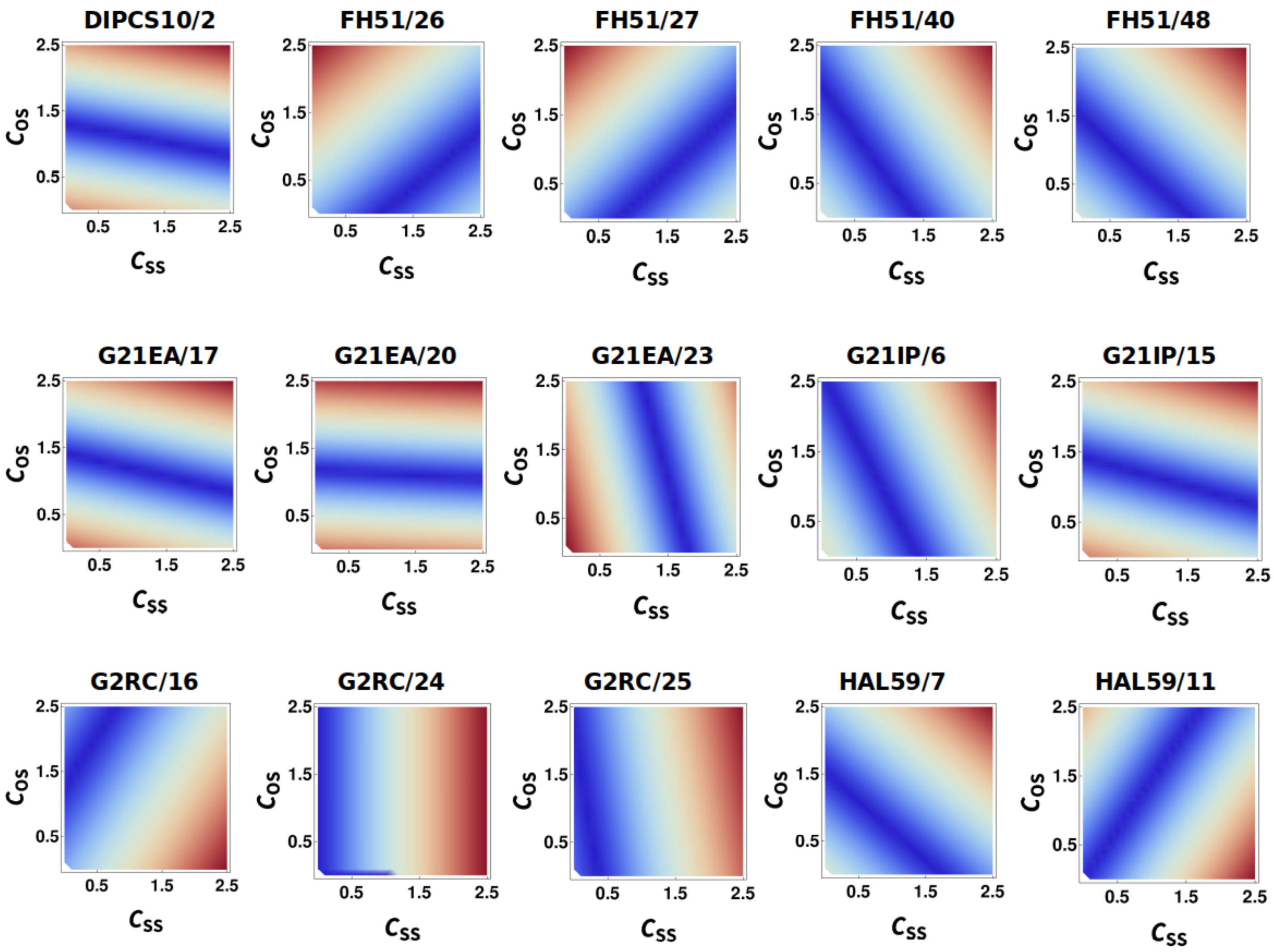
DIPCS10/2
FH51/26
FH51/27
FH51/40
FH51/48
G21EA/17
G21EA/20
G21EA/23
G21IP/6
G21IP/15
G2RC/16
G2RC/24
G2RC/25
HAL59/7
HAL59/11
c_{os}
c_{ss}
0.5
1.5
2.5

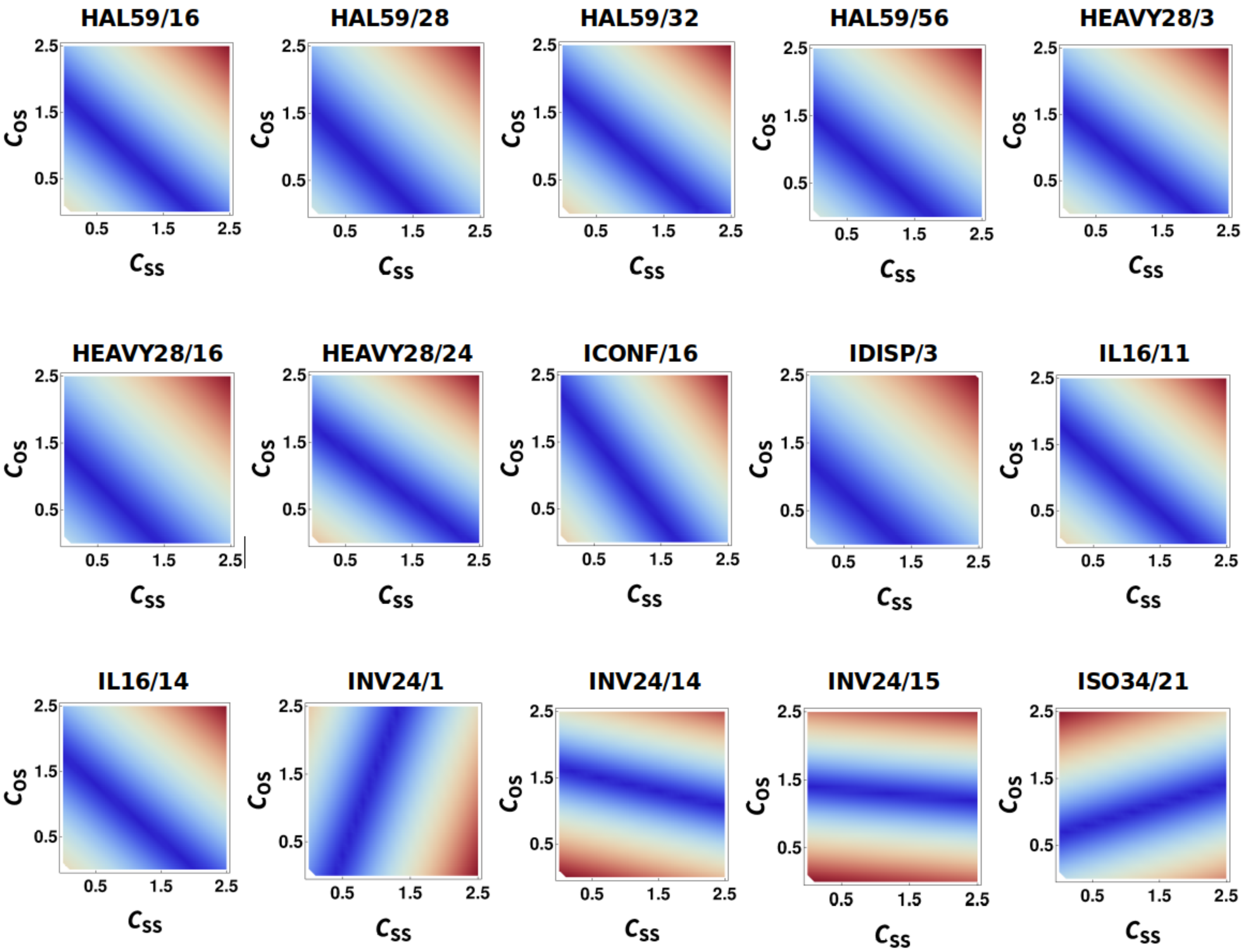
HAL59/16
HAL59/28
HAL59/32
HAL59/56
HEAVY28/3
HEAVY28/16
HEAVY28/24
ICONF/16
IDISP/3
IL16/11
IL16/14
INV24/1
INV24/14
INV24/15
ISO34/21
C_{os}
C_{ss}
0.5
1.5
2.5

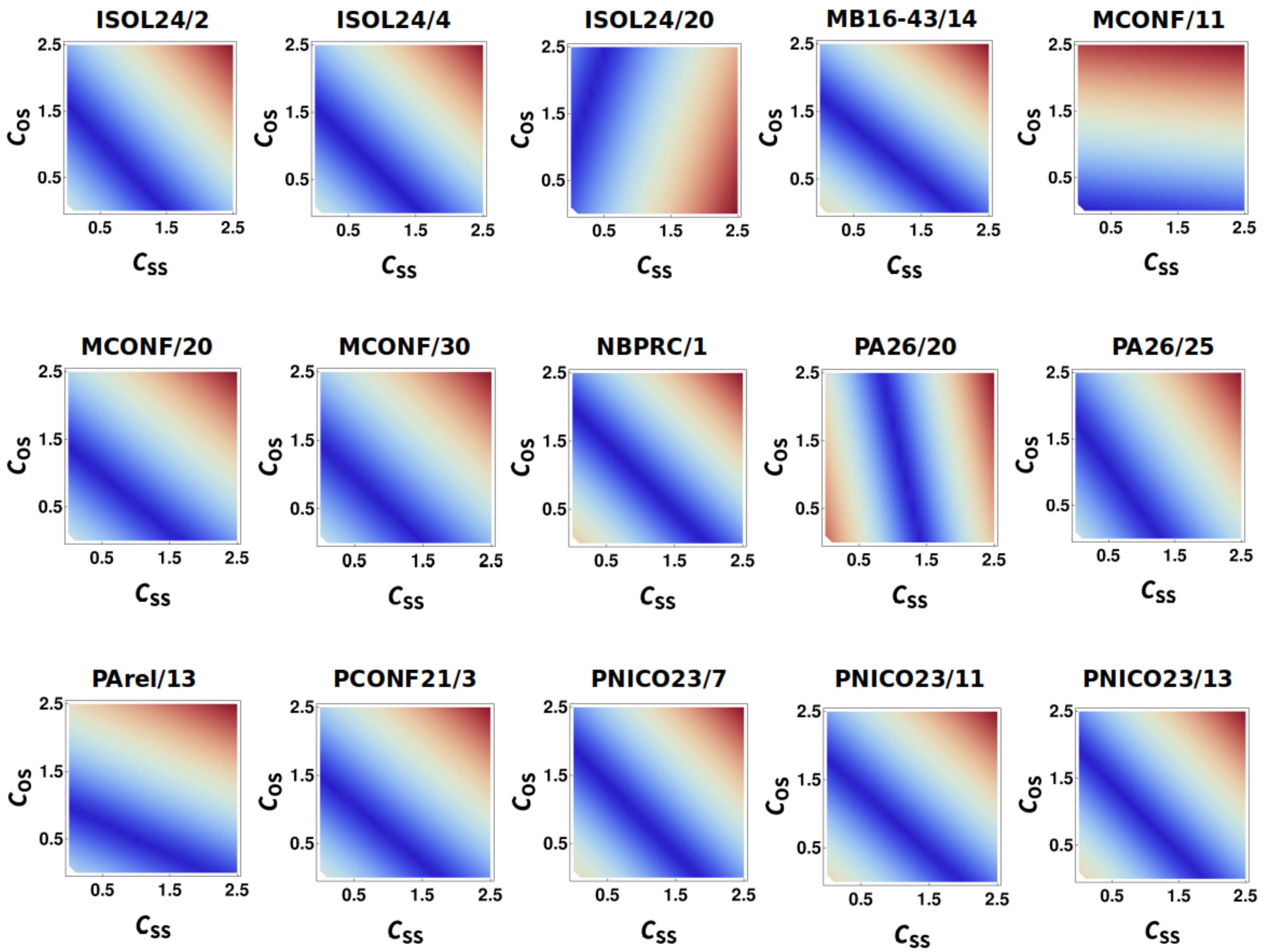
ISOL24/2
ISOL24/4
ISOL24/20
MB16-43/14
MCONF/11
MCONF/20
MCONF/30
NBPRC/1
PA26/20
PA26/25
PArel/13
PCONF21/3
PNICO23/7
PNICO23/11
PNICO23/13
c_{OS}
c_{SS}
0.5
1.5
2.5

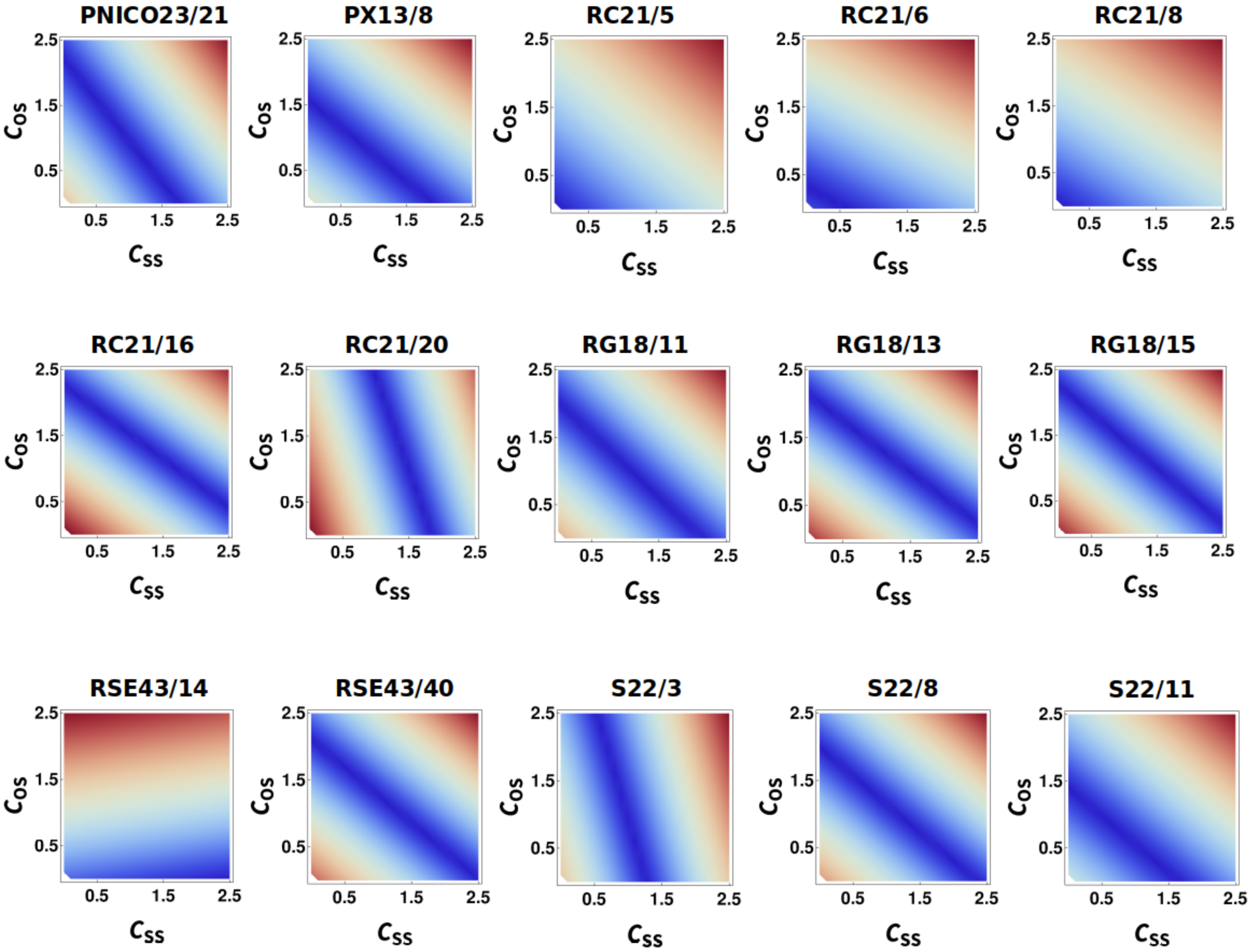
PNICO23/21
PX13/8
RC21/5
RC21/6
RC21/8
RC21/16
RC21/20
RG18/11
RG18/13
RG18/15
RSE43/14
RSE43/40
S22/3
S22/8
S22/11
C_{OS}
C_{SS}
0.5
1.5
2.5

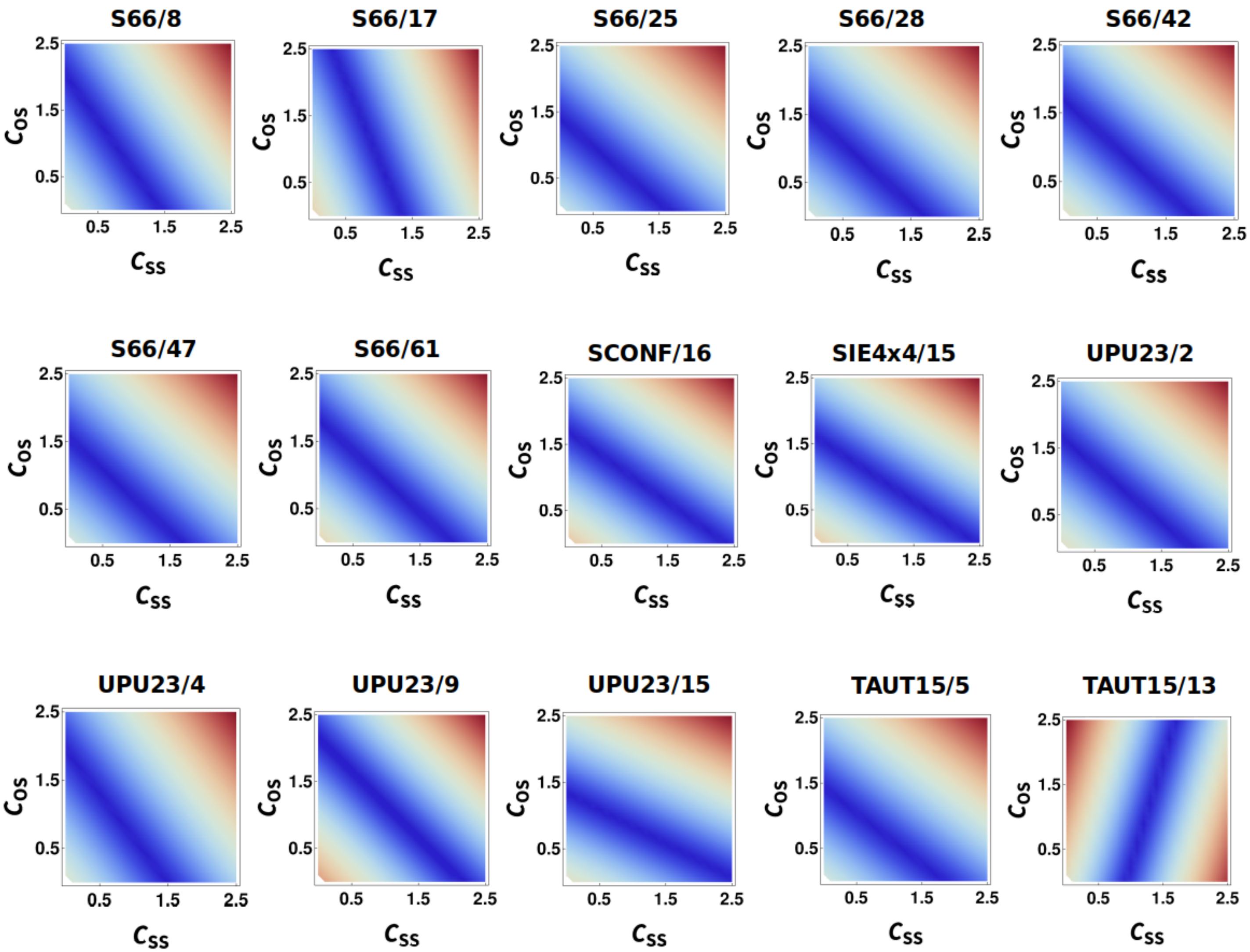
S66/8
S66/17
S66/25
S66/28
S66/42
S66/47
S66/61
SCONF/16
SIE4x4/15
UPU23/2
UPU23/4
UPU23/9
UPU23/15
TAUT15/5
TAUT15/13
c_{OS}
c_{SS}
0.5
1.5
2.5

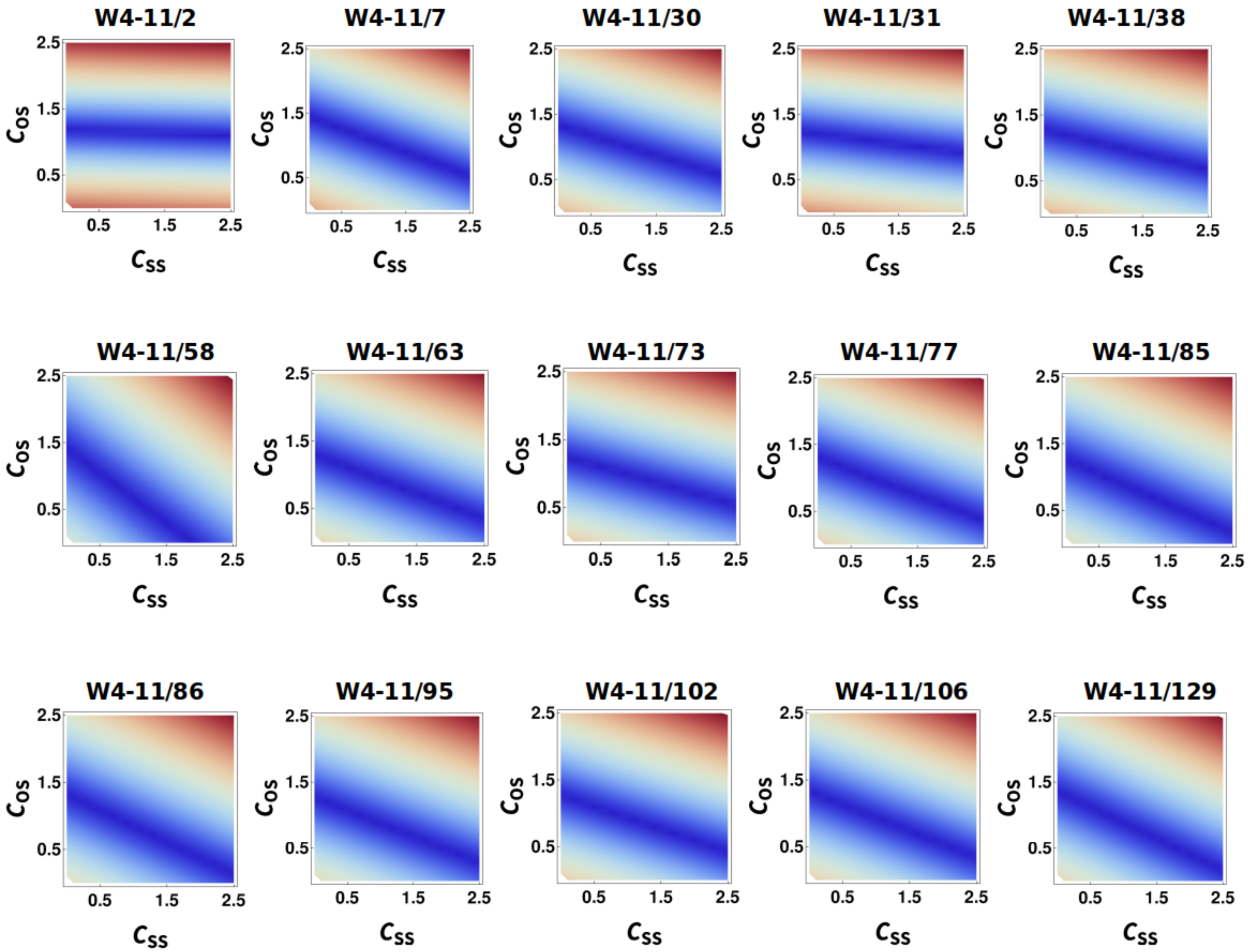
W4-11/2
W4-11/7
W4-11/30
W4-11/31
W4-11/38
W4-11/58
W4-11/63
W4-11/73
W4-11/77
W4-11/85
W4-11/86
W4-11/95
W4-11/102
W4-11/106
W4-11/129
c_{OS}
c_{SS}
0.5
1.5
2.5

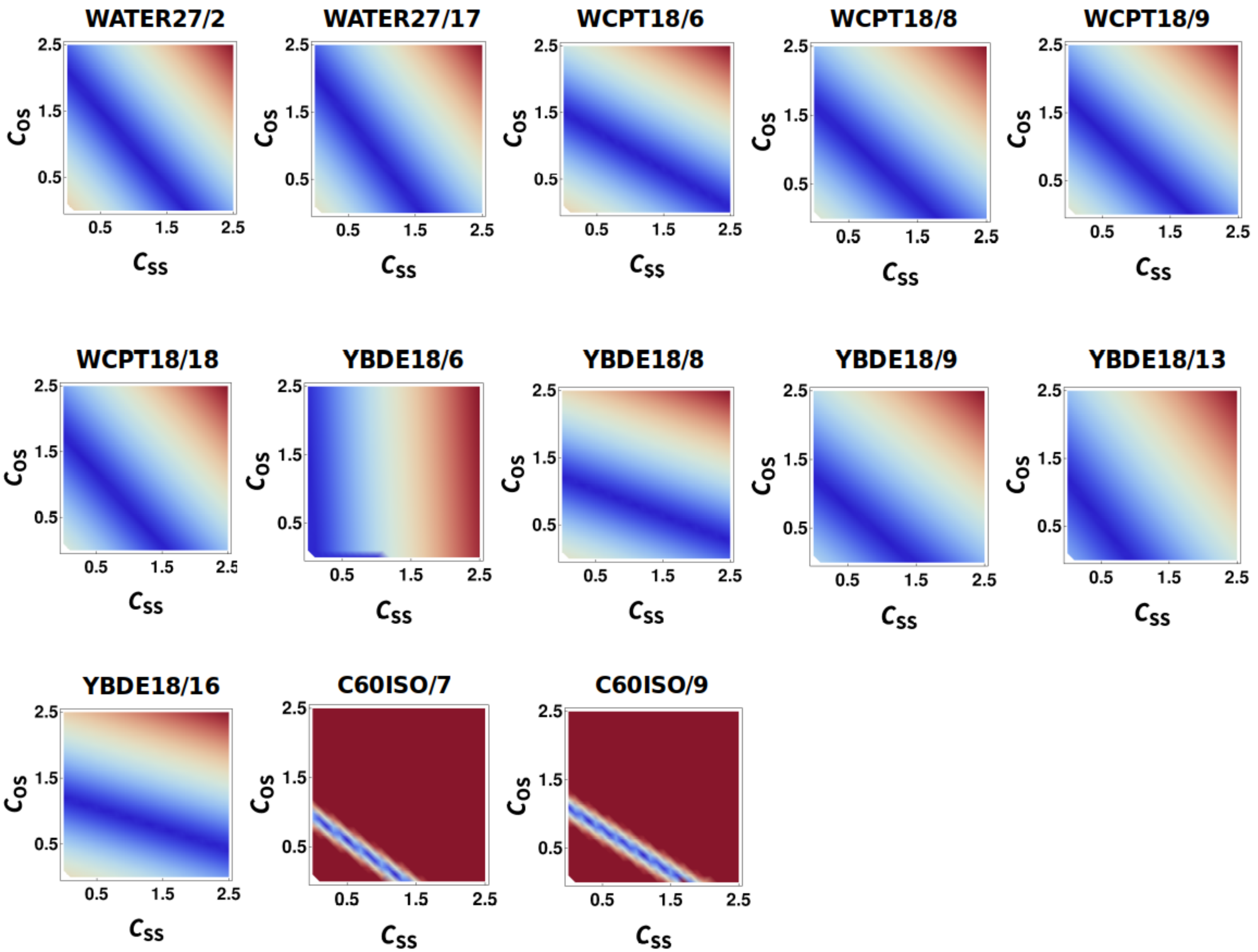

Figure S2: Heat maps for diet-GMTKN55 systems.[S8] Color gradient: in blue the c_{OS} and c_{SS} values are shown where the MAD is less than 1 kcal/mol. As it turns red, the MAD values increase.

3 GMTKN55 dataset description

Table S1: Description of the GMTKN55 dataset. ^{a} W4-11. ^{b} Experimental. ^{c} W1-F12. ^{d} W2-F12. ^{e} CCSD(T)/CBS. ^{f} CCSD(T)-F12. ^{g} W3.2. ^{h} DLPNO-CCSD(T)/CBS. ^{i}Estimated CCSD(T)/CBS. ^{j} DLPNO-CCSD(T)/CBS*. ^{k} W2.2. ^{l} CP-corrected. ^{m} Estimated CCSD(T)-F12/CBS ^{n} W1h-val.

**Subset**	**Description**	**Ref. method**
	Basic properties and reaction energies for small systems	
W4-11[S9]	Total atomisation energies	a
G21EA[S10,S11]	Adiabatic electron affinities	b
G21IP[S10,S11]	Adiabatic ionisation potentials	b
DIPCS10[S12]	Double-ionisation potentials of closed-shell systems	c
PA26[S12]	Adiabatic proton affinities	c,d,e
SIE4x4[S13]	Self-interaction-error related problems	d
ALKBDE10[S14]	Dissociation energies in group-1 and -2 diatomics	b
YBDE18[S15]	Bond-dissociation energies in ylides	d
AL2x6[S12]	Dimerisation energies of AlX_3 compounds	c,d
HEAVYSB11[S12]	Dissociation energies in heavy-element compounds	e
NBPRC[S10,S16,S17]	Oligomerisations and H_2 fragmentations of NH_3/BH_3 systems	c,d
ALK8[S12]	Dissociation and other reactions of alkaline compounds	e
RC21[S12]	Fragmentations and rearrangements in radical cations	c
G2RC[S18,S19]	Reaction energies of selected G2/97 systems	d
BH76RC[S10]	Reaction energies of the BH76 set	d
FH51[S20,S21]	Reaction energies in various (in-)organic systems	f
TAUT15[S12]	Relative energies in tautomers	c
DC13[S10,S22–S32]	13 difficult cases for DFT methods	c,d,e,g,h,i
	Reaction energies for large systems and isomerisation reactions	
MB16-43[S12]	Decomposition energies of artificial molecules	c
DARC[S10,S33]	Reaction energies of Diels–Alder reactions	c
RSE43[S34]	Radical-stabilisation energies	c
BSR36[S35,S36]	Bond-separation reactions of saturated hydrocarbons	h
CDIE20[S37]	Double-bond isomerisation energies in cyclic systems	c
ISO34[S38]	Isomerisation energies of small and medium-sized organic molecules	c
ISOL24[S39]	Isomerisation energies of large of large organic molecules	h

C60ISO[S40]	Relative energies between C_{60} isomers	j
PArel[S12]	Relative energies in protonated isomers	e
	Reaction barrier heights	
BH76[S10,S41,S42]	Barrier heights of hydrogen transfer, heavy atom transfer, nucleophilic substitution, unimolecular and association reactions	d
BHPERI[S12,S43–S45]	Barrier heights of pericyclic reactions	c,d
BHDIV10[S12]	Diverse reaction barrier heights	c,d
INV24[S46]	Inversion/racemisation barrier heights	c,d,h
BHROT27[S12]	Barrier heights for rotation around single bonds	c,d
PX13[S47]	Proton-exchange barriers in H_2O, NH_3, and HF clusters	c
WCPT18[S48]	Proton-transfer barriers in uncatalysed and water-catalysed reactions	k
	Intermolecular noncovalent interactions	
RG18[S12]	Interaction energies in rare-gas complexes	l
ADIM6[S49]	Interaction energies of n-alkane dimers	c
S22[S50]	Binding energies of noncovalently bound dimers	i
S66[S51]	Binding energies of noncovalently bound dimers	i
HEAVY28[S42]	Noncovalent interaction energies between heavy element hydrides	e
WATER27[S52]	Binding energies in $(H_2O)_n$, $H^+(H_2O)_n$ and $OH^-(H_2O)_n$	m
CARBHB12[S12]	Hydrogen-bonded complexes between carbene analogues and H_2O, NH_3 or HCl	d
PNICO23[S53]	Interaction energies in pnicogen-containing dimers	c,d
HAL59[S54,S55]	Binding energies in halogenated dimers (incl. halogen bonds)	i
AHB21[S56]	Interaction energies in anion–neutral dimers	e,f
CHB6[S56]	Interaction energies in cation–neutral dimers	e,i
IL16[S56]	Interaction energies in anion–cation dimers	i
	Intramolecular noncovalent interactions	
IDISP[S10,S16,S38,S57,S58]	Intramolecular dispersion interactions	h
ICONF[S12]	Relative energies in conformers of inorganic systems	
ACONF[S18]	Relative energies of alkane conformers	n
AMINO20x4[S59]	Relative energies in amino acid conformers	m
PCONF21[S12]	Relative energies in tri- and tetrapeptide conformers	h
MCONF[S60]	Relative energies in melatonin conformers	h
SCONF[S10,S61]	Relative energies of sugar conformers	h
UPU23[S62]	Relative energies between RNA-backbone conformers	j
BUT14DIOL[S63]	Relative energies in butane-1,4-diol conformers	c

4 SCS-MP2 *vs.* SCS-MP2*

The MAD, MAX, RMSD, and WTMAD2 for the training set and the GMTKN55 dataset are reported in Table S2. The SCS-MP2* method keeps the deviations calculated practically constant with respect to the version proposed by Grimme (SCS-MP2).

Table S2: Errors with SCS-MP2 and SCS-MP2*. Diet-GMTKN55 dataset: 337 molecular systems corresponding to 150 datapoints. GMTKN55 dataset: 2561 molecules corresponding to 1505 datapoints. SCS-MP2: $c_{OS} = 1.2$ $c_{SS} = 0.33$. SCS-MP2*: $c_{OS} = 1.13$ $c_{SS} = 0.44$. All values in kcal/mol.

	Diet-GMTKN55	
**Error type**	**SCS-MP2**	**SCS-MP2***
**MAD**	2.14	1.98
**MAX**	27.29	25.15
**RMSD**	4.32	4.07
**WTMAD2**	5.45	4.90
	GMTKN55	
**MAD**	2.15	2.07
**MAX**	48.19	44.03
**RMSD**	4.66	4.29
**WTMAD2**	5.90	5.59

Table S3: **Set1:** Basic properties and reaction energies for small systems (475 data points). **Set2:** Reaction energies for large systems and isomerisation reactions (243 data points). **Set3:** Barrier heights (194 data points). **Set4:** Intermolecular noncovalent interactions (304 data points). **Set 5:** Intramolecular noncovalent interactions (291 data points). All values in kcal/mol.

Error type	SCS-MP2	SCS-MP2*
	Set 1	
**MAD**	2.79	2.86
**MAX**	16.44	14.88
**RMSD**	3.87	3.87
**WTMAD 2**	2.82	2.76
	Set 2	
**MAD**	4.09	3.73
**MAX**	48·19	44.03
**RMSD**	9.14	8.17
**WTMAD2**	5.31	5.27
	Set 3	
**MAD**	2.30	2.30
**MAX**	13.78	13.52
**RMSD**	3.47	3.44
**WTMAD2**	6.48	6.51
	Set 4	
**MAD**	1.27	1.08
**MAX**	20.98	19.01
**RMSD**	3.10	2.61
**WTMAD2**	11.46	10.23
	Set 5	
**MAD**	0.31	0.30
**MAX**	4.47	4.87
**RMSD**	0.51	0.51
**WTMAD2**	5.23	5.00

4.1 MAD, RMSD, MAX and WTMAD2 on the diet-GMTKN55 subsets.

Table S4: MAD for the 49 subsets of diet-GMTKN55 dataset.[S8] All values in kcal/mol.

Subset	MP2	SCS-MP2	SCS-MP2*	CD2-SCS-MP2	CD4-SCS-MP2
Amino20x4	0.15	0.15	0.14	0.13	0.13
CDIE20	0.39	0.08	0.11	5.33	4.30
FH51	1.46	2.19	1.94	1.26	1.27
TAUT15	0.20	0.05	0.05	0.05	0.04
ISOL24	6.65	0.53	0.15	0.69	0.91
PCONF21	1.76	0.01	0.10	0.02	0.06
DARC	5.43	0.29	0.72	1.96	2.13
ICONF	0.04	0.40	0.36	0.40	0.41
ISO34	0.00	0.03	0.03	0.03	0.03
BHROT27	0.13	0.09	0.09	0.11	0.12
UPU23	0.45	0.57	0.51	0.54	0.57
RG18	0.05	0.13	0.12	0.41	0.59
IDISP	3.32	0.00	0.20	0.52	0.04
DC13	11.25	2.69	3.91	5.28	3.95
BH76	4.15	5.94	5.77	1.29	1.28
HEAVY28	0.29	0.18	0.18	0.19	0.22
INV24	0.61	0.53	0.57	0.72	0.74
PX13	4.37	1.65	1.33	2.37	2.07
G2RC	5.99	2.81	2.81	1.74	3.00
CHB6	0.86	0.69	0.72	1.06	0.69
RC21	6.10	6.84	6.69	1.09	1.23
BHPERI	9.12	1.17	1.86	0.55	0.82
NBPRC	0.16	2.57	2.35	3.02	2.04
G21EA	3.08	2.92	2.99	0.92	1.55
AL2X6	0.26	3.22	3.03	3.52	3.03
C60ISO	30.89	19.25	19.31	17.49	11.34
SCONF	0.08	0.93	0.91	1.08	1.07
DIPCS10	1.75	0.58	1.36	1.73	0.50
S22	0.89	0.76	0.71	0.54	0.51
PA26	1.53	2.20	1.83	0.62	1.50
W4-11	8.04	2.73	1.99	1.55	1.68
BH76RC	1.34	2.62	2.70	1.47	1.91
S66	1.03	0.60	0.52	0.59	0.33
MB16-43	14.47	27.29	25.15	5.55	1.04
G21IP	3.95	6.39	5.98	3.25	2.60
HAL59	0.63	1.02	0.30	0.50	0.37
BUT14DIOL	0.10	0.32	0.28	0.31	0.27
PArel	2.11	1.64	1.55	1.04	0.98
AHB21	0.50	0.62	0.48	0.50	0.51
PNICO23	0.18	0.58	0.51	0.64	0.56
ACONF	0.12	0.23	0.23	0.30	0.25
WCPT18	2.47	1.54	1.32	1.72	1.66
IL16	0.59	1.53	1.40	2.83	0.18
BSR36	2.90	0.04	0.29	0.13	0.11
RSE43	1.41	2.11	2.07	0.97	0.80
MCONF	1.07	0.33	0.37	0.34	0.33
WATER27	0.55	1.57	1.36	1.26	1.09
SIE4x4	0.62	2.98	2.97	2.41	2.10
YBDE18	6.07	3.32	2.74	1.69	1.41
TOTAL	3.17	2.14	1.98	1.36	1.18

Table S5: MAX for the 49 subsets of diet-GMTKN55 dataset.[S8] All values in kcal/mol.

**Subset**	**MP2**	**SCS-MP2**	**SCS-MP2***	**CD2-SCS-MP2**	**CD4-SCS-MP2**
Amino20x4	0.36	0.50	0.44	0.45	0.43
CDIE20	0.64	0.22	0.15	15.40	12.38
FH51	2.30	4.04	3.64	2.48	2.44
TAUT15	0.39	0.06	0.06	0.06	0.06
ISOL24	13.72	0.99	0.23	1.11	1.34
PCONF21	1.76	0.01	0.10	0.02	0.06
DARC	5.43	0.29	0.72	1.96	2.13
ICONF	0.04	0.40	0.36	0.40	0.41
ISO34	0.00	0.03	0.03	0.03	0.03
BHROT27	0.15	0.25	0.20	0.23	0.25
UPU23	0.95	0.95	0.85	0.93	1.00
RG18	0.07	0.16	0.15	0.74	1.10
IDISP	3.32	0.00	0.20	0.52	0.04
DC13	21.13	5.19	7.20	9.11	6.83
BH76	8.11	9.23	8.67	3.78	3.79
HEAVY28	0.56	0.23	0.22	0.27	0.35
INV24	0.95	0.59	0.71	0.99	1.05
PX13	4.37	1.65	1.33	2.37	2.07
G2RC	8.66	5.11	3.53	2.39	4.84
CHB6	0.86	0.69	0.72	1.06	0.69
RC21	11.48	9.91	10.00	2.19	2.63
BHPERI	10.92	1.90	2.63	0.69	1.24
NBPRC	0.16	2.57	2.35	3.02	2.04
G21EA	3.55	5.15	4.76	1.53	2.29
AL2X6	0.26	3.22	3.03	3.52	3.03
C60ISO	37.74	24.27	24.52	22.19	14.95
SCONF	0.08	0.93	0.91	1.08	1.07
DIPCS10	1.75	0.58	1.36	1.73	0.50
S22	2.55	1.56	1.33	0.96	0.93
PA26	2.16	4.02	3.57	0.81	1.99
W4-11	19.39	8.96	6.32	3.22	3.76
BH76RC	1.34	2.62	2.70	1.47	1.91
S66	2.31	1.61	1.37	1.31	1.16
MB16-43	14.47	27.29	25.15	5.55	1.04
G21IP	7.36	12.11	10.86	6.29	3.90
HAL59	1.39	5.23	0.91	1.13	1.03
BUT14DIOL	0.21	0.47	0.42	0.47	0.42
PArel	2.11	1.64	1.55	1.04	0.98
AHB21	0.80	0.73	0.56	0.69	0.72
PNICO23	0.42	0.97	0.85	1.10	0.96
ACONF	0.17	0.28	0.27	0.36	0.29
WCPT18	4.00	1.77	1.83	2.69	2.44
IL16	0.65	1.78	1.65	3.15	0.33
BSR36	3.47	0.08	0.29	0.26	0.20
RSE43	1.76	2.40	2.33	1.27	0.83
MCONF	1.42	0.48	0.47	0.43	0.43
WATER27	0.91	1.72	1.45	1.30	1.12
SIE4x4	0.62	2.98	2.97	2.41	2.10
YBDE18	9.54	5.86	4.44	2.46	2.95
TOTAL	37.74	27.29	25.15	22.19	14.95

Table S6: RMSD for the 49 subsets of diet-GMTKN55 dataset.[S8] All values in kcal/mol.

**Subset**	**MP2**	**SCS-MP2**	**SCS-MP2***	**CD2-SCS-MP2**	**CD4-SCS-MP2**
Amino20x4	0.20	0.21	0.19	0.19	0.18
CDIE20	0.43	0.13	0.11	8.89	7.15
FH51	1.64	2.86	2.58	1.67	1.71
TAUT15	0.27	0.05	0.05	0.05	0.04
ISOL24	8.57	0.63	0.16	0.79	1.04
PCONF21	1.76	0.01	0.10	0.02	0.06
DARC	5.43	0.29	0.72	1.96	2.13
ICONF	0.04	0.40	0.36	0.40	0.41
ISO34	0.00	0.03	0.03	0.03	0.03
BHROT27	0.13	0.13	0.11	0.14	0.15
UPU23	0.56	0.69	0.62	0.66	0.69
RG18	0.05	0.13	0.13	0.47	0.69
IDISP	3.32	0.00	0.20	0.52	0.04
DC13	14.98	3.67	5.11	6.52	4.89
BH76	4.66	6.48	6.26	1.70	1.73
HEAVY28	0.37	0.18	0.18	0.21	0.24
INV24	0.68	0.53	0.60	0.77	0.81
PX13	4.37	1.65	1.33	2.37	2.07
G2RC	6.28	3.25	2.88	1.82	3.30
CHB6	0.86	0.69	0.72	1.06	0.69
RC21	6.78	7.25	7.08	1.37	1.60
BHPERI	9.33	1.36	2.01	0.58	0.90
NBPRC	0.16	2.57	2.35	3.02	2.04
G21EA	3.12	3.39	3.44	1.03	1.68
AL2X6	0.26	3.22	3.03	3.52	3.03
C60ISO	31.64	19.89	20.00	18.11	11.90
SCONF	0.08	0.93	0.91	1.08	1.07
DIPCS10	1.75	0.58	1.36	1.73	0.50
S22	1.47	0.95	0.85	0.62	0.59
PA26	1.65	2.85	2.52	0.65	1.58
W4-11	9.81	3.69	2.45	1.82	2.03
BH76RC	1.34	2.62	2.70	1.47	1.91
S66	1.32	0.78	0.68	0.77	0.49
MB16-43	14.47	27.29	25.15	5.55	1.04
G21IP	4.66	7.60	6.91	3.90	2.76
HAL59	0.77	2.14	0.42	0.59	0.55
BUT14DIOL	0.12	0.35	0.31	0.34	0.30
PArel	2.11	1.64	1.55	1.04	0.98
AHB21	0.54	0.63	0.49	0.56	0.54
PNICO23	0.23	0.62	0.55	0.71	0.61
ACONF	0.13	0.24	0.23	0.31	0.25
WCPT18	2.68	1.56	1.36	2.01	1.86
IL16	0.60	1.55	1.43	2.85	0.23
BSR36	2.96	0.06	0.29	0.18	0.14
RSE43	1.45	2.13	2.08	1.01	0.80
MCONF	1.15	0.35	0.38	0.35	0.34
WATER27	0.65	1.58	1.36	1.26	1.09
SIE4x4	0.62	2.98	2.97	2.41	2.10
YBDE18	6.65	3.67	3.18	1.87	1.78
TOTAL	6.10	4.32	4.07	2.92	2.23

Table S7: WTMAD2 for the 49 subsets of diet-GMTKN55 dataset. All values in kcal/mol.

**Subset**	**MP2**	**SCS-MP2**	**SCS-MP2***	**CD2-SCS-MP2**	**CD4-SCS-MP2**
Amino20x4	28.32	27.77	25.23	24.70	24.33
CDIE20	16.57	3.55	4.68	223.99	180.77
FH51	10.69	16.03	14.20	9.23	9.28
TAUT15	7.52	1.79	1.90	1.78	1.45
ISOL24	51.72	4.12	1.14	5.33	7.05
PCONF21	61.55	0.21	3.61	0.84	2.13
DARC	9.50	0.52	1.25	3.43	3.73
ICONF	0.78	7.01	6.23	6.95	7.08
ISO34	0.01	0.14	0.10	0.12	0.11
BHROT27	4.62	3.23	3.14	4.07	4.36
UPU23	17.91	22.71	20.08	21.44	22.47
RG18	14.18	37.59	36.18	119.56	172.40
IDISP	13.26	0.01	0.81	2.08	0.15
DC13	23.26	5.55	8.09	10.91	8.16
BH76	88.66	126.93	123.41	27.59	27.47
HEAVY28	39.99	24.28	24.53	26.52	29.83
INV24	3.28	2.84	3.07	3.86	3.94
PX13	7.44	2.81	2.27	4.05	3.53
G2RC	19.92	9.35	9.36	5.78	9.97
CHB6	1.83	1.47	1.52	2.25	1.46
RC21	48.54	54.42	53.24	8.67	9.78
BHPERI	74.46	9.55	15.18	4.46	6.74
NBPRC	0.33	5.27	4.83	6.21	4.18
G21EA	15.63	14.81	15.19	4.64	7.86
AL2X6	0.41	5.10	4.79	5.57	4.80
C60ISO	35.74	22.27	22.34	20.23	13.12
SCONF	1.04	11.53	11.27	13.30	13.16
DIPCS10	0.15	0.05	0.12	0.15	0.04
S22	20.72	17.65	16.69	12.61	11.83
PA26	0.92	1.32	1.10	0.37	0.90
W4-11	22.33	7.57	5.52	4.32	4.66
BH76RC	3.57	6.95	7.19	3.91	5.08
S66	74.73	43.45	37.65	42.58	24.27
MB16-43	1.76	3.31	3.05	0.67	0.13
G21IP	2.62	4.23	3.96	2.15	1.72
HAL59	46.70	76.04	22.51	37.01	27.31
BUT14DIOL	16.83	51.68	45.68	49.73	44.37
PArel	25.90	20.13	19.08	12.71	11.98
AHB21	3.77	4.72	3.65	3.83	3.88
PNICO23	9.49	30.69	27.37	34.31	29.73
ACONF	7.50	14.36	14.01	18.57	15.22
WCPT18	16.08	9.98	8.56	11.21	10.78
IL16	0.62	1.59	1.46	2.95	0.18
BSR36	20.38	0.28	2.02	0.93	0.80
RSE43	21.03	31.57	30.90	14.46	11.99
MCONF	36.57	11.36	12.84	11.72	11.30
WATER27	0.76	2.20	1.91	1.76	1.53
SIE4x4	1.05	5.02	5.01	4.07	3.54
YBDE18	35.01	19.17	15.82	9.73	8.12
TOTAL	6.72	5.45	4.90	5.75	5.52

4.2 MAD, RMSD, MAX and WTMAD on GMTKN55 subsets.

Table S8: MAD for the 55 subsets of GMTKN55 dataset. The data from PBE, B3LYP, M06-2X, ωB97X, and DSD-PBEP86 consider effects dispersion and were taken from Ref. 49. All values in kcal/mol.

Subset	MP2	SCS-MP2	SCS-MP2*	CD2-SCS-MP2	CD4-SCS-MP2	PBE	B3LYP	M06-2X	ωB97X	DSD-PBEP86
W4-11	6.99	3.08	3.30	1.80	2.24	15.68	3.40	3.16	2.86	3.30
G21EA	2.76	3.38	3.61	3.79	3.67	3.43	1.91	1.76	1.53	1.51
G21IP	2.87	3.03	3.10	2.64	2.53	3.85	3.55	2.64	2.87	2.10
DIPCS10	5.00	5.74	6.52	4.92	5.37	4.50	4.73	3.16	5.45	3.91
PA26	1.85	1.28	1.19	1.65	1.45	2.19	2.87	1.23	3.66	0.98
SIE4x4	1.61	2.30	2.43	2.02	2.04	23.72	18.06	8.67	12.35	4.92
ALKBDE10	6.30	2.90	2.98	2.38	2.72	6.30	4.39	4.79	4.43	3.19
YBDE18	6.41	3.15	2.75	2.10	2.31	4.93	4.72	2.40	2.41	1.23
AL2X6	0.80	2.89	2.71	2.98	2.61	1.63	2.71	0.90	3.02	2.52
HEAVYSB11	3.50	2.10	2.05	2.56	2.42	3.55	3.30	8.16	2.37	2.09
NBPRC	1.11	1.57	1.38	1.50	1.14	2.41	2.00	0.95	1.89	1.08
ALK8	3.20	3.36	3.22	4.23	2.91	4.14	2.48	2.31	3.58	1.57
RC21	6.76	7.61	7.40	2.26	2.73	6.85	2.44	1.63	2.98	1.86
G2RC	3.00	2.17	2.09	1.91	1.81	6.92	2.73	1.92	3.88	1.54
FH51	2.58	1.51	1.58	1.59	1.56	3.17	2.61	1.20	1.88	0.85
TAUT15	0.75	0.69	0.56	0.62	0.59	1.84	1.16	0.78	0.74	0.45
DC13	8.31	4.09	3.86	4.11	2.94	8.63	10.57	7.51	6.40	2.94
MB16-43	21.97	16.62	14.55	11.27	7.78	24.26	24.84	15.68	36.50	17.40
DARC	3.68	0.81	0.84	1.07	1.11	3.31	8.03	2.16	1.32	1.25
RSE43	2.09	2.32	2.26	1.15	1.24	2.94	1.72	0.63	1.39	0.86
BSR36	4.54	0.65	0.76	0.85	0.61	3.17	3.35	2.48	4.13	3.55
CDIE20	0.54	0.18	0.20	0.95	0.80	1.65	1.00	0.54	0.63	0.56
ISO34	1.11	0.48	0.53	0.58	0.57	1.49	1.78	1.23	1.03	0.53
ISOL24	2.89	0.89	0.90	1.07	1.12	4.39	5.80	2.74	2.64	1.88
C60ISO	16.35	10.29	10.17	9.36	6.21	10.72	2.22	6.88	13.56	7.86
PArel	0.93	0.55	0.51	0.42	0.40	1.81	1.18	0.97	0.58	0.50
BH76	3.53	4.32	4.28	3.25	3.07	9.62	5.70	2.34	1.88	1.20
BHPERI	7.71	1.27	1.69	0.97	1.02	6.69	1.18	1.35	3.06	1.08
BHDIV10	2.25	1.55	1.48	2.26	1.97	8.87	3.22	1.05	1.00	1.30
INV24	1.06	0.99	0.98	1.33	1.27	2.07	1.05	1.28	1.48	0.64
BHROT27	0.23	0.14	0.12	0.19	0.18	0.47	0.41	0.36	0.39	0.19
PX13	2.61	1.24	1.06	1.92	1.66	12.02	4.33	5.32	1.81	2.27
WCPT18	2.54	1.42	1.17	1.88	1.77	9.34	2.27	1.88	1.41	1.41
RG18	0.16	0.28	0.27	0.39	0.41	0.26	0.13	0.23	0.21	0.23
ADIM6	0.26	1.02	0.94	1.10	0.93	0.21	0.11	0.27	0.34	1.40
S22	1.00	0.64	0.57	0.59	0.50	0.48	0.31	0.34	0.21	0.80
S66	0.60	0.57	0.49	0.65	0.43	0.40	0.26	0.22	0.24	0.74
HEAVY28	0.94	0.62	0.74	0.73	0.72	0.42	0.34	0.33	0.19	0.25
WATER27	1.01	5.53	4.95	3.02	2.71	8.92	4.07	3.70	2.33	1.04
CARBHB12	0.62	0.32	0.25	0.39	0.28	1.91	0.88	0.25	0.57	0.30
PNICO23	0.26	0.69	0.61	0.77	0.61	1.32	0.48	0.29	0.24	0.28
HAL59	2.81	1.61	1.09	1.11	1.07	1.18	0.57	0.35	0.42	0.38
AHB21	0.49	0.67	0.56	1.20	0.96	1.15	0.33	0.95	0.30	0.28
CHB6	0.93	0.59	0.61	1.20	0.57	0.93	1.41	1.42	1.26	0.59
IL16	0.47	1.48	1.36	1.90	0.72	0.59	0.76	0.47	1.06	1.05
IDISP	5.96	1.90	1.91	2.73	1.86	2.76	3.57	2.07	2.68	2.21
ICONF	0.17	0.23	0.19	0.20	0.19	0.32	0.29	0.32	0.42	0.18
ACONF	0.12	0.15	0.14	0.19	0.15	0.07	0.05	0.27	0.07	0.12
Amino20x4	0.23	0.25	0.24	0.24	0.24	0.34	0.21	0.30	0.24	0.18
PCONF21	0.83	0.22	0.24	0.24	0.23	1.25	0.53	1.09	0.31	0.85
MCONF	1.04	0.25	0.29	0.27	0.26	0.49	0.22	0.55	0.30	0.32
SCONF	0.33	0.45	0.40	0.50	0.47	0.80	0.30	0.26	0.27	0.14
UPU23	0.64	0.36	0.33	0.36	0.38	0.53	0.61	0.50	0.77	0.85
BUT14DIOL	0.12	0.32	0.28	0.30	0.27	0.46	0.31	0.13	0.11	0.14
BH76RC	2.95	1.38	1.54	0.86	1.12	4.18	2.25	1.18	1.59	0.84
TOTAL	3.13	2.15	2.07	1.69	1.53					

Table S9: MAX for the 55 subsets of GMTKN55 dataset. The data from PBE, B3LYP, M06-2X, ωB97X, and DSD-PBEP86 consider effects dispersion and were taken from Ref. 49. All values in kcal/mol.

Subset	MP2	SCS-MP2	SCS-MP2*	CD2-SCS-MP2	CD4-SCS-MP2	PBE	B3LYP	M06-2X	ωB97X	DSD-PBEP86
W4-11	23.39	12.67	12.46	9.17	9.30	54.18	9.79	17.49	4.13	2.01
G21EA	6.17	6.96	6.15	8.98	6.97	7.72	7.45	4.15	4.46	4.72
G21IP	11.31	12.11	10.86	6.79	8.18	10.17	9.59	11.55	6.91	6.58
DIPCS10	11.63	9.01	9.77	9.20	9.32	7.11	9.22	6.58	1.98	0.96
PA26	9.26	4.02	3.57	5.19	4.01	7.34	9.06	3.99	7.77	4.25
SIE4x4	5.68	4.10	4.66	3.84	4.37	46.59	40.25	22.39	30.87	11.44
ALKBDE10	14.56	7.44	6.21	7.57	7.95	27.70	7.51	1.77	7.54	2.05
YBDE18	15.52	8.44	8.46	5.89	7.07	11.05	2.64	0.14	0.51	4.15
AL2X6	2.11	3.51	3.26	3.89	3.32	1.51	2.01	3.50	1.93	1.43
HEAVYSB11	5.76	8.40	8.62	9.57	6.79	6.59	0.19	0.51	2.50	0.91
NBPRC	2.43	3.82	3.43	3.64	2.97	3.37	7.97	1.93	6.34	2.90
ALK8	16.31	9.98	9.97	13.41	6.54	12.21	5.58	6.02	0.04	3.88
RC21	14.67	16.44	14.88	9.99	10.05	14.16	4.50	4.95	6.10	1.49
G2RC	8.97	5.11	3.98	4.72	6.15	19.64	7.82	2.90	10.99	4.98
FH51	13.07	8.84	8.82	8.61	8.55	12.78	8.87	3.21	4.10	2.66
TAUT15	1.72	1.97	1.57	1.67	1.62	5.26	2.71	1.93	1.28	0.76
DC13	22.96	9.77	9.02	9.78	8.15	17.53	21.27	15.24	16.49	7.15
MB16-43	46.70	48.19	44.03	18.92	17.85	72.41	19.81	37.90	6.89	4.55
DARC	5.69	2.44	1.92	2.02	2.13	5.22	9.66	3.54	1.80	2.55
RSE43	11.99	10.94	10.50	5.53	6.27	0.79	0.62	0.51	0.61	7.04
BSR36	14.27	3.36	4.32	4.76	2.53	1.12	1.23	0.65	1.36	1.52
CDIE20	1.84	1.03	1.08	15.40	12.38	2.86	2.02	2.09	2.12	1.20
ISO34	5.25	2.63	2.65	2.30	2.39	4.65	10.46	4.68	3.29	2.54
ISOL24	13.72	2.99	2.83	2.69	2.76	16.18	19.03	3.92	6.86	6.68
C60ISO	37.74	24.27	24.52	22.19	14.95	1.67	2.22	10.82	20.82	0.66
PArel	3.87	2.66	2.37	1.33	0.98	6.68	5.40	3.90	2.45	1.81
BH76	13.20	13.78	13.52	6.97	7.66	1.44	16.19	44.30	4.12	9.89
BHPERI	12.86	2.93	3.53	5.47	4.65	3.37	1.87	4.40	6.27	0.67
BHDIV10	7.55	2.88	2.81	3.41	2.79	2.20	5.18	1.30	2.63	1.92
INV24	11.60	6.57	6.28	7.02	7.36	2.33	3.25	7.88	7.12	3.47
BHROT27	0.67	0.57	0.48	0.44	0.45	1.65	1.31	1.17	1.33	0.64
PX13	4.37	2.24	1.97	3.25	2.63	9.15	2.61	1.33	0.30	1.00
WCPT18	4.30	2.31	1.99	2.93	2.57	4.98	1.08	3.58	5.33	0.11
RG18	0.77	0.95	0.94	1.04	1.17	0.81	0.25	0.87	0.03	0.16
ADIM6	0.59	1.59	1.45	1.75	1.47	0.29	0.24	0.82	0.66	0.47
S22	3.95	1.89	1.64	1.66	1.57	1.25	1.16	1.22	0.93	0.06
S66	2.31	1.61	1.37	1.84	1.16	1.47	1.38	0.81	1.10	0.17
HEAVY28	19.44	6.87	10.56	8.35	10.05	1.04	0.63	0.99	0.37	0.04
WATER27	3.93	20.98	19.01	14.54	13.31	30.19	13.89	9.98	7.41	0.98
CARBHB12	1.64	0.72	0.58	0.81	0.64	4.51	2.20	0.88	1.38	1.14
PNICO23	1.12	2.00	1.73	2.47	2.10	6.75	1.33	0.71	0.18	0.21
HAL59	35.81	16.48	14.40	8.79	8.43	6.70	3.22	1.07	0.31	1.04
AHB21	2.89	1.14	1.02	3.75	3.76	1.76	0.58	0.67	0.41	0.54
CHB6	2.36	1.88	1.89	2.05	1.47	1.42	1.30	0.49	2.19	0.22
IL16	1.09	2.38	2.24	3.15	2.15	0.09	1.50	0.85	2.45	1.67
IDISP	12.63	4.47	4.87	6.52	6.00	9.08	12.38	4.82	5.52	4.41
ICONF	0.74	0.62	0.58	0.66	0.68	1.28	0.83	0.78	1.31	0.33
ACONF	0.35	0.37	0.37	0.46	0.40	0.21	0.13	0.07	0.17	0.23
Amino20x4	0.86	1.10	1.03	1.04	1.03	1.00	1.12	1.01	0.76	0.53
PCONF21	2.09	0.83	0.84	0.82	0.81	1.48	0.73	2.04	0.68	1.76
MCONF	1.93	0.56	0.64	0.60	0.59	0.88	0.51	1.15	0.59	0.26
SCONF	0.66	0.95	0.91	1.08	1.07	1.15	0.35	0.39	0.23	0.52
UPU23	2.92	0.97	0.93	0.97	1.00	1.61	1.46	1.19	1.72	2.40
BUT14DIOL	0.23	0.53	0.49	0.55	0.50	1.25	0.82	0.22	0.43	0.22
BH76RC	11.59	6.97	7.29	4.61	4.46	22.80	4.25	2.84	4.06	4.90
TOTAL	46.70	48.19	44.03	22.19	17.85					

Table S10: RMSD for the 55 subsets of GMTKN55 dataset. The data from PBE, B3LYP, M06-2X, ωB97X, and DSD-PBEP86 consider effects dispersion and were taken from Ref. 49. All values in kcal/mol.

**Subset**	**MP2**	**SCS-MP2**	**SCS-MP2***	**CD2-SCS-MP2**	**CD4-SCS-MP2**	**PBE**	**B3LYP**	**M06-2X**	**ωB97X**	**DSD-PBEP86**
W4-11	8.79	4.03	4.09	2.51	2.87	19.26	5.53	5.62	5.12	4.27
G21EA	3.19	3.87	4.04	4.44	4.13	4.11	2.48	2.08	1.92	1.80
G21IP	3.91	4.21	4.05	3.30	3.13	4.84	4.38	3.67	3.72	2.62
DIPCS10	6.24	6.29	7.04	5.49	6.07	5.99	5.93	3.58	7.24	4.36
PA26	3.01	1.63	1.58	1.96	1.68	2.91	3.62	1.52	4.06	1.40
SIE4x4	2.53	2.68	2.86	2.41	2.47	26.41	20.57	10.20	14.33	5.71
ALKBDE10	8.05	3.89	3.75	3.28	3.51	10.42	4.99	7.02	5.80	3.79
YBDE18	7.47	3.60	3.48	2.62	3.06	5.77	5.74	2.78	2.87	1.54
AL2X6	1.02	2.92	2.74	3.06	2.65	2.50	2.78	1.49	3.13	2.60
HEAVYSB11	3.76	2.99	2.99	3.49	3.01	3.97	3.69	9.67	2.59	2.17
NBPRC	1.40	1.85	1.63	1.88	1.46	3.00	3.03	1.13	2.57	1.35
ALK8	6.07	4.66	4.51	5.93	3.79	5.70	3.14	3.20	4.80	2.04
RC21	8.07	8.68	8.39	3.24	3.63	7.81	2.97	1.96	3.42	2.32
G2RC	3.80	2.45	2.30	2.23	2.35	8.39	3.32	2.51	4.77	1.98
FH51	3.60	2.23	2.33	2.32	2.38	4.49	3.51	1.55	2.42	1.15
TAUT15	0.94	0.91	0.74	0.80	0.76	2.32	1.38	0.94	0.91	0.50
DC13	10.78	5.13	4.99	5.37	3.97	12.22	13.11	10.38	8.25	3.74
MB16-43	24.87	20.52	18.06	12.32	9.10	30.59	28.92	18.56	41.78	23.17
DARC	4.05	1.03	0.92	1.27	1.36	3.65	8.26	2.40	1.74	1.55
RSE43	3.39	3.39	3.29	1.74	1.82	3.25	1.96	0.75	1.53	1.68
BSR36	5.61	0.93	1.18	1.25	0.79	3.79	3.87	3.26	4.90	3.95
CDIE20	0.68	0.28	0.32	3.45	2.78	1.78	1.12	0.83	0.92	0.64
ISO34	1.63	0.72	0.79	0.84	0.84	2.08	2.64	1.71	1.41	0.78
ISOL24	4.06	1.17	1.23	1.35	1.38	6.37	8.15	4.51	3.44	2.87
C60ISO	20.95	13.29	13.31	12.07	8.01	12.80	2.56	7.39	14.71	10.06
PArel	1.37	0.82	0.77	0.55	0.50	2.53	1.71	1.49	0.88	0.77
BH76	4.62	5.25	5.19	3.80	3.63	10.83	6.45	7.29	2.18	2.04
BHPERI	8.12	1.53	1.94	1.49	1.36	6.91	1.55	1.78	3.41	1.17
BHDIV10	2.99	1.81	1.69	2.45	2.08	9.87	3.78	1.32	1.27	1.49
INV24	2.51	1.83	1.79	2.25	2.16	2.66	1.37	2.14	2.41	1.00
BHROT27	0.28	0.20	0.17	0.22	0.22	0.65	0.60	0.52	0.54	0.25
PX13	2.73	1.33	1.16	2.04	1.75	12.24	4.46	5.85	2.04	2.35
WCPT18	2.65	1.53	1.28	2.00	1.86	9.85	2.94	2.32	1.87	1.62
RG18	0.24	0.37	0.36	0.49	0.54	0.32	0.20	0.32	0.31	0.30
ADIM6	0.33	1.09	1.00	1.18	1.00	0.22	0.13	0.38	0.42	1.55
S22	1.57	0.87	0.78	0.73	0.67	0.63	0.43	0.47	0.29	1.06
S66	0.83	0.69	0.60	0.76	0.52	0.52	0.39	0.28	0.30	0.91
HEAVY28	3.69	1.38	2.05	1.68	1.95	0.52	0.38	0.40	0.22	0.28
WATER27	1.33	8.28	7.44	4.10	3.73	11.86	5.48	4.34	3.13	1.67
CARBHB12	0.83	0.39	0.31	0.44	0.35	2.30	1.07	0.34	0.67	0.44
PNICO23	0.38	0.78	0.68	0.91	0.73	1.94	0.58	0.36	0.30	0.33
HAL59	7.07	3.90	2.48	1.97	1.86	1.86	0.84	0.46	0.50	0.49
AHB21	0.76	0.71	0.60	1.67	1.39	1.37	0.44	1.33	0.40	0.38
CHB6	1.25	0.86	0.87	1.35	0.75	1.01	1.48	1.66	1.43	0.66
IL16	0.57	1.50	1.39	1.97	0.91	0.67	0.92	0.53	1.27	1.10
IDISP	7.32	2.62	2.73	3.55	2.75	4.15	5.53	2.73	3.49	2.59
ICONF	0.25	0.30	0.27	0.28	0.27	0.45	0.40	0.43	0.58	0.22
ACONF	0.15	0.17	0.17	0.22	0.18	0.09	0.07	0.31	0.08	0.14
Amino20x4	0.29	0.34	0.32	0.33	0.32	0.42	0.29	0.37	0.32	0.22
PCONF21	1.06	0.32	0.33	0.35	0.34	1.50	0.61	1.26	0.37	0.95
MCONF	1.18	0.29	0.33	0.31	0.30	0.54	0.27	0.63	0.33	0.37
SCONF	0.39	0.50	0.45	0.56	0.53	1.00	0.51	0.33	0.35	0.18
UPU23	0.91	0.48	0.45	0.48	0.49	0.66	0.72	0.63	0.90	1.11
BUT14DIOL	0.13	0.34	0.30	0.33	0.30	0.52	0.34	0.19	0.14	0.15
BH76RC	4.03	1.93	2.09	1.26	1.45	6.12	2.79	1.71	2.01	1.38
TOTAL	6.29	4.66	4.29	3.15	2.67					

Table S11: WTMAD2 for the 55 subsets of GMTKN55 dataset. The data from PBE, B3LYP, M06-2X, ωB97X, and DSD-PBEP86 consider effects dispersion and were taken from Ref. 49. All values in kcal/mol.

Subset	MP2	SCS-MP2	SCS-MP2*	CD2-SCS-MP2	CD4-SCS-MP2	PBE	B3LYP	M06-2X	ωB97X	DSD-PBEP86
W4-11	1.30	0.57	0.61	0.33	0.41	2.90	0.63	0.59	0.53	0.61
G21EA	4.66	5.71	6.11	6.41	6.21	5.80	3.23	2.98	2.59	2.55
G21IP	0.63	0.67	0.68	0.58	0.56	0.85	0.78	0.58	0.63	0.46
DIPCS10	0.43	0.50	0.57	0.43	0.47	0.39	0.41	0.27	0.47	0.34
PA26	0.55	0.38	0.36	0.49	0.44	0.66	0.86	0.37	1.10	0.29
SIE4x4	2.71	3.88	4.09	3.41	3.44	39.98	30.44	14.61	20.81	8.29
ALKBDE10	3.55	1.64	1.68	1.34	1.54	3.56	2.48	2.70	2.50	1.80
YBDE18	7.39	3.64	3.18	2.42	2.66	5.69	5.44	2.77	2.78	1.42
AL2X6	1.27	4.57	4.30	4.72	4.14	2.58	4.29	1.43	4.78	3.99
HEAVYSB11	3.43	2.06	2.01	2.51	2.37	3.48	3.23	7.99	2.32	2.05
NBPRC	2.28	3.21	2.84	3.07	2.33	4.94	4.10	1.95	3.88	2.22
ALK8	2.90	3.05	2.93	3.84	2.64	3.76	2.25	2.10	3.25	1.43
RC21	10.77	12.11	11.78	3.60	4.35	10.91	3.89	2.60	4.74	2.96
G2RC	3.32	2.41	2.31	2.11	2.01	7.67	3.03	2.13	4.30	1.71
FH51	4.73	2.78	2.90	2.91	2.86	5.81	4.78	2.20	3.45	1.56
TAUT15	13.99	12.86	10.41	11.60	11.07	34.34	21.65	14.56	13.81	8.40
DC13	8.59	4.23	3.99	4.24	3.04	3.21	3.94	2.80	2.38	1.10
MB16-43	2.67	2.02	1.77	1.37	0.94	2.94	3.01	1.90	4.43	2.11
DARC	6.45	1.41	1.47	1.86	1.95	5.79	14.06	3.78	2.31	2.19
RSE43	15.60	17.34	16.93	8.61	9.30	21.98	12.86	4.71	10.39	6.43
BSR36	15.95	2.28	2.68	2.99	2.12	11.12	11.76	8.70	14.49	12.46
CDIE20	7.55	2.46	2.87	13.32	11.25	23.13	14.02	7.57	8.83	7.85
ISO34	4.35	1.88	2.05	2.28	2.22	5.81	6.94	4.80	4.02	2.07
ISOL24	7.49	2.30	2.33	2.78	2.89	11.38	15.04	7.11	6.85	4.88
C60ISO	9.46	5.95	5.88	5.42	3.59	6.20	1.28	3.98	7.84	4.55
PArel	11.36	6.73	6.22	5.20	4.86	22.22	14.48	11.91	7.12	6.14
BH76	10.78	13.20	13.08	9.91	9.36	29.38	17.41	7.15	5.74	3.66
BHPERI	20.98	3.47	4.59	2.64	2.76	18.22	3.21	3.68	8.33	2.94
BHDIV10	2.82	1.94	1.85	2.84	2.47	11.12	4.04	1.32	1.25	1.63
INV24	1.89	1.76	1.75	2.38	2.27	3.69	1.87	2.28	2.64	1.14
BHROT27	2.08	1.24	1.13	1.71	1.68	4.26	3.72	3.26	3.53	1.72
PX13	4.45	2.11	1.81	3.27	2.83	20.48	7.38	9.06	3.08	3.87
WCPT18	4.12	2.30	1.91	3.05	2.87	15.17	3.69	3.05	2.29	2.29
RG18	15.46	27.87	26.79	38.39	40.55	25.48	12.74	22.54	20.58	22.54
ADIM6	4.43	17.25	15.84	18.67	15.76	3.55	1.86	4.57	5.75	23.70
S22	7.80	4.96	4.45	4.59	3.86	3.74	2.41	2.65	1.63	6.23
S66	6.24	5.94	5.08	6.72	4.47	4.16	2.70	2.29	2.50	7.69
HEAVY28	43.09	28.24	33.81	33.29	32.77	19.23	15.57	15.11	8.70	11.45
WATER27	0.71	3.88	3.47	2.11	1.90	6.25	2.85	2.59	1.63	0.73
CARBHB12	5.80	3.05	2.37	3.71	2.64	17.99	8.29	2.35	5.37	2.83
PNICO23	3.42	9.13	8.07	10.31	8.07	17.56	6.38	3.86	3.19	3.72
HAL59	34.84	19.99	13.47	13.74	13.30	14.61	7.06	4.33	5.20	4.70
AHB21	1.24	1.69	1.43	3.02	2.44	2.91	0.83	2.40	0.76	0.71
CHB6	1.97	1.25	1.29	2.54	1.21	1.97	2.99	3.01	2.67	1.25
IL16	0.24	0.77	0.71	0.99	0.38	0.31	0.40	0.24	0.55	0.55
IDISP	23.83	7.58	7.64	10.92	7.44	11.03	14.27	8.27	10.71	8.83
ICONF	3.01	3.93	3.38	3.52	3.30	5.57	5.05	5.57	7.31	3.13
ACONF	3.68	4.51	4.33	5.94	4.70	2.17	1.55	8.37	2.17	3.72
Amino20x4	5.36	5.88	5.51	5.70	5.60	7.92	4.89	6.99	5.59	4.20
PCONF21	29.17	7.81	8.41	8.50	8.22	43.81	18.58	38.20	10.87	29.79
MCONF	11.92	2.86	3.36	3.05	2.93	5.60	2.52	6.29	3.43	3.66
SCONF	4.05	5.52	4.88	6.19	5.79	9.89	3.71	3.21	3.34	1.73
UPU23	6.31	3.54	3.29	3.61	3.73	5.26	6.06	4.97	7.65	8.44
BUT14DIOL	2.34	6.43	5.72	6.17	5.53	9.34	6.29	2.64	2.23	2.84
BH76RC	7.84	3.68	4.10	2.29	2.97	11.11	5.98	3.14	4.22	2.23
TOTAL	8.08	5.90	5.59	5.53	5.17	10.32	6.42	4.94	4.77	3.14

5 CD-SCS-MP2 *vs.* DFT

5.1 Mean Absolute Deviation Plots

Figures shown in this section include the performance of a series of DFAs reported in the literature for the GMTKN55 dataset. The following DFAs were considered:

- **Generalised-gradient-approximation (GGA) or non-separable gradient approximations (NGAs)**: PBE, PBEhPBE, RevPBE, RPBE, PW91, BLYP, BP86, BPBE, OPBE, OLYP, XLYP, mPWLYP, PW91P86, mPWPW91, rPW86PBE, B97, HCTH/407, and N12.

- **Meta-GGA and meta-NGA DFAs** : PKZB, TPSS, revTPSS, SCAN, τHCTH, M06L, M11L, MN12L, and MN15L.

- **Hybrids DFAs**: B3LYP, B3PW91, B3P86, BHLYP, B1P86, B1LYP, B1B95, MPW1B95, PW6B95, MPWB1K, mPW1LYP, MPW1PW91, PW1PW, MPW1KCIS, MPWKCIS1K, PBE0, PBEh1PBE, PBE1KCIS, X3LYP, O3LYP, B97-1,B97-2, B98, HISS, HSE03, HSE06, TPSSh, revTPSSh, TPSS0, revTPSS0, TPSS1KCIS, BMK, τHCTHhyb, M05, M05-2X, M06, M06-2X, M08-HX, M11, SOGGA11X, N12SX, MN12SX, MN15, and LC-ωhPBE.

- **Double-hydrids DFAs**: B2PLYP, B2GPPLYP, MPW2PLYP, PWPB95, DSD-BLYP, DSD-PBEP86, and DSD-PBEB95.

For the thermochemical properties, it is observed that the density functional approximations (DFAs) DSD-PBE-P86, DSD-BLYP and B2GPPLYP show slightly better performance than CD-SCS-MP2 methods, however, the first two correspond to DFA double hybrids, are based on spin-component-scaled (SCS), and depend on multiple parameters. In addition, B2GPPLYP was developed to reduce the errors of the properties described in the GMTKN30 dataset (precursor of the GMTKN55 dataset), so the behavior seen in the plots is somewhat

expected. It is important to highlight that HCTH/407 shows the most important deviations. In the case of reaction energies for large systems and isomerization energy, some double hybrid DFAs show better performance, however CD-SCS-MP2 methods exhibit a small MAD. Regarding the reaction barriers, the DFA DSD-PBEB95 has the lowest MAD, however, with CD-SCS-MP2 and some DFAs acceptable errors are obtained.

Table S12: Error assessment of MP2, SCS-MP2, CD2-SCS-MP2, CD4-SCS-MP2, DSD-PBEP86-D3(BJ), DSD-BLYP-D3(BJ), B2PLYP-D3(BJ), PBE0-DH-D3(BJ) and PBE-QIQH-D3(BJ) for the training set [the diet-GMTKN55 dataset (150 data points)]. All values in kcal/mol.

**Error type**	MP2	SCS-MP2	CD2-SCS-MP2	CD4-SCS-MP2	DSD-PBEP86-D3(BJ)	DSD-BLYP-D3(BJ)	B2PLYP-D3(BJ)	PBE0-DH-D3(BJ)	PBE-QIQH-D3(BJ)
**MAD**	3.17	2.27	1.36	1.18	1.17	1.18	1.62	3.00	1.91
**MAX**	37.74	26.38	22.19	14.95	18.19	18.91	19.78	55.93	18.47
**RMSD**	6.10	4.45	2.92	2.23	2.30	2.44	3.33	6.28	3.31
**WTMAD2**	6.72	5.12	5.75	5.52	5.51	5.53	5.98	6.65	6.09

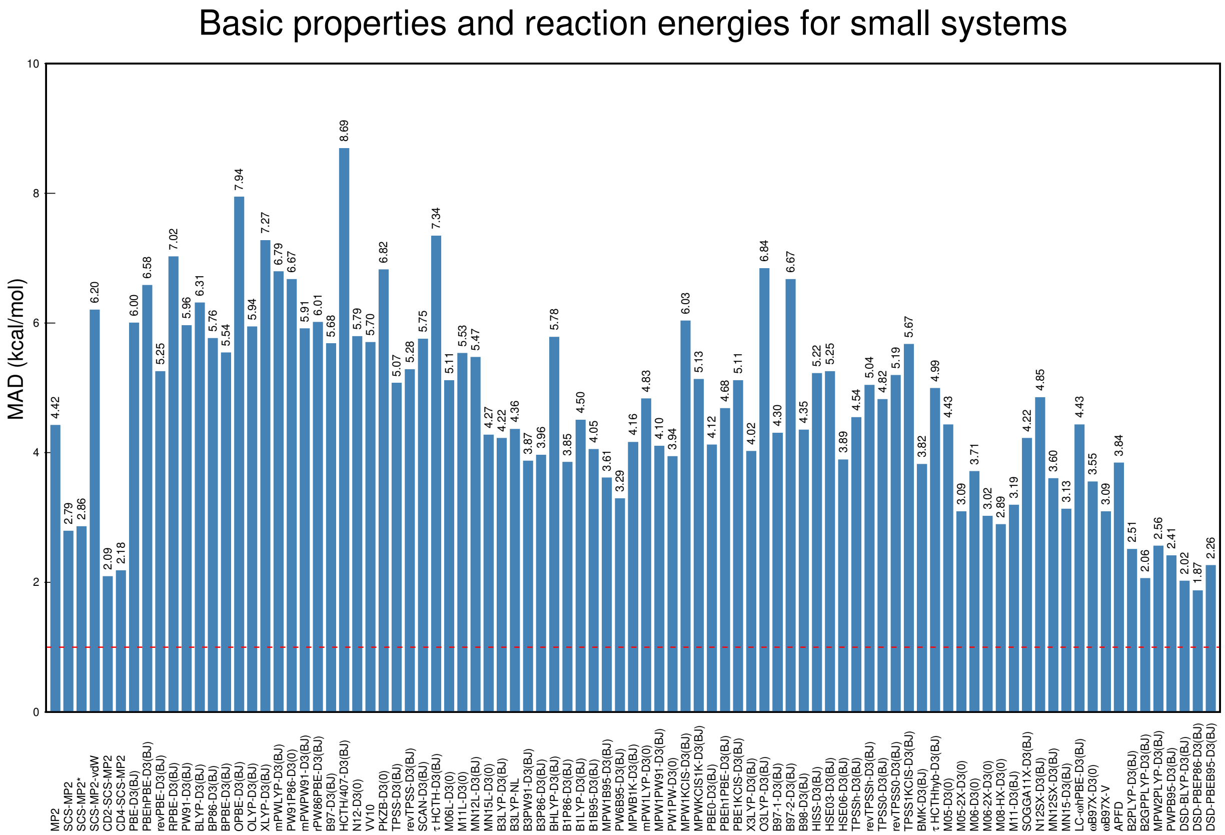

Figure S3: MAD of basic properties and reaction energies for small systems. 718 single point calculations and 473 relative energies for each of the methodologies. 18 sets out of 55 were considered. Data for all the DFAs has been extracted from Ref. S12. The dotted line is over 1 kcal/mol, desired chemical precision in electronic structure.

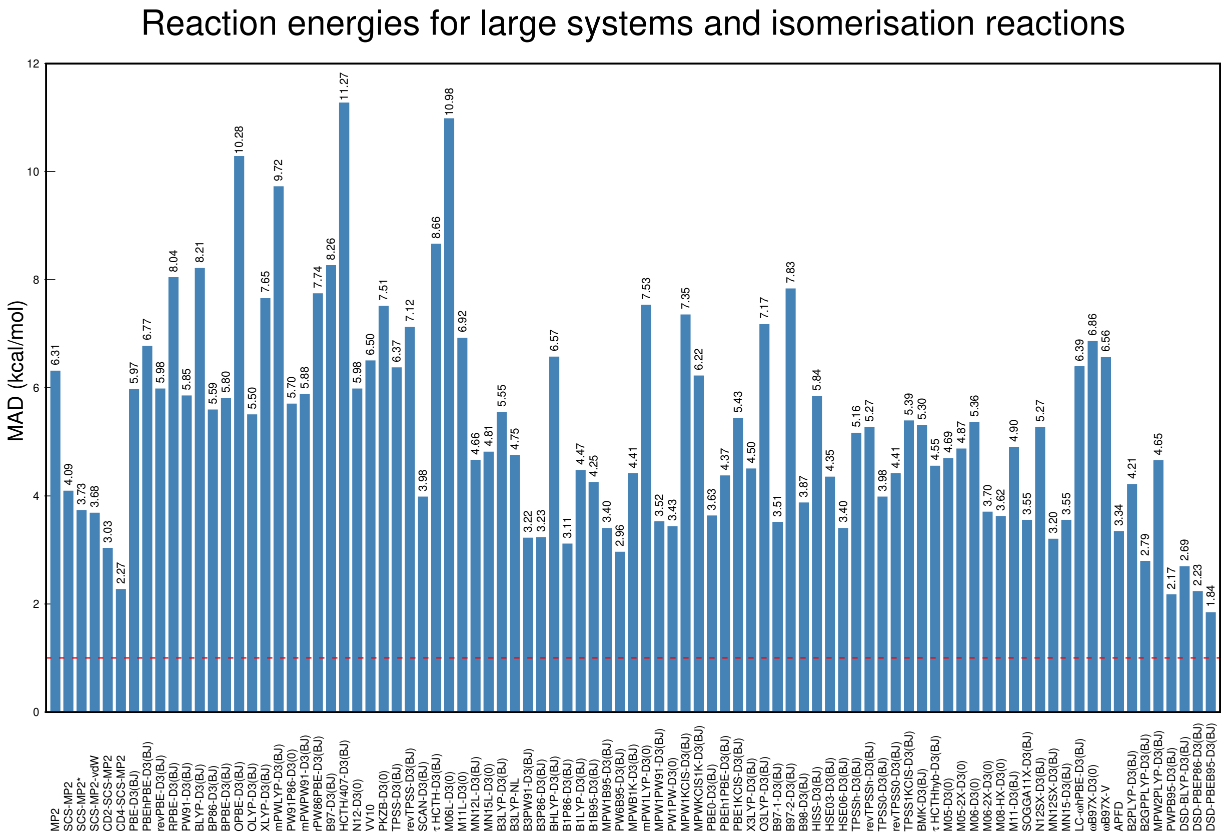

Figure S4: MAD of reaction energies for large systems and isomerisation reaction. 394 single point calculations and 243 relative energies for each of the methodologies. 9 sets out of 55 were considered. Data for all the DFAs has been extracted from Ref. S12. The dotted line is over 1 kcal/mol, desired chemical precision in electronic structure.

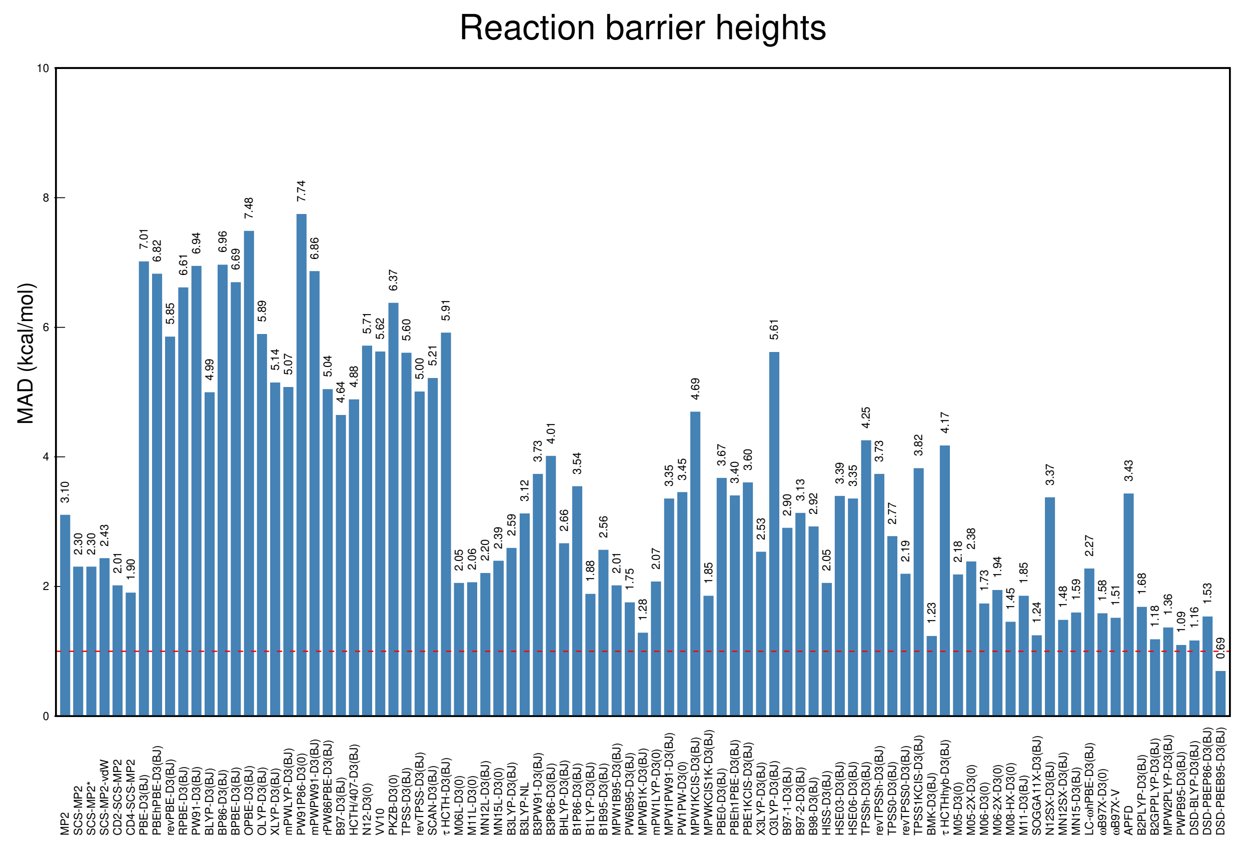

Figure S5: MAD of reaction barriers heights. 312 single point calculations and 194 relative energies for each of the methodologies. 7 sets out of 55 were considered. Data for all the DFAs has been extracted from Ref. S12. The dotted line is over 1 kcal/mol, desired chemical precision in electronic structure.

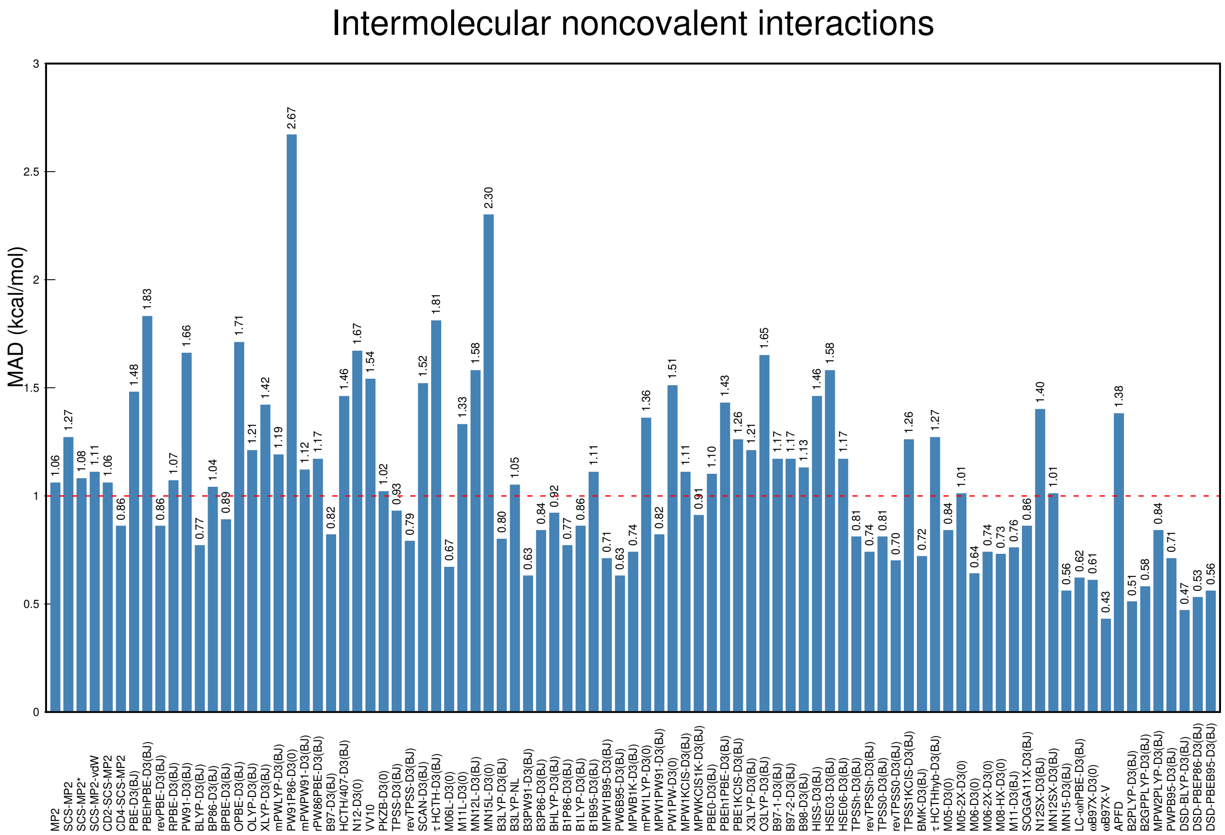

Figure S6: MAD of intermolecular noncovalent interactions. 699 single point calculations and 304 relative energies for each of the methodologies. 12 sets out of 55 were considered.Data for all the DFAs has been extracted from Ref. S12. The dotted line is over 1 kcal/mol, desired chemical precision in electronic structure.

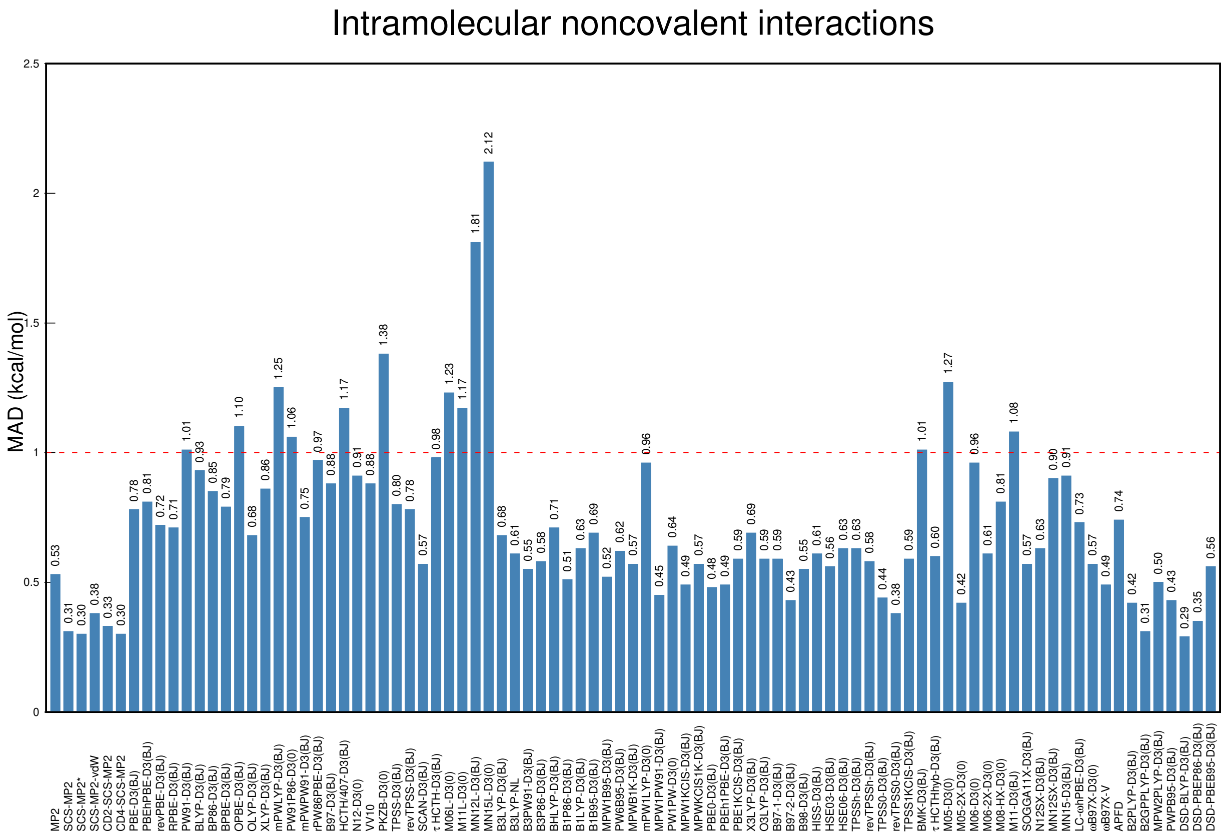

Figure S7: MAD of intramolecular noncovalent interactions. 339 single point calculations and 291 relative energies for each of the methodologies. 9 sets out of 55 were considered. Data for all DFAs has been extracted from Ref. S12. The dotted line is over 1 kcal/mol, desired chemical precision in electronic structure.

5.2 Root-Mean-Square Deviation Plots

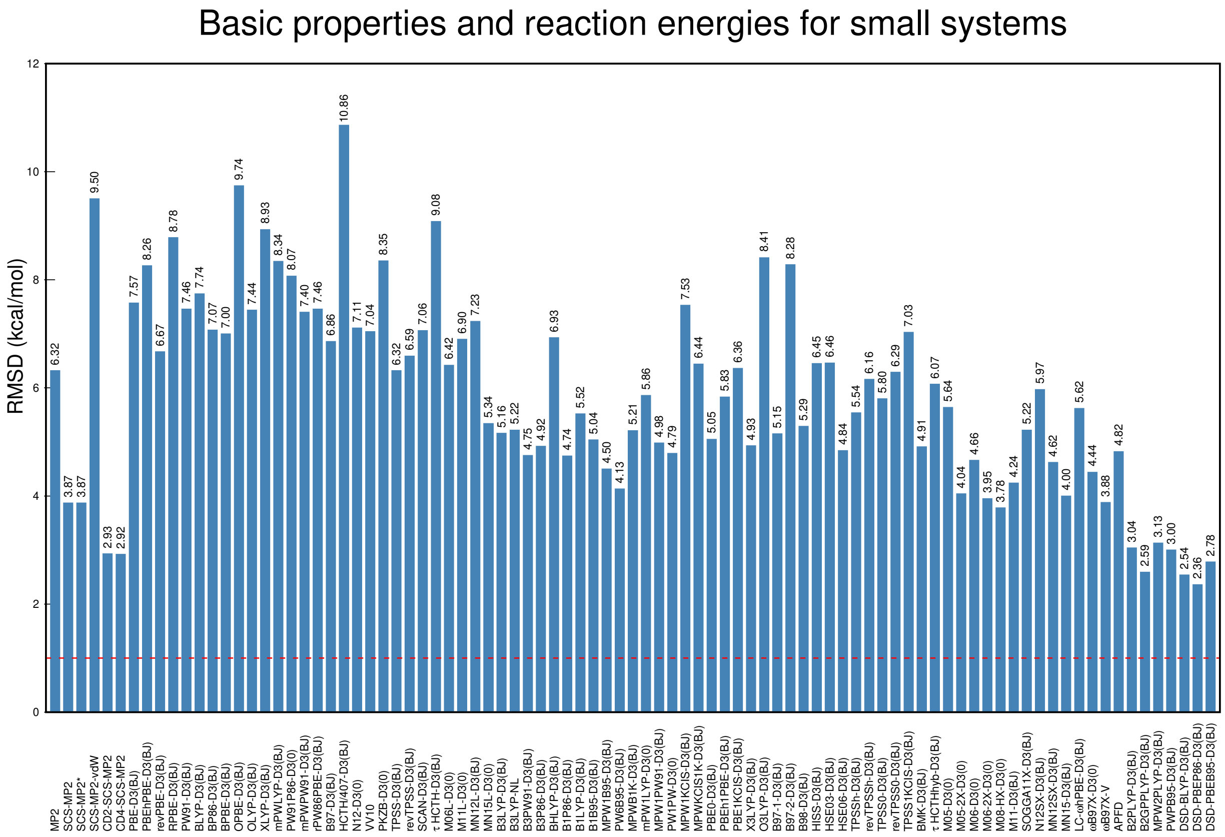

Figure S8: RMSD of basic properties and reaction energies for small systems. 718 single point calculations and 473 relative energies for each of the methodologies. 18 sets out of 55 were considered. Data for all the DFAs has been extracted from Ref. S12. The dotted line is over 1 kcal/mol, desired chemical precision in electronic structure.

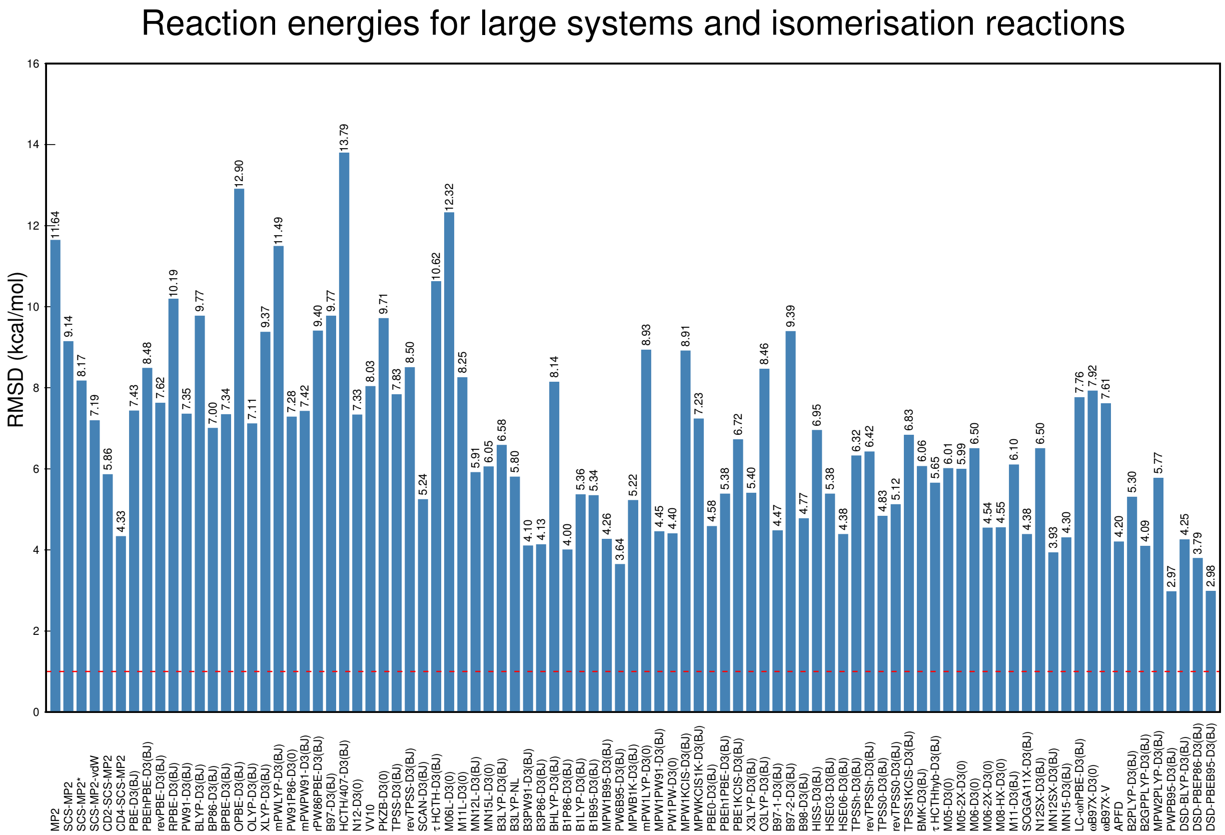

Figure S9: RMSD of reaction energies for large systems and isomerisation reaction. 394 single point calculations and 243 relative energies for each of the methodologies. 9 sets out of 55 were considered. Data for all the DFAs has been extracted from Ref. S12. The dotted line is over 1 kcal/mol, desired chemical precision in electronic structure.

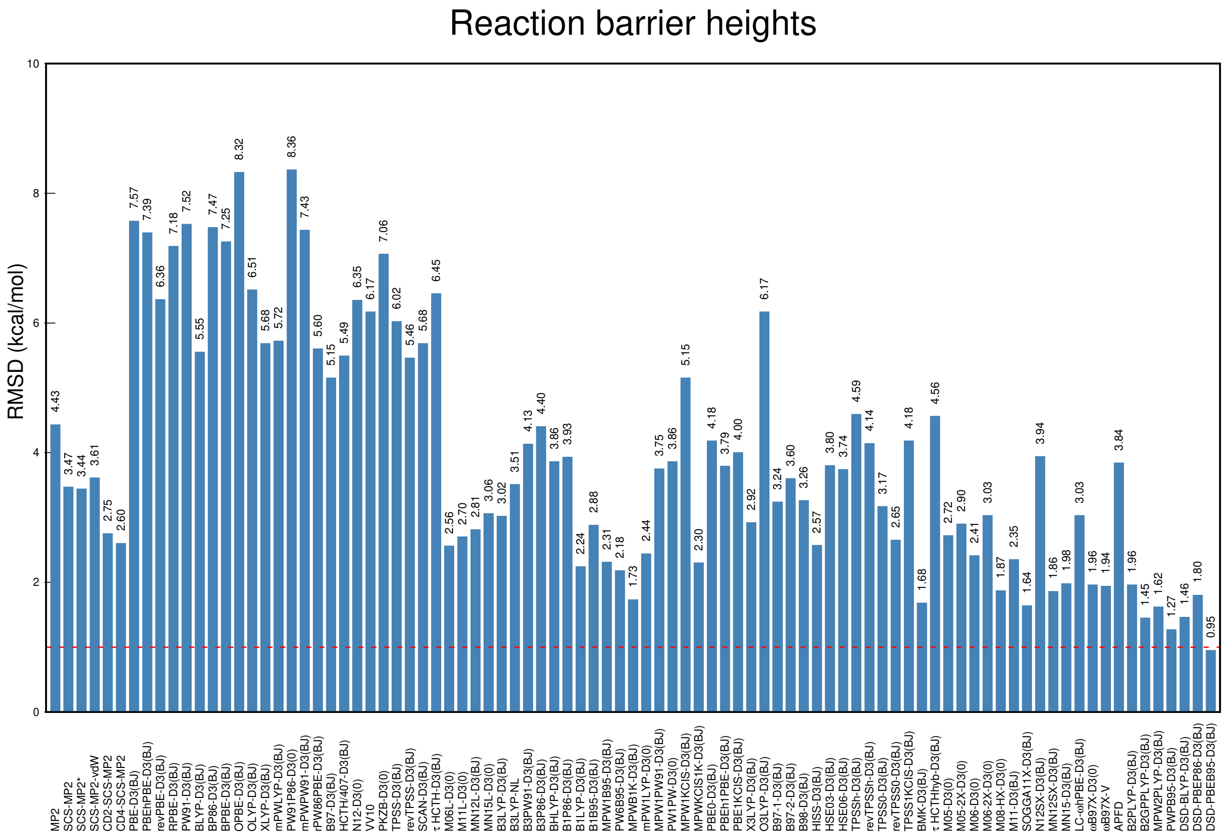

Figure S10: RMSD of reaction barriers heights. 312 single point calculations and 194 relative energies for each of the methodologies. 7 sets out of 55 were considered. Data for all the DFAs has been extracted from Ref. S12. The dotted line is over 1 kcal/mol, desired chemical precision in electronic structure.

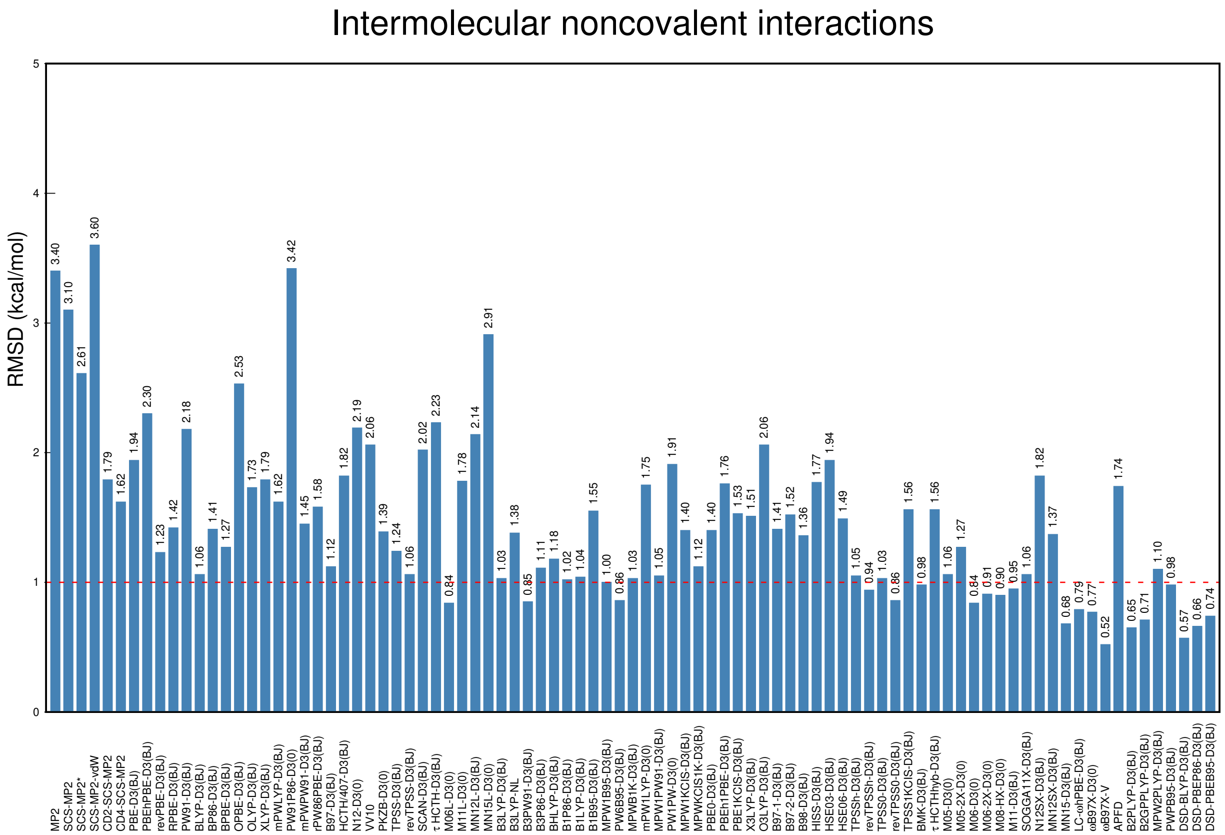

Figure S11: RMSD of intermolecular noncovalent interactions. 699 single point calculations and 304 relative energies for each of the methodologies. 12 sets out of 55 were considered.Data for all the DFAs has been extracted from Ref. S12. The dotted line is over 1 kcal/mol, desired chemical precision in electronic structure.

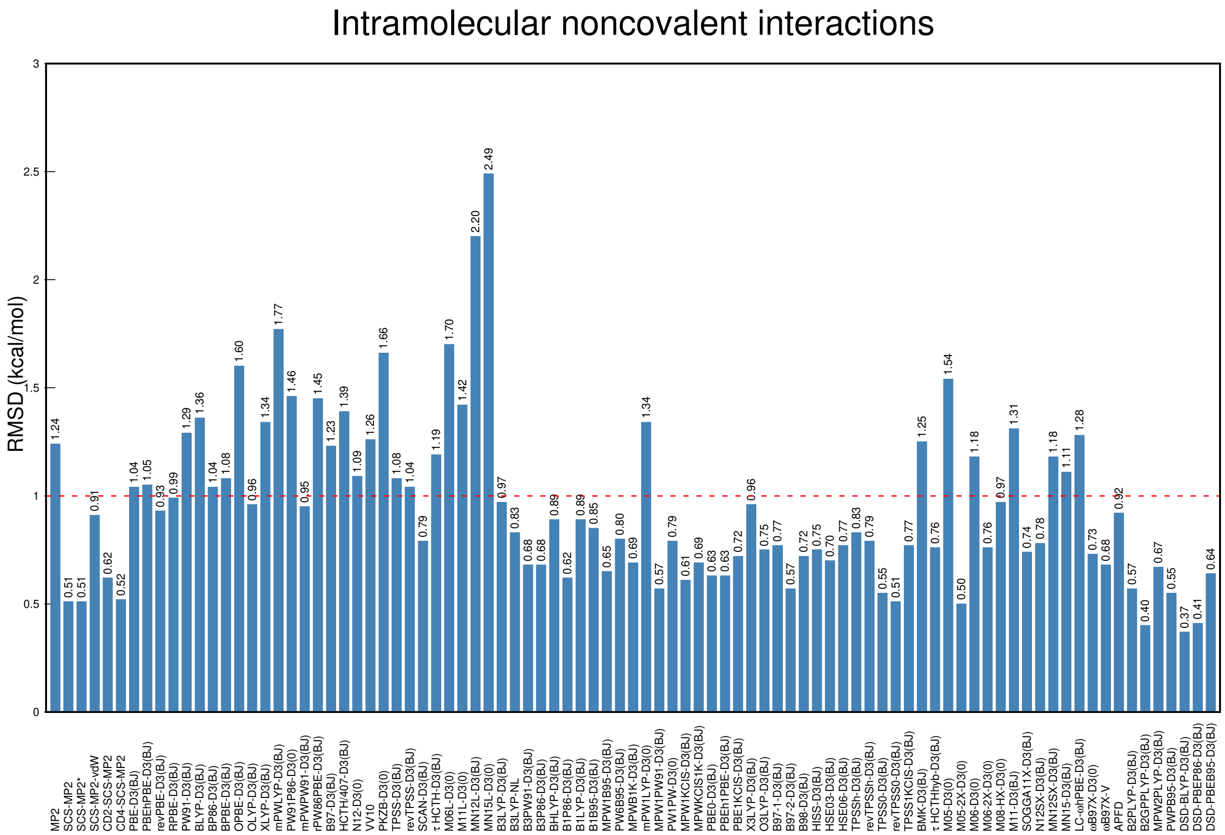

Figure S12: RMSD of intramolecular noncovalent interactions. 339 single point calculations and 291 relative energies for each of the methodologies. 9 sets out of 55 were considered. Data for all DFAs has been extracted from Ref. S12. The dotted line is over 1 kcal/mol, desired chemical precision in electronic structure.

5.3 Maximum Mean Absolute Deviation Plots

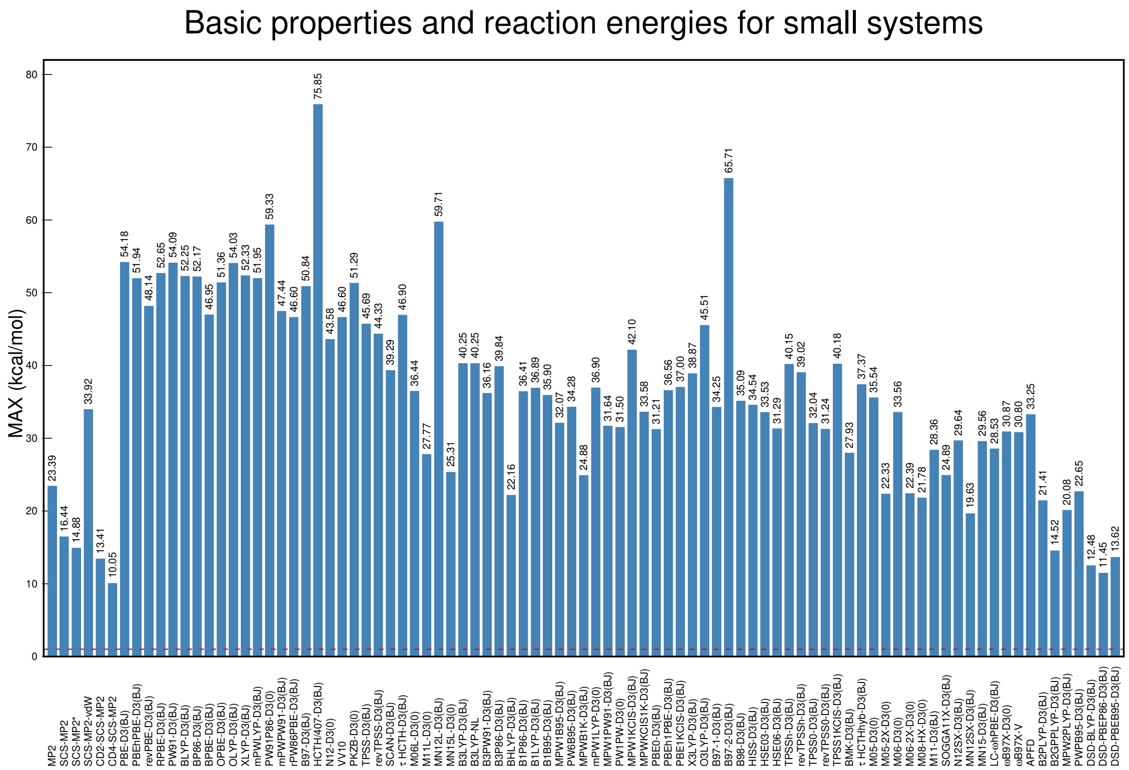

Figure S13: MAX of basic properties and reaction energies for small systems. 718 single point calculations and 473 relative energies for each of the methodologies. 18 sets out of 55 were considered. Data for all the DFAs has been extracted from Ref. S12. The dotted line is over 1 kcal/mol, desired chemical precision in electronic structure.

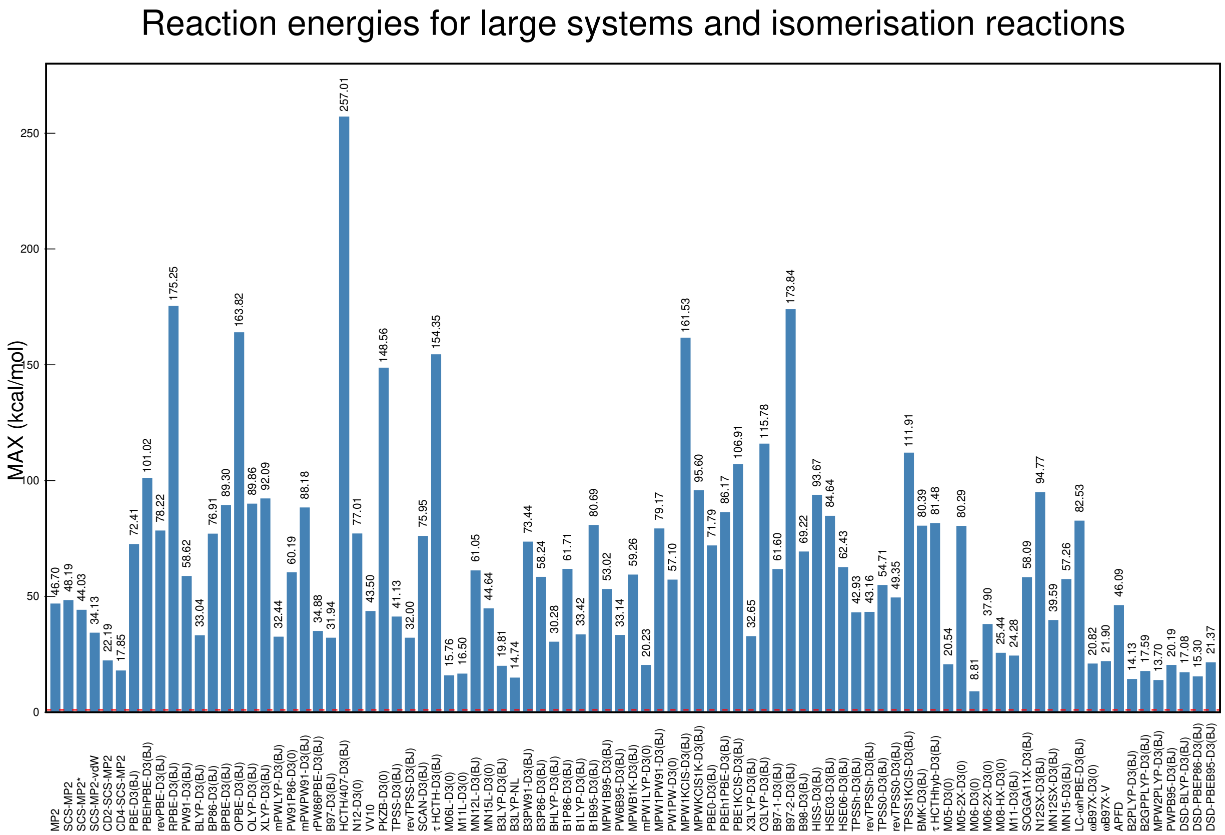

Figure S14: MAX of reaction energies for large systems and isomerisation reaction. 394 single point calculations and 243 relative energies for each of the methodologies. 9 sets out of 55 were considered. Data for all the DFAs has been extracted from Ref. S12. The dotted line is over 1 kcal/mol, desired chemical precision in electronic structure.

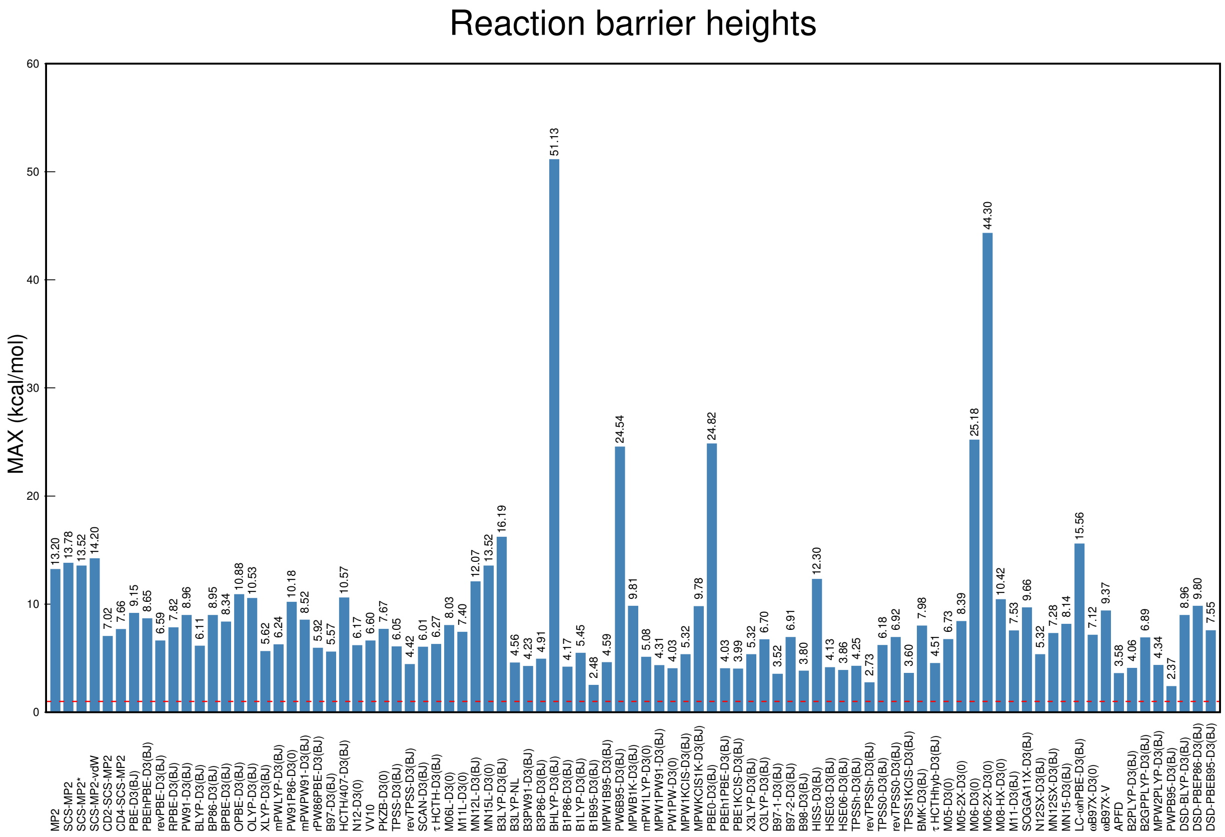

Figure S15: MAX of reaction barriers heights. 312 single point calculations and 194 relative energies for each of the methodologies. 7 sets out of 55 were considered. Data for all the DFAs has been extracted from Ref. S12. The dotted line is over 1 kcal/mol, desired chemical precision in electronic structure.

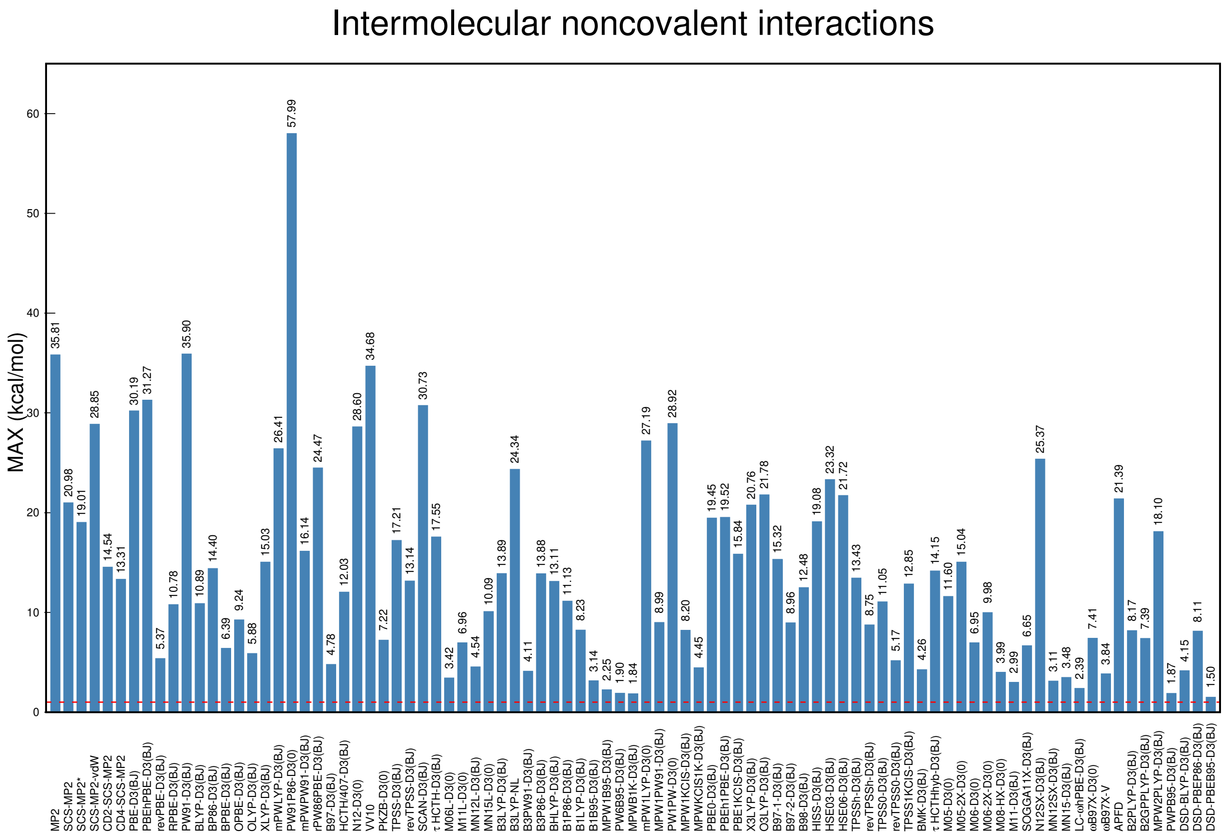

Figure S16: MAX of intermolecular noncovalent interactions. 699 single point calculations and 304 relative energies for each of the methodologies. 12 sets out of 55 were considered.Data for all the DFAs has been extracted from Ref. S12. The dotted line is over 1 kcal/mol, desired chemical precision in electronic structure.

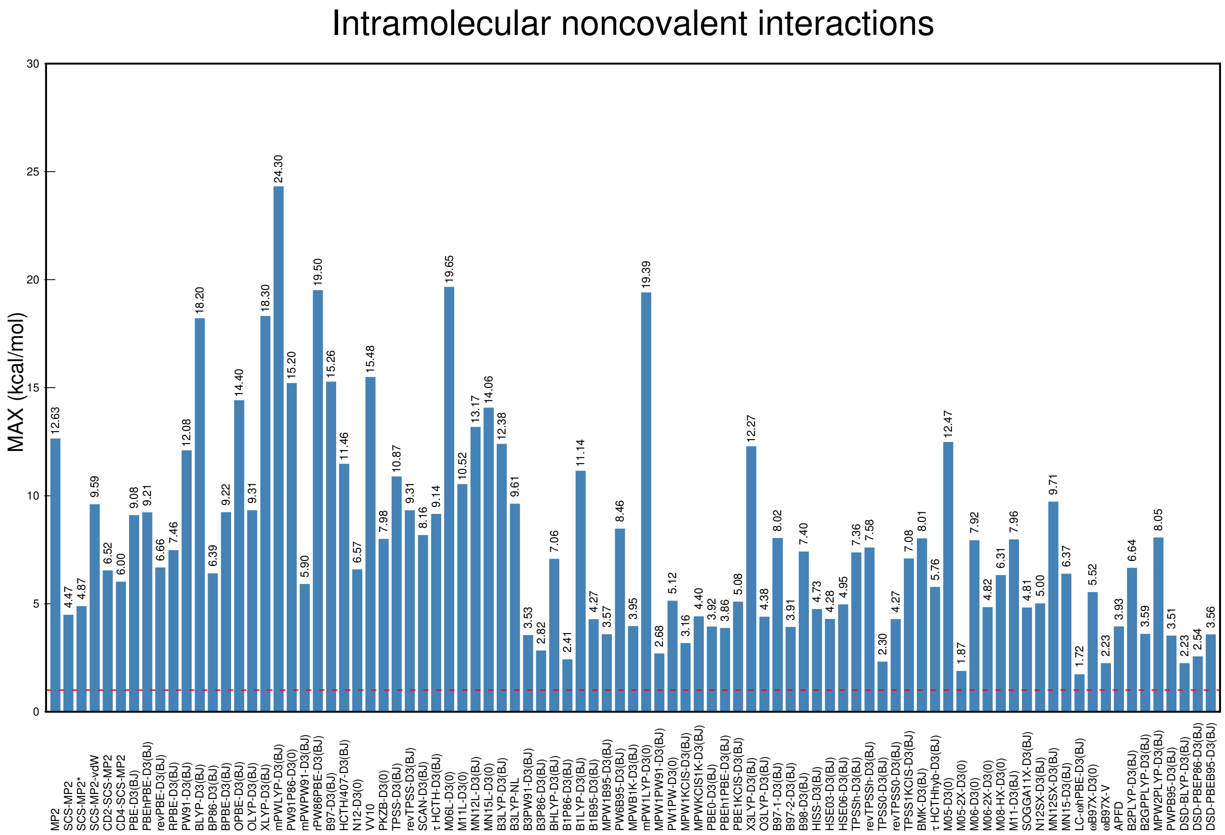

Figure S17: MAX of intramolecular noncovalent interactions. 339 single point calculations and 291 relative energies for each of the methodologies. 9 sets out of 55 were considered. Data for all DFAs has been extracted from Ref. S12. The dotted line is over 1 kcal/mol, desired chemical precision in electronic structure.

5.4 Weighted total mean absolute deviation of type 2 Plots

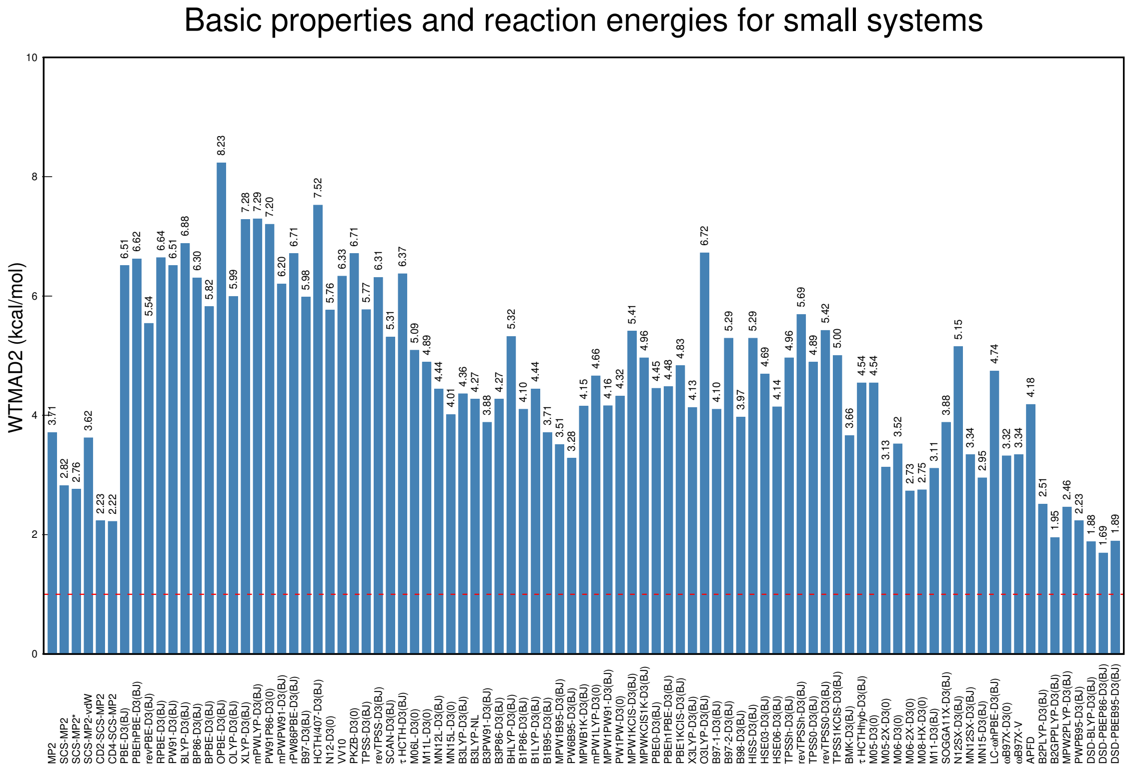

Figure S18: WTMAD2 of basic properties and reaction energies for small systems. 718 single point calculations and 473 relative energies for each of the methodologies. 18 sets out of 55 were considered. Data for all the DFAs has been extracted from Ref. S12. The dotted line is over 1 kcal/mol, desired chemical precision in electronic structure.

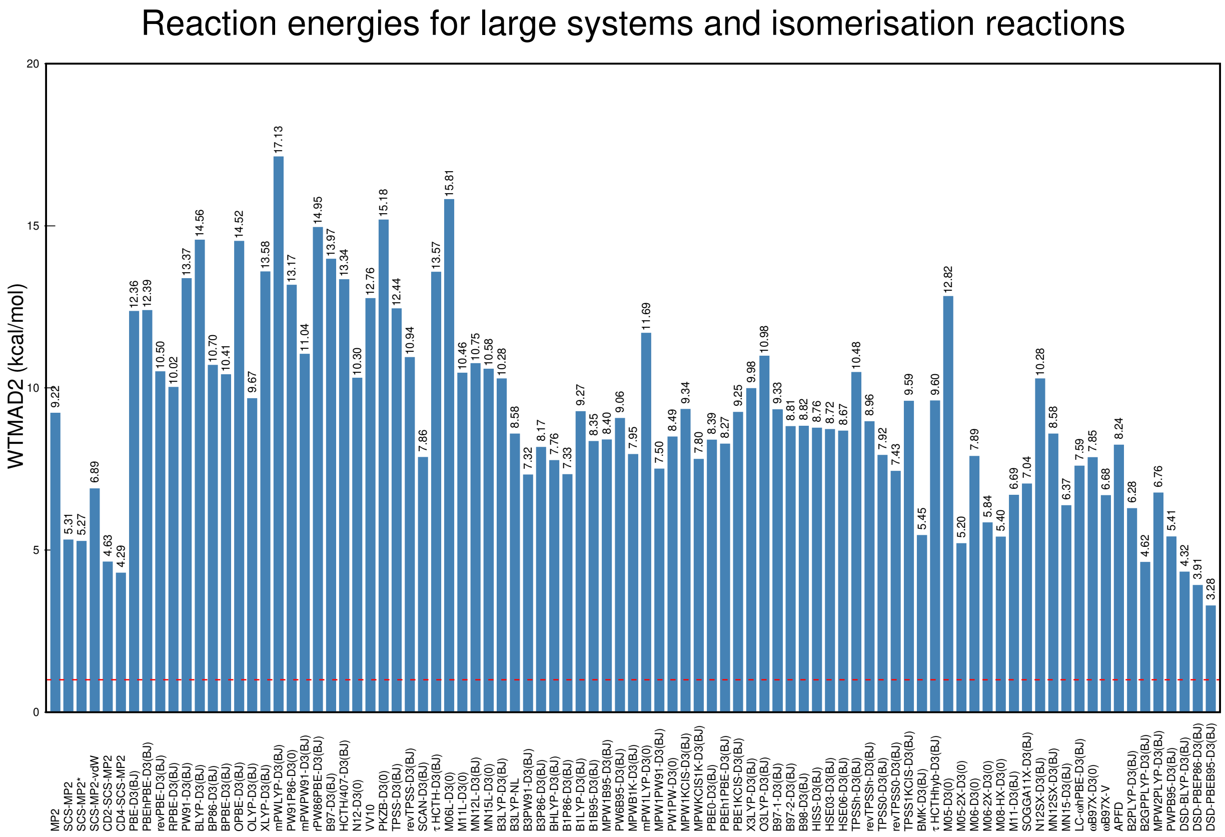

Figure S19: WTMAD2 of reaction energies for large systems and isomerisation reaction. 394 single point calculations and 243 relative energies for each of the methodologies. 9 sets out of 55 were considered. Data for all the DFAs has been extracted from Ref. S12. The dotted line is over 1 kcal/mol, desired chemical precision in electronic structure.

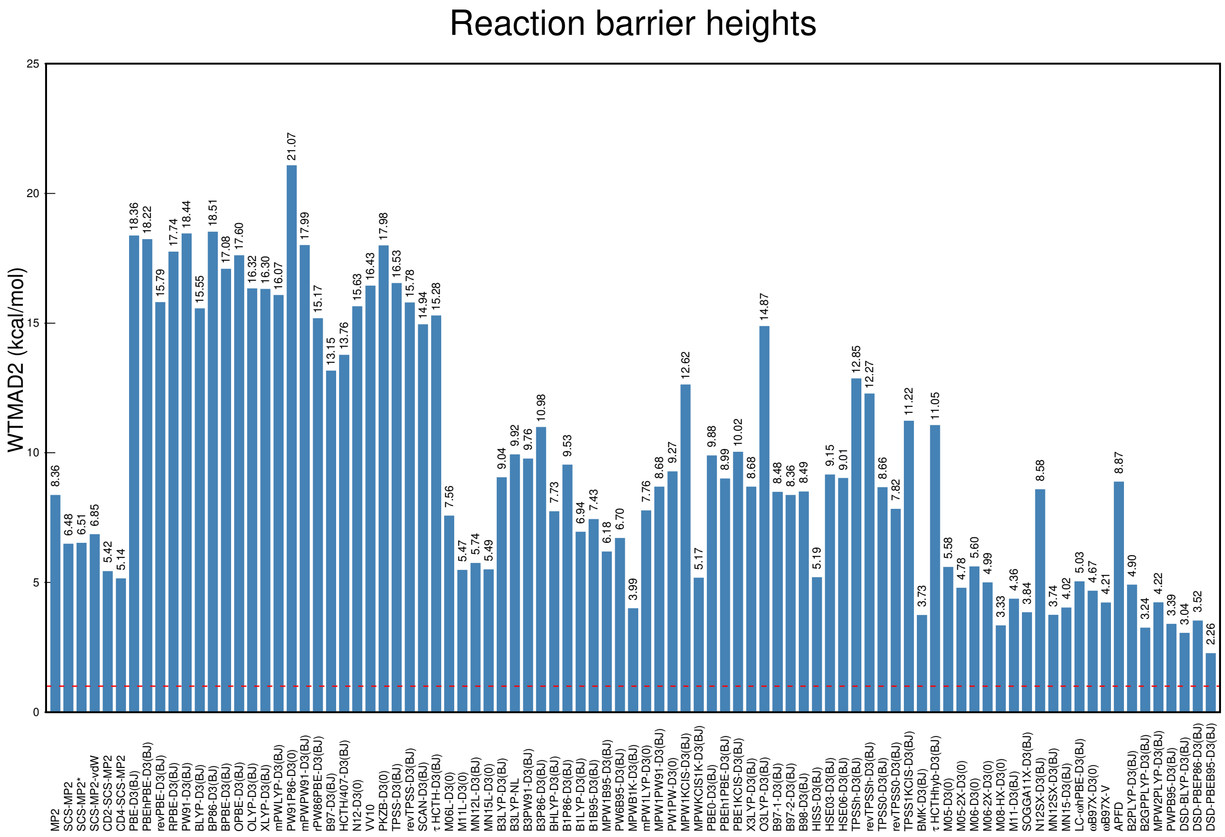

Figure S20: WTMAD2 of reaction barriers heights. 312 single point calculations and 194 relative energies for each of the methodologies. 7 sets out of 55 were considered. Data for all the DFAs has been extracted from Ref. S12. The dotted line is over 1 kcal/mol, desired chemical precision in electronic structure.

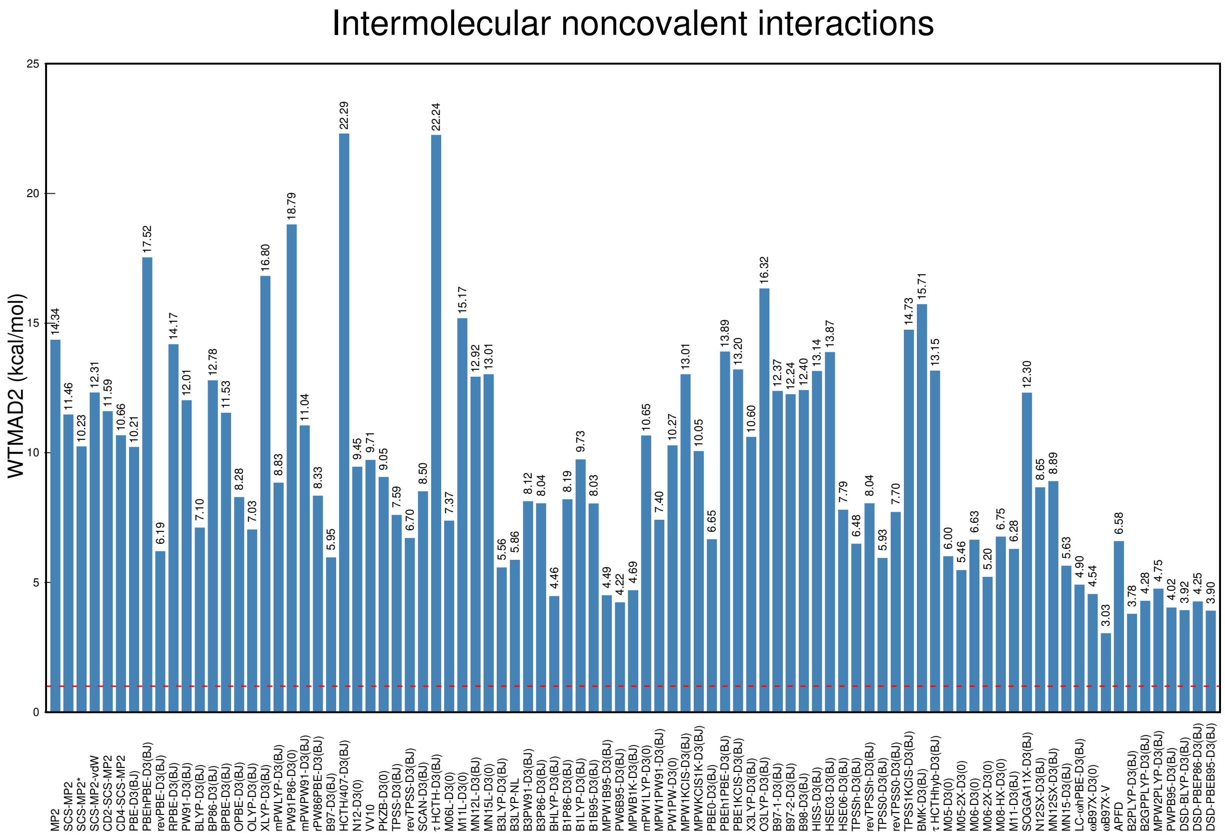

Figure S21: WTMAD2 of intermolecular noncovalent interactions. 699 single point calculations and 304 relative energies for each of the methodologies. 12 sets out of 55 were considered.Data for all the DFAs has been extracted from Ref. S12. The dotted line is over 1 kcal/mol, desired chemical precision in electronic structure.

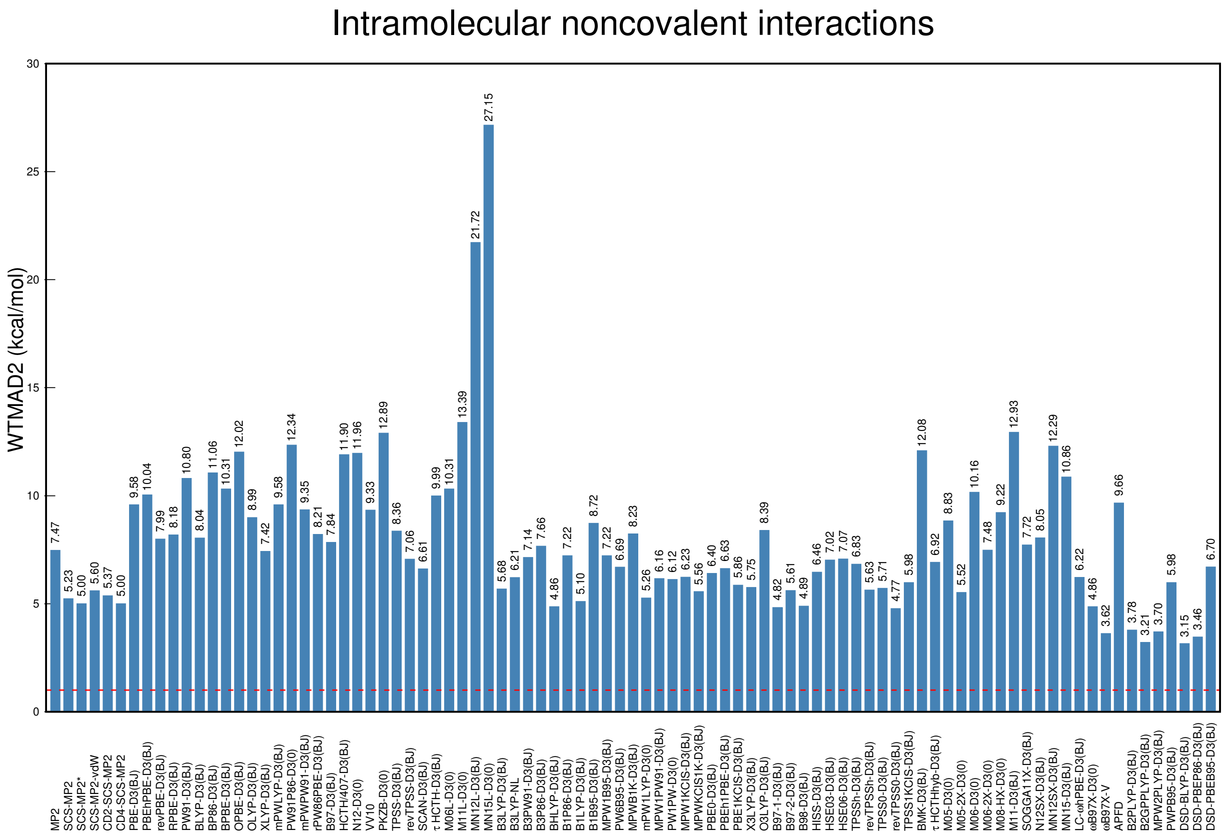

Figure S22: WTMAD2 of intramolecular noncovalent interactions. 339 single point calculations and 291 relative energies for each of the methodologies. 9 sets out of 55 were considered. Data for all DFAs has been extracted from Ref. S12. The dotted line is over 1 kcal/mol, desired chemical precision in electronic structure.

References

(S1) Ramos-Cordoba, E.; Salvador, P.; Matito, E. Separation of dynamic and nondynamic correlation. *Phys. Chem. Chem. Phys.* **2016**, *18*, 24015–24023.

(S2) Ramos-Cordoba, E.; Matito, E. Local Descriptors of dynamic and nondynamic correlation. *J. Chem. Theory Comput.* **2017**, *13*, 2705–2711.

(S3) Via-Nadal, M.; Rodríguez-Mayorga, M.; Ramos-Cordoba, E.; Matito, E. Singling out Weak and Strong Correlation. *J. Phys. Chem. Lett.* **2019**, *10*, 4032–4037.

(S4) Via-Nadal, M.; Rodríguez-Mayorga, M.; Ramos-Cordoba, E.; Matito, E. Range Separation of the Coulomb Hole. *J. Chem. Phys.* **2022**, *156*, 184106.

(S5) Xu, X.; Soriano-Agueda, L.; López, X.; Ramos-Cordoba, E.; Matito, E. An All-Purpose Measure of Electron Correlation for Multireference Diagnostics. *J. Chem. Theory Comput.* **2024**, *20*, 721–727.

(S6) Xu, X.; Soriano-Agueda, L.; López, X.; Ramos-Cordoba, E.; Matito, E. How many distinct and reliable multireference diagnostics are there? *J. Chem. Phys.* **2025**, *162*, 124102.

(S7) Grimme, S. Improved second-order Møller–Plesset perturbation theory by separate scaling of parallel-and antiparallel-spin pair correlation energies. *J. Chem. Phys.* **2003**, *118*, 9095–9102.

(S8) Gould, T. 'Diet GMTKN55' offers accelerated benchmarking through a representative subset approach. *Phys. Chem. Chem. Phys.* **2018**, *20*, 27735–27739.

(S9) Karton, A.; Daon, S.; Martin, J. M. W4-11: A high-confidence benchmark dataset for computational thermochemistry derived from first-principles W4 data. *Chem. Phys. Lett.* **2011**, *510*, 165–178.

(S10) Goerigk, L.; Grimme, S. A General Database for Main Group Thermochemistry, Kinetics, and Noncovalent Interactions - Assessment of Common and Reparameterized (meta-)GGA Density Functionals. *J. Chem. Theory Comput.* **2010**, *6*, 107–126, PMID: 26614324.

(S11) Curtiss, L. A.; Raghavachari, K.; Trucks, G. W.; Pople, J. A. Gaussian-2 theory for molecular energies of first- and second-row compounds. *J. Chem. Phys.* **1991**, *94*, 7221–7230.

(S12) Goerigk, L.; Hansen, A.; Bauer, C.; Ehrlich, S.; Najibi, A.; Grimme, S. A look at the density functional theory zoo with the advanced GMTKN55 database for general main group thermochemistry, kinetics and noncovalent interactions. *Phys. Chem. Chem. Phys.* **2017**, *19*, 32184–32215.

(S13) Karton, A.; Rabinovich, E.; Martin, J. M. L.; Ruscic, B. W4 theory for computational thermochemistry: In pursuit of confident sub-kJ/mol predictions. *J. Chem. Phys.* **2006**, *125*, 144108.

(S14) Yu, H.; Truhlar, D. G. Components of the Bond Energy in Polar Diatomic Molecules, Radicals, and Ions Formed by Group-1 and Group-2 Metal Atoms. *J. Chem. Theory Comput.* **2015**, *11*, 2968–2983, PMID: 26575734.

(S15) Zhao, Y.; Ng, H. T.; Peverati, R.; Truhlar, D. G. Benchmark Database for Ylidic Bond Dissociation Energies and Its Use for Assessments of Electronic Structure Methods. *J. Chem. Theory Comput.* **2012**, *8*, 2824–2834, PMID: 26592123.

(S16) Goerigk, L.; Grimme, S. Efficient and Accurate Double-Hybrid-Meta-GGA Density Functionals—Evaluation with the Extended GMTKN30 Database for General Main Group Thermochemistry, Kinetics, and Noncovalent Interactions. *J. Chem. Theory Comput.* **2011**, *7*, 291–309, PMID: 26596152.

(S17) Grimme, S.; Kruse, H.; Goerigk, L.; Erker, G. The Mechanism of Dihydrogen Activation by Frustrated Lewis Pairs Revisited. *Angew. Chem., Int. Ed.* **2010**, *49*, 1402–1405.

(S18) Gruzman, D.; Karton, A.; Martin, J. M. L. Performance of Ab Initio and Density Functional Methods for Conformational Equilibria of CnH2n+2 Alkane Isomers (n = 4-8). *J. Phys. Chem. A* **2009**, *113*, 11974–11983, PMID: 19795892.

(S19) Curtiss, L. A.; Raghavachari, K.; Redfern, P. C.; Pople, J. A. Assessment of Gaussian-2 and density functional theories for the computation of enthalpies of formation. *J. Chem. Phys.* **1997**, *106*, 1063–1079.

(S20) Friedrich, J.; Hänchen, J. Incremental CCSD(T)(F12*)—MP2: A Black Box Method To Obtain Highly Accurate Reaction Energies. *J. Chem. Theory Comput.* **2013**, *9*, 5381–5394, PMID: 26592276.

(S21) Friedrich, J. Efficient Calculation of Accurate Reaction Energies—Assessment of Different Models in Electronic Structure Theory. *J. Chem. Theory Comput.* **2015**, *11*, 3596–3609, PMID: 26574443.

(S22) Zhao, Y.; Truhlar, D. G. The M06 suite of density functionals for main group thermochemistry, thermochemical kinetics, noncovalent interactions, excited states, and transition elements: two new functionals and systematic testing of four M06 functionals and 12 other functionals. *Theor. Chem. Acc.* **2008**, *119*, 525–525.

(S23) Grimme, S. Semiempirical hybrid density functional with perturbative second-order correlation. *J. Chem. Phys.* **2006**, *124*, 034108.

(S24) Grimme, S.; Mück-Lichtenfeld, C.; Würthwein, E.-U.; Ehlers, A. W.; Goumans, T. P. M.; Lammertsma, K. Consistent Theoretical Description of 1,3-Dipolar Cycloaddition Reactions. *J. Phys. Chem. A* **2006**, *110*, 2583–2586, PMID: 16494365.

(S25) Piacenza, M.; Grimme, S. Systematic quantum chemical study of DNA-base tautomers. *J. Comput. Chem.* **2004**, *25*, 83–99.

(S26) Woodcock, H. L.; Schaefer, H. F.; Schreiner, P. R. Problematic Energy Differences between Cumulenes and Poly-ynes: Does This Point to a Systematic Improvement of Density Functional Theory? *J. Phys. Chem. A* **2002**, *106*, 11923–11931.

(S27) Schreiner, P. R.; Fokin, A. A.; Pascal, R. A.; de Meijere, A. Many Density Functional Theory Approaches Fail To Give Reliable Large Hydrocarbon Isomer Energy Differences. *Org. Lett.* **2006**, *8*, 3635–3638, PMID: 16898779.

(S28) Lepetit, C.; Chermette, H.; Gicquel, M.; Heully, J.-L.; Chauvin, R. Description of Carbo-oxocarbons and Assessment of Exchange-Correlation Functionals for the DFT Description of Carbo-mers. *J. Phys. Chem. A* **2007**, *111*, 136–149, PMID: 17201396.

(S29) Lee, J. S. Accurate ab Initio Binding Energies of Alkaline Earth Metal Clusters. *J. Phys. Chem. A* **2005**, *109*, 11927–11932, PMID: 16366644.

(S30) Karton, A.; Martin, J. M. Explicitly correlated benchmark calculations on C8H8 isomer energy separations: how accurate are DFT, double-hybrid, and composite ab initio procedures? *Mol. Phys.* **2012**, *110*, 2477–2491.

(S31) Zhao, Y.; Tishchenko, O.; Gour, J. R.; Li, W.; Lutz, J. J.; Piecuch, P.; Truhlar, D. G. Thermochemical Kinetics for Multireference Systems: Addition Reactions of Ozone. *J. Phys. Chem. A* **2009**, *113*, 5786–5799, PMID: 19374412.

(S32) Manna, D.; Martin, J. M. L. What Are the Ground State Structures of C20 and C24? An Explicitly Correlated Ab Initio Approach. *J. Phys. Chem. A* **2016**, *120*, 153–160, PMID: 26654916.

(S33) Johnson, E. R.; Mori-Sánchez, P.; Cohen, A. J.; Yang, W. Delocalization errors in

density functionals and implications for main-group thermochemistry. *J. Chem. Phys.* **2008**, *129*, 204112.

(S34) Neese, F.; Schwabe, T.; Kossmann, S.; Schirmer, B.; Grimme, S. Assessment of Orbital-Optimized, Spin-Component Scaled Second-Order Many-Body Perturbation Theory for Thermochemistry and Kinetics. *J. Chem. Theory Comput.* **2009**, *5*, 3060–3073, PMID: 26609985.

(S35) Steinmann, S. N.; Csonka, G.; Corminboeuf, C. Unified Inter- and Intramolecular Dispersion Correction Formula for Generalized Gradient Approximation Density Functional Theory. *J. Chem. Theory Comput.* **2009**, *5*, 2950–2958, PMID: 26609976.

(S36) Krieg, H.; Grimme, S. Thermochemical benchmarking of hydrocarbon bond separation reaction energies: Jacob's ladder is not reversed! *Mol. Phys.* **2010**, *108*, 2655–2666.

(S37) Yu, L.-J.; Karton, A. Assessment of theoretical procedures for a diverse set of isomerization reactions involving double-bond migration in conjugated dienes. *Chem. Phys.* **2014**, *441*, 166–177.

(S38) Schwabe, T.; Grimme, S. Double-hybrid density functionals with long-range dispersion corrections: higher accuracy and extended applicability. *Phys. Chem. Chem. Phys.* **2007**, *9*, 3397–3406.

(S39) Huenerbein, R.; Schirmer, B.; Moellmann, J.; Grimme, S. Effects of London dispersion on the isomerization reactions of large organic molecules: a density functional benchmark study. *Phys. Chem. Chem. Phys.* **2010**, *12*, 6940–6948.

(S40) Sure, R.; Hansen, A.; Schwerdtfeger, P.; Grimme, S. Comprehensive theoretical study of all 1812 C60 isomers. *Phys. Chem. Chem. Phys.* **2017**, *19*, 14296–14305.

(S41) Zhao, Y.; Lynch, B. J.; Truhlar, D. G. Multi-coefficient extrapolated density functional

theory for thermochemistry and thermochemical kinetics. *Phys. Chem. Chem. Phys.* **2005**, *7*, 43–52.

(S42) Zhao, Y.; González-García, N.; Truhlar, D. G. Benchmark Database of Barrier Heights for Heavy Atom Transfer, Nucleophilic Substitution, Association, and Unimolecular Reactions and Its Use to Test Theoretical Methods. *J. Phys. Chem. A* **2005**, *109*, 2012–2018, PMID: 16833536.

(S43) Guner, V.; Khuong, K. S.; Leach, A. G.; Lee, P. S.; Bartberger, M. D.; Houk, K. N. A Standard Set of Pericyclic Reactions of Hydrocarbons for the Benchmarking of Computational Methods: The Performance of ab Initio, Density Functional, CASSCF, CASPT2, and CBS-QB3 Methods for the Prediction of Activation Barriers, Reaction Energetics, and Transition State Geometries. *J. Phys. Chem. A* **2003**, *107*, 11445–11459.

(S44) Ess, D. H.; Houk, K. N. Activation Energies of Pericyclic Reactions: Performance of DFT, MP2, and CBS-QB3 Methods for the Prediction of Activation Barriers and Reaction Energetics of 1,3-Dipolar Cycloadditions, and Revised Activation Enthalpies for a Standard Set of Hydrocarbon Pericyclic Reactions. *J. Phys. Chem. A* **2005**, *109*, 9542–9553, PMID: 16866406.

(S45) Dinadayalane, T. C.; Vijaya, R.; Smitha, A.; Sastry, G. N. Diels-Alder Reactivity of Butadiene and Cyclic Five-Membered Dienes ((CH)4X, X = CH2, SiH2, O, NH, PH, and S) with Ethylene: A Benchmark Study. *J. Phys. Chem. A* **2002**, *106*, 1627–1633.

(S46) Grimme, S.; Steinmetz, M.; Korth, M. How to Compute Isomerization Energies of Organic Molecules with Quantum Chemical Methods. *J. Org. Chem.* **2007**, *72*, 2118–2126, PMID: 17286442.

(S47) Karton, A.; O'Reilly, R. J.; Chan, B.; Radom, L. Determination of Barrier Heights for Proton Exchange in Small Water, Ammonia, and Hydrogen Fluoride Clusters

with G4(MP2)-Type, MPn, and SCS-MPn Procedures—A Caveat. *J. Chem. Theory Comput.* **2012**, *8*, 3128–3136, PMID: 26605724.

(S48) Karton, A.; O'Reilly, R. J.; Radom, L. Assessment of Theoretical Procedures for Calculating Barrier Heights for a Diverse Set of Water-Catalyzed Proton-Transfer Reactions. *J. Phys. Chem. A* **2012**, *116*, 4211–4221, PMID: 22497287.

(S49) Grimme, S.; Antony, J.; Ehrlich, S.; Krieg, H. A consistent and accurate ab initio parametrization of density functional dispersion correction (DFT-D) for the 94 elements H-Pu. *J. Chem. Phys.* **2010**, *132*, 154104.

(S50) Jurečka, P.; Šponer, J.; Černý, J.; Hobza, P. Benchmark database of accurate (MP2 and CCSD(T) complete basis set limit) interaction energies of small model complexes, DNA base pairs, and amino acid pairs. *Phys. Chem. Chem. Phys.* **2006**, *8*, 1985–1993.

(S51) Řezáč, J.; Riley, K. E.; Hobza, P. S66: A Well-balanced Database of Benchmark Interaction Energies Relevant to Biomolecular Structures. *J. Chem. Theory Comput.* **2011**, *7*, 2427–2438, PMID: 21836824.

(S52) Bryantsev, V. S.; Diallo, M. S.; van Duin, A. C. T.; Goddard, W. A. Evaluation of B3LYP, X3LYP, and M06-Class Density Functionals for Predicting the Binding Energies of Neutral, Protonated, and Deprotonated Water Clusters. *J. Chem. Theory Comput.* **2009**, *5*, 1016–1026, PMID: 26609610.

(S53) Setiawan, D.; Kraka, E.; Cremer, D. Strength of the Pnicogen Bond in Complexes Involving Group Va Elements N, P, and As. *J. Phys. Chem. A* **2015**, *119*, 1642–1656, PMID: 25325889.

(S54) Kozuch, S.; Martin, J. M. L. Halogen Bonds: Benchmarks and Theoretical Analysis. *J. Chem. Theory Comput.* **2013**, *9*, 1918–1931, PMID: 26583543.

(S55) Řezáč, J.; Riley, K. E.; Hobza, P. Benchmark Calculations of Noncovalent Interactions of Halogenated Molecules. *J. Chem. Theory Comput.* **2012**, *8*, 4285–4292, PMID: 26605592.

(S56) Lao, K. U.; Schäffer, R.; Jansen, G.; Herbert, J. M. Accurate Description of Intermolecular Interactions Involving Ions Using Symmetry-Adapted Perturbation Theory. *J. Chem. Theory Comput.* **2015**, *11*, 2473–2486, PMID: 26575547.

(S57) Grimme, S. Seemingly Simple Stereoelectronic Effects in Alkane Isomers and the Implications for Kohn–Sham Density Functional Theory. *Angew. Chem., Int. Ed.* **2006**, *45*, 4460–4464.

(S58) Grimme, S.; Steinmetz, M.; Korth, M. How to Compute Isomerization Energies of Organic Molecules with Quantum Chemical Methods. *J. Org. Chem.* **2007**, *72*, 2118–2126, PMID: 17286442.

(S59) Kesharwani, M. K.; Karton, A.; Martin, J. M. L. Benchmark ab Initio Conformational Energies for the Proteinogenic Amino Acids through Explicitly Correlated Methods. Assessment of Density Functional Methods. *J. Chem. Theory Comput.* **2016**, *12*, 444–454, PMID: 26653705.

(S60) Fogueri, U. R.; Kozuch, S.; Karton, A.; Martin, J. M. The Melatonin Conformer Space: Benchmark and Assessment of Wave Function and DFT Methods for a Paradigmatic Biological and Pharmacological Molecule. *J. Phys. Chem. A* **2013**, *117*, 2269–2277, PMID: 23379303.

(S61) Csonka, G. I.; French, A. D.; Johnson, G. P.; Stortz, C. A. Evaluation of Density Functionals and Basis Sets for Carbohydrates. *J. Chem. Theory Comput.* **2009**, *5*, 679–692, PMID: 26609572.

(S62) Kruse, H.; Mladek, A.; Gkionis, K.; Hansen, A.; Grimme, S.; Sponer, J. Quantum

Chemical Benchmark Study on 46 RNA Backbone Families Using a Dinucleotide Unit. *J. Chem. Theory Comput.* **2015**, *11*, 4972–4991, PMID: 26574283.

(S63) Kozuch, S.; Bachrach, S. M.; Martin, J. M. Conformational Equilibria in Butane-1,4-diol: A Benchmark of a Prototypical System with Strong Intramolecular H-bonds. *J. Phys. Chem. A* **2014**, *118*, 293–303, PMID: 24328111.
